# Correlated velocity models as a fundamental unit of animal movement: synthesis and applications

**DOI:** 10.1186/s40462-017-0103-3

**Published:** 2017-05-10

**Authors:** Eliezer Gurarie, Christen H. Fleming, William F. Fagan, Kristin L. Laidre, Jesús Hernández-Pliego, Otso Ovaskainen

**Affiliations:** 1 Department of Biology, University of Maryland, College Park, MD, 20742 USA; 2 Conservation Ecology Center, Smithsonian Conservation Biology Institute, Front Royal, VA, USA; 3 Polar Science Center, Applied Physics Laboratory, University of Washington, Seattle, 98195 WA USA; 4 Department of Wetland Ecology, Estación Biológica de Doñana (EBD-CSIC), c/ Américo Vespucio s/n, Seville, 41092 Spain; 5 Department of Biosciences, University of Helsinki, Helsinki, 00014 Finland; 6 Centre for Biodiversity Dynamics, Department of Biology, University of Science and Technology, Trondheim, N-7491 Norway

**Keywords:** Correlated velocity movement, Velocity autocovariance function, Correlated random walk, Integrated Ornstein-Uhlenbeck process, *Balaena mysticetus*, Thermal soaring, *Falco naumanni*

## Abstract

**Background:**

Continuous time movement models resolve many of the problems with scaling, sampling, and interpretation that affect discrete movement models. They can, however, be challenging to estimate, have been presented in inconsistent ways, and are not widely used.

**Methods:**

We review the literature on integrated Ornstein-Uhlenbeck velocity models and propose four fundamental correlated velocity movement models (CVM’s): random, advective, rotational, and rotational-advective. The models are defined in terms of biologically meaningful speeds and time scales of autocorrelation. We summarize several approaches to estimating the models, and apply these tools for the higher order task of behavioral partitioning via change point analysis.

**Results:**

An array of simulation illustrate the precision and accuracy of the estimation tools. An analysis of a swimming track of a bowhead whale (*Balaena mysticetus*) illustrates their robustness to irregular and sparse sampling and identifies switches between slower and faster, and directed vs. random movements. An analysis of a short flight of a lesser kestrel (*Falco naumanni*) identifies exact moments when switches occur between loopy, thermal soaring and directed flapping or gliding flights.

**Conclusions:**

We provide tools to estimate parameters and perform change point analyses in continuous time movement models as an R package (smoove). These resources, together with the synthesis, should facilitate the wider application and development of correlated velocity models among movement ecologists.

**Electronic supplementary material:**

The online version of this article (doi:10.1186/s40462-017-0103-3) contains supplementary material, which is available to authorized users.

## Background

All moving organisms, from unicellular organisms to whales, display heterogeneity in behavior, with multiple movement modes serving different functions. In outlining a paradigm for movement ecology, Nathan et al. [1] argued that a unified approach to movement ecology must rely on an “elemental view of a movement track,” making an analogy to genetics and the enviably discrete and countable amino acid base pairs. Unlike a strand of DNA, however, even at the most fundamental level the movements of living organisms are extremely diverse: they can be roughly linear, tortuous, circular, directed, random, stationary or some combination of the above. Only one, nearly tautological, aspect of movement can be said to be universal: all organisms are always located somewhere in continuous space and time, and all movements therefore occur in continuous space and time.

The properties of movement data are similarly variable in accuracy, precision and resolution. At the extremes are highly resolved and regular data (e.g., video data in controlled settings), and irregularly sampled data with significant errors (e.g., ARGOS satellite data on marine organisms, which are opportunistically collected when an animal is at the surface). Improved portability of power, ever more novel biologging technology, and ubiquity of coverage are yielding data that are increasingly precise and sampled at ever-higher temporal resolutions.

Given the increasing resolution of data and the intrinsic continuous nature of the movement process, one might suppose that the dominant paradigm for movement modeling would be continuous. However, the most commonly used models of animal movements are still discrete [2, 3]. In particular, the correlated random walk (CRW), first proposed by Patlak [4] and reintroduced by Kareiva and Shigesada [5], models observed location data in terms of distributions for step lengths and turning angles. The observed axial persistence of most movements (at some unspecified scale) is modeled with a parameter that quantifies the extent to which turning angles cluster around zero degrees. In most applications of CRWs, consecutive turning angles and step-lengths are assumed to be independent [2, 6] (though see [7]).

In many cases, a discrete movement model is a natural choice, for example in Shigesada and Kareiva’s earliest application to flights of butterflies between flowers [5], mostly linear elk movements between feeding craters in winter [8], or daily stop-overs during a bird’s migration. However, when the CRW is applied to raw telemetry data it becomes a model of a sampling from a continuous movement process. This can be problematic for several reasons. First, the parameters – usually, a shape and scale parameter for step lengths and a clustering parameter of turning angles – have no clear interpretation. At different discretizations (or subsamplings, or interpolations), the parameters have different values: the higher the temporal resolution, the less skewed the step lengths and the higher the clustering of the turning angles, with no simple scaling relationships (though see [3, 9]). This ambiguity reflects the fact that step-length and turning angle distributions do not capture a fundamental, biological property of continuous movement, but are artifacts of the sampling resolution, much as an estimated fractal dimension of a path is an artifact of the sampling rate [10]. The CRW is also problematic for irregularly sampled data (e.g. data from marine satellite telemetry [11, 12]), in which case movement data must be either thinned or interpolated [6]. Finally, and perhaps most importantly, for high resolution data the common assumption of serial independence is certain to be incorrect, with important consequences for false inference [2, 6].

In principle, continuous-time movement models resolve these drawbacks because they can be defined in terms of scale-invariant parameters and can be estimated regardless of sampling [3, 12, 13]. The closest continuous time equivalent to simple CRW models are ones in which velocities are modeled as two-dimensional Ornstein-Uhlenbeck (OU) processes, essentially continuous time equivalents of first order auto-regressive time series. It is straightforward to incorporate directional bias [3, 12, 14] or rotational tendencies to these models [14, 15], capturing additional, potentially important, features. These models, which we have referred to collectively as *correlated velocity models* (CVM’s, [9, 16]) have been described and applied for animal (or sub-organismal) movements for several decades. More recently, tools have been developed to estimate parameters of continuous time models, notably the “continuous time correlated random walk” of [12] and the “continuous time movement models” [13, 17] (and, respectively, the crawl [18] and ctmm [19] R packages). Nonetheless, the use of continuous time movement models has been limited in the broader movement ecology community, in part because of the unfamiliar nature of the stochastic differential equations that underlie the models, inconsistencies in how the models have been presented in the literature, and the unclear biological interpretation of some of the parameters.

Our primary goal in this paper is to argue for the flexibility and appropriateness of CVM models as a “fundamental unit” of movement. To that end, we first present and review the literature on integrated OU velocity models, all fundamentally similar but parameterized in divergent and possibly confusing ways. We propose a unifying, hierarchical family of CVM models defined in terms of biologically intuitive parameters, notably speeds and characteristic time scales. We review the statistical properties of these models and present several approaches to estimating the parameters, providing examples both for simulated data and the highly irregularly sampled track of a bowhead whale (*Balaena mysticetus*).

The estimation of a few parameters to characterize a homogeneous section of movement track is only the starting point of an analysis. As fundamental units of movement, CVM’s can serve as a basis for higher level analysis of movement tracks. We illustrate this by focusing on the problem of identifying multiple behavioral modes [20, 21], an important exploratory step with respect to higher-level questions related to energetics, time budgeting, responses to environmental cues and habitat use, and mechanisms of navigation. Widely used tools for behavioral partitioning include behavioral change point analysis (BCPA [11, 22]), the Bayesian partitioning of movement models (BPMM [23]), and analyses of first-passage and residence times (FPT, RT [24, 25]). Of these tools, only the BCPA is explicitly designed to be robust to irregularly sampled data. However, none of the tools provide a parameterized movement process as an outcome [20], as all analyze some derived statistics of the movement process. Discrete movement models have a broad range of applications as a basis for more complex models of behavior, including behavioral switching [26], step-selection (i.e. tactic responses to environmental covariates) [27], biased movements to unknown centers of attraction [28] and inferring movement processes from data with error [7, 29].

Here, we develop a method for the behavioral partitioning of movement tracks based on estimating shifts in the parameters and type of CVM. In this way complex (and arbitrarily sampled) movement tracks can be estimated in terms of biologically meaningful parameters. We perform the partitioning on the bowhead whale data, identifying transitions from exploratory movements to intensive foraging. We also partition a highly detailed portion of the lesser kestrel (*Falco naumanni*) flight track, identifying moments at which the flight transitions from autocorrelated random movement to advective movement to loopy thermal soaring, including changes between clockwise and counterclockwise rotations. To facilitate the adoption of continuous time movement models by ecologists, we provide an R package (smoove) for estimating the CVM models and performing the behavioral partitioning.

## Methods

### General formulation and review

The correlated velocity movement models we discuss in this paper can all be expressed as a continuous stochastic model for velocity **v**(*t*) that is integrated to obtain the position in time: $\mathbf {z}(t) = \mathbf {z}(0)+\int _{0}^{t} \mathbf {v}(t')dt'$. A simple continuous-time velocity model is a multivariate Ornstein-Uhlenbeck (OU) process [30]. In two-dimensions, this model is formulated most generally as the stochastic differential equation

1 $$\begin{array}{@{}rcl@{}} d\mathbf{v} = \alpha ({\boldsymbol{\mu}} - \mathbf{v}) \, dt + \beta \,d\mathbf{w}_{t} \end{array} $$

where *α* is a parameter that captures both the relaxation time (i.e. autocorrelation time scale) and a possible rotational component, ***μ*** is the asymptotic expected mean of the velocity (typically a constant 2D vector, or 0), *β* is the magnitude of the stochasticity and *d* **w**_*t*_ is a two-dimensional independent Gaussian perturbation with variance *dt*.^1^ Note that the *d* **w**_*t*_ term has units of time ^1/2^. In order to give the entire expression consistent units of velocity, the *β* parameter must have units of distance × time ^−3/2^, a unit with no clear biological interpretation. Essentially, this model describes a velocity process that is continuously fluctuating while attempting to relax to a velocity ***μ*** at some rate related to *α*. The position, in turn, is the integral of the velocity and therefore a smooth random process that is well-defined and differentiable in continuous time.

With some variations (and a variety of acronyms), an integrated OU velocity process has been used to model a wide array of animal movements. In the earliest such application we are aware of, Dunn and Brown [32], proposed the model as a fundamental one for movements of cells and noted the equivalence of this model to a first order autoregressive moving average (ARMA(1,1)) model, commonly used in time-series analysis. Alt [15] introduced complex number notation to the Dunn-Brown model, elegantly introducing rotation and advection, and discussed the predicted velocity autocovariance function of these models. Gurarie et al. [14] extended the formulation of [15] to model and estimate three-dimensional helical trajectories of a motile alga (*Heterosigma akashiwo*). Zattara et al. [33] applied the unbiased model the characterize the movements of cells in regenerating annelids. Brillinger and Stewart [34] modelled the movements of an elephant seal (*Mirounga angustirostris*) by modifying the integrated Ornstein-Uhlenbeck model for the surface of a sphere and incorporating points of attraction. Johnson et al. [12] presented this model as the *continuous time correlated random walk model* (CTCRW), provided likelihood estimates of the parameters and developed an efficient Kálmán filter-based method for estimating the parameters. Their formulation also allowed for the inclusion of advection and the separation of the underlying process from observation errors in a state-space modeling framework. The study organisms motivating their study were, again, pinnipeds: northern fur seals (*Callorhinus ursinus*) and harbor seals (*Phoca vitulina*). Fleming et al. [13] introduced and estimated an autocorrelated model that hybridizes a spatial OU process with a velocity OU process (the OUF process), such that the unbiased (***μ***=0) CVM is a special case in which the spatial time scale of autocorrelation approaches infinity [17].

We summarize these models and their applications in Table 1. Note that there is considerable (and potentially confusing) variability in the way models are parameterized. For example, the *α* in [32], *β* in [34], and *σ* in [12] all refer to the same quantity (denoted *β* in Eq. 1, with the same awkward units), while each of those symbols refers to something else entirely in other formulations.

**Table 1:** Summary of studies that develop or apply versions of the integrated Ornstein-Uhlenbeck process for modeling biological movement. Numbers 3 [34] and 5 [9] are extensions to spherical coordinates and for three-dimensional helical movement, respectively. The remaining models all correspond to one of more of the CVM family of models presented here

	Authors	Nomenclature	Parameterization	Application	Comments
1	Dunn and Brown 1987 [32]		*α* - white noise spectrum	General cell motility	
			*β* - relaxation term		
2	Alt 1990 [15]		*T* - persistence time	Unicellular organisms and individual cells	
			*ω* - mean angular speed		
			*μ* - mean advective speed		
			$\sigma _{V}^{2}$ - variance of velocity		
3	Brillinger and Stewart 1998 [34]	-	*β* - dynamical friction	Elephant seal (*Mirounga angustirostris*)	Spherical coordinates
			*σ* - Brownian motion variance term		
			*δ*- speed of attraction to center		
4	Johson et al. 2008 [12]	Continuous Time	*γ* - drift term	Northern fur seal (*Callorhinus ursinus*)	R package crawl [18] with state-
	McClintock et al. 2014 [3]	Correlated Random Walk	*β* - autocorrelation parameter	Harbor seal (*Phoca vitulina*)	space observation error
		(CTCRW)	*σ* - white noise variance term		
5	Gurarie et al. 2011 [14]	Correlated Velocity	*τ*_*a*_,*τ*_*o*_ - characteristic time scales ^∗^	Dinoflaggelate (*Heterosigma akashiwo*)	[15] adapted to 3D helical movement:
		Helical Movement	*σ*_*a*_,*σ*_*o*_ - white noise term variance ^∗^		* - *a* and *o* refer to advective and
		(CVHM)	***μ*** - 3-D mean velocity		oscillatory components, respectively)
			*ω* - mean angular speed		
6	Gurarie and Ovaskainen 2011 [9]	Correlated Velocity	*τ* - characteristic time scale	Encounter rate theory	
	Gurarie and Ovaskainen 2013 [16]	Movement (CVM)	*σ* - characteristic spatial scale		
			*ν* - mean tangential speed		
7	Zattara et al. 2016 [33]	CVM	*τ* and *ν* as above	Regenerating cells in *Pristina leidyi*	
8	Calabrese et al. 2016 [17]	Integrated Ornstein-	*τ*_*v*_ - time scale of velocity autocorrelation	Presented as limiting case of OUF model [13]	R package ctmm [19]
		Uhlenbeck (IOU)			

It is noteworthy that integrated Ornstein-Uhlenbeck velocity process have mainly been applied either to microorganisms videotaped in laboratory settings or to marine mammals traveling over spatial scales of hundreds or thousands of kilometers. These applications – near the absolute extremes of the scales at which organisms move – reflect two advantages of continuous time movement models: their explicit ability to deal with highly autocorrelated data sampled at high temporal resolution (e.g. videography), and their ability to handle irregularly sampled data, typical for marine telemetry where locations can only be obtained when the animal is (unpredictably) at the surface.

### Four fundamental models

We present here a consolidation of integrated OU velocity models into a unified, hierarchically structured family of CVM’s formulated in terms of biologically meaningful parameters. All of the models are special cases of the general process in Eq. 1. We describe these models qualitatively here, summarizing the notation and parameters in Table 2, with more details provided in Additional file 1: Appendix A.

**Table 2:** Notation, parameters, units and derived properties of correlated velocity movement models

Model	Parameter (units)	Mean speed	Mean squared speed	Velocity auto-covariance function
	*τ* - characteristic time scale (time)			
Unbiased CVM				
UCVM(*τ*,*ν*)	*ν* - mean speed (dist/time)	*ν*	${2 \over \pi } \nu ^{2} $	${2 \nu ^{2} \over \pi } e^{-{\Delta t \over \tau }}$
UCVM(*τ*,*η*)	*η* - random rms speed (dist/time)	$\sqrt {\pi \over 2} \eta $	*η* ^2^	${\eta ^{2}} e^{-{\Delta t \over \tau }}$
Advective CVM				
ACVM(*τ*,*η*,***μ***)	***μ*** - advective velocity (2D - dist/time)	eq. A6	*η* ^2^+|***μ***|^2^	$|\boldsymbol {\mu }|^{2} + \eta ^{2} e^{-{\Delta t \over \tau }} $
Rotational CVM				
RCVM(*τ*,*η*,*ω*)	*ω* - angular speed (radians/time)	-	*η* ^2^	$\eta ^{2} e^{-{\Delta t \over \tau }} \cos (\omega \Delta t)$
Rotational-advective CVM				
RACVM(*τ*,*η*,*ω*,***μ***)		-	*η* ^2^+|***μ***|^2^	$|\boldsymbol {\mu }^{2}| + \eta ^{2} e^{-{\Delta t \over \tau }} \cos (\omega \Delta t)$

**Fig. 1:**
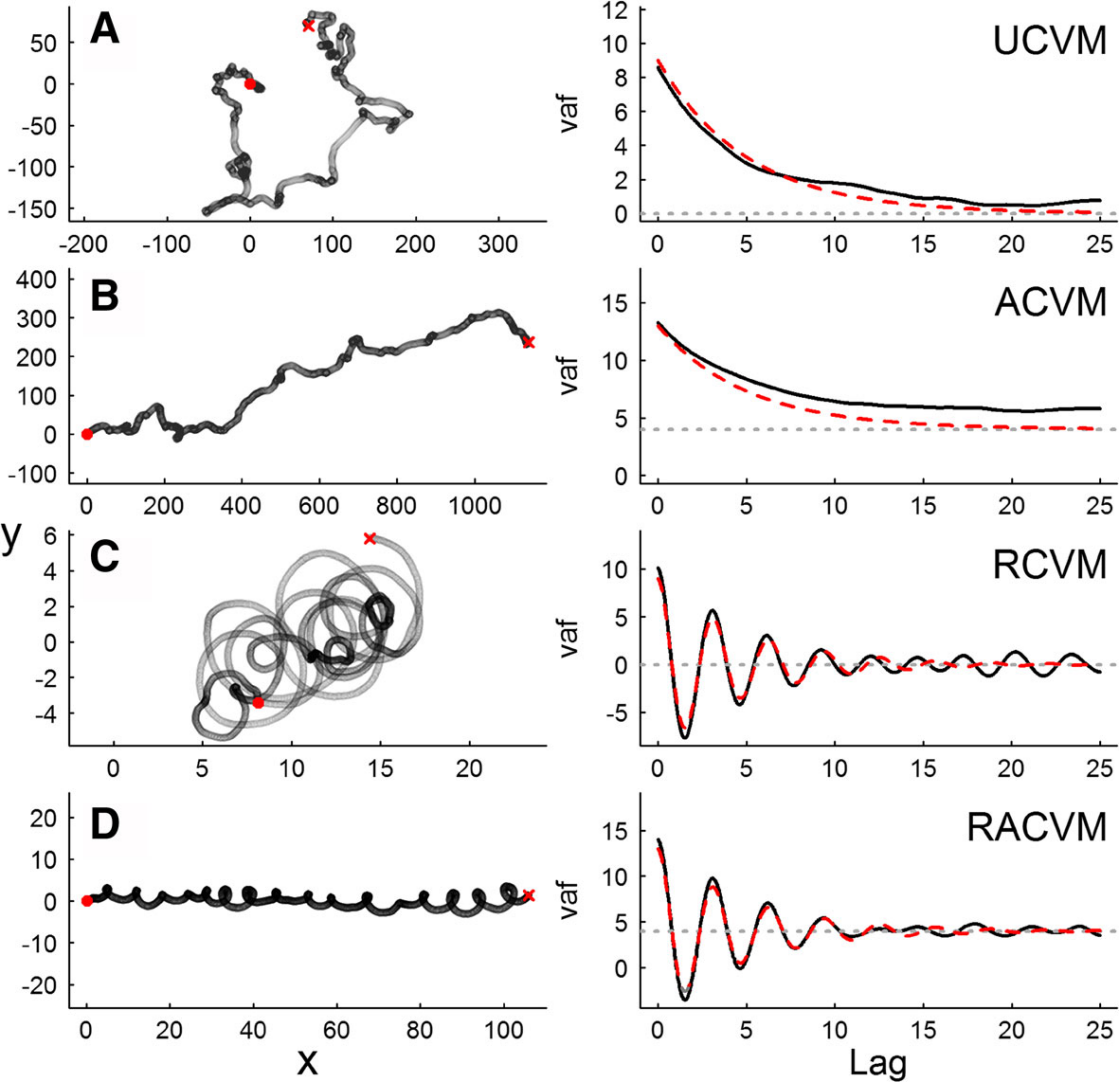
Four sample trajectories (*left* panels) and corresponding velocity auto-covariance functions (*right* panels) of CVM movement models. In all trajectories, the characteristic time scale *τ*=5, the random mean squared speed *η*=3 and the sampling intervals are 0.01. Start and end of each trajectory is represented with filled circles and x’s, respectively. Regions of *darker* and *lighter grey* within the track indicate locations where the speed is slower or faster. In panel **a**, the mean velocity and rotation are equal to 0, in panels **b** and **d**, there is a mean component of velocity *μ*_*x*_=2, and in panels **c** and **d** there is a rotational component *ω*=2. In the *right* panels, *black lines* are the empirical estimates of the velocity auto-covariance function (EVAF), the *red dashed line* is the theoretical prediction (Equation A15), and the *horizontal dashed grey line* is the predicted asymptote |***μ*** ^2^|, reflecting the advective term in the process

**Unbiased correlated velocity model (UCVM):** The unbiased CVM (Fig. 1 a) is a continuous time analogue of an unbiased CRW. It is obtained by making the substitutions $\alpha = {1 \over \tau }$ and $\beta = {2 \nu \over \sqrt {\pi \tau }}$:

2 $$\begin{array}{@{}rcl@{}} d\textbf{v} &=&{1 \over \tau}\textbf{v} \, dt + {2 \nu\over \sqrt{\pi\tau}} \,d\textbf{w}_{t} \end{array} $$

In this formulation, *ν* simply represents the mean actual speed of movement and *τ* is a characteristic time scale of auto-correlation [12, 14, 15]. We refer to this model as UCVM(*τ*,*ν*). As *τ*→0, the UCVM approaches uncorrelated random Brownian motion. As *τ*→*∞* the UCVM approaches perfectly linear motion. Thus, the UCVM is a simple, two parameter model that spans the entire range of possible tortuosities and speeds. An alternative parameterization of the UCVM is in terms of the root mean squared speed $\eta = {2 \nu \over \sqrt {\pi }}$, also a useful measure (as seen below).

**Advective CVM (ACVM):** The ACVM (Fig. 1 b) has a mean non-zero advective velocity ***μ*** (where the bold-facing represents a two-dimensional vector), and is usefully expressed as ACVM(*τ*,*η*,***μ***). The *mean squared speed* of an ACVM process is tidily decomposed into the random and advective components: $|\overline {\textbf {v}^{2}}| = \eta ^{2} + |\boldsymbol {\mu }|^{2}$, where the vertical bars indicate the magnitude of the advective velocity: $|\boldsymbol {\mu }|^{2} = \mu _{x}^{2} + \mu _{y}^{2}$. Note that with the random r.m.s. speed, the time scale, and the two components of the advective velocity, this model is specified by four parameters.

**Rotational CVMs (RCVM and RACVM):** It is similarly straightforward to introduce a rotational component to the CVM [9, 15] by substituting a two by two matrix: $\left [ \begin {array}{cc} {1 / \tau } & -\omega \\ \omega & {1 / \tau } \\ \end {array} \right ]$for the *α* parameter in Eq. 1. This term combines the decay to the mean along the main coordinates with a nudge that is perpendicular to the direction of movement. With no advection, we denote this model RCVM(*τ*,*η*,*ω*) where *ω* is a mean radial velocity (rotations × time ^−1^). With advection, the model is denoted RACVM(*τ*,*η*,*ω*,***μ***) (Fig. 1 c and d). A characteristic spatial scale of rotation can be defined as *ρ*=*η*/*ω*, i.e. the ratio between the random speed component and the angular speed, which is closely related to the circling radius used to characterize helical soaring flights [35]. With somewhat different parameterizations, rotational and advective-rotational models have been analyzed to model cellular movement [15] and helical trajectories of unicellular algae [14].

Decomposed into *x* and *y* components, the complete RACVM model is expressed:

3 $$\begin{array}{*{20}l} dv_{x} &= \left({1\over \tau}(\mu_{x} - v_{x}) - \omega(\mu_{y} - v_{y})\right)dt + {\eta \over \sqrt{\tau}} dw_{x,t} \\ dv_{y} &= \left({1\over \tau}(\mu_{y} - v_{y}) + \omega(\mu_{x} - v_{x})\right)dt + {\eta \over \sqrt{\tau}} dw_{y,t}  \end{array} $$

Setting *μ*_*x*_=*μ*_*y*_=0 gives the RCVM, setting *ω*=0 gives the ACVM, and setting *μ*_*x*_=*μ*_*y*_=*ω*=0 gives the UCVM (with the *η* parameterization).

It should be noted that all of these processes are conditioned on an initial velocity **v**_0_. If the initial velocity is “extreme”, the process needs some time (governed by the magnitude of *τ*) to settle into its asymptotically stationary behavior. The initial speed parameter is of little biological interest in practice, as we typically assume that a sampled CVM is already in its stationary state (see Additional file 1: Appendix C.3 for more details).

### Statistical properties

Key statistical properties (expectations, variances, autocorrelations) of the CVM processes are summarized in Additional file 1: Appendix B. These properties directly inform the estimation procedures.

In brief, both the position and velocity are Gaussian, with the long-term mean of the location (|**z**(*t*≫*τ*)|) equal to the initial location |**z**(0)| for the non-advective UCVM and RCVM, and equal to the advective velocity times time ***μ*** *t* for the ACVM and RACVM. At long time frames (*t*≫*τ*), the variance of the process increases linearly with time (as for any unconstrained random movement) in proportion to the random mean squared speed *η* ^2^. This linearly increasing variance is characteristic of unconstrained random walks [9, 16]. At intermediate time ranges, both the positions and velocities are correlated with magnitudes controlled by the time scale parameter *τ*.

### Velocity autocovariance functions

A useful measure for visualizing the structure of CVM processes is the velocity autocovariance function (VAF [9, 15, 36]). Defined as the expected dot product of the velocity vectors over different lags, the VAF is directly analogous of the familiar autocovariance function for discrete one-dimensional time series. Theoretical VAF’s of the four CVM processes have convenient and simple expressions (Table 2). At lag zero, they are all equal to |**v** ^2^|. At increasing lags they decay exponentially with rate 1/*τ* to |***μ***|^2^. Rotational processes contain an additional oscillatory component with frequency *ω*.

Empirical velocity autocovariance functions (EVAF’s) can be computed by taking means of observed dot products across lags. Because of its intrinsic smoothing, the EVAF provides a useful visual exploratory tool for recognizing these fundamental processes (Fig. 1, right panels). There is a strong analogy between analysis of velocity autocovariance and the use of variograms for position data [17, 37]: the variogram similarly smooths across time lags, and known theoretical forms of the curve are used to identify fundamental ranging processes. The key difference between the two tools – that one is based on velocities while the other is based on positions – suggests an important caveat for the application of VAF’s, namely that VAF’s are obtainable only for data that is sufficiently high resolution (i.e. *Δ* *T*≪*τ*, where *Δ* *T* is a time interval). Inferring the VAF from irregular position data is a topic for future work.

### Estimation methods

Given the raw ingredients of movement data, a vector of 2D positions (*Z*_*i*_={*X*_*i*_,*Y*_*i*_}) and a vector of times (*T*_*i*_), there are several approaches to estimate or approximate CVM parameters. We describe these methods here in a qualitative way, referring the reader to Additional file 1: Appendix C for technical details. Broadly, there are two approaches: phenomenological methods that match some statistical property of the observed trajectory, analogous to method of moments estimation, and maximum likelihood methods that exploit the distribution of the position or velocity processes.

#### Method of moments estimators

For a movement process that is sampled relatively coarsely (i.e. the intervals between observations are approximately equal to or greater the time scale of autocorrelation), the most straightforward approximation of the UCVM parameters is to match those parameters to correlated random walk (CRW) parameters. Specifically, the UCVM speed and time scale parameters can be expressed in terms of the ratio between the variance and mean of discrete step lengths (parameter *λ*), the mean interval between observations (*Δ* *T*), and the mean cosine of the turning angles (*κ*) via a straightforward set of formulas (Additional file 1: Appendix C.1). The equations are derived from matching the characteristic movement scales of the processes [14]. Because the CRW process is not exactly equivalent to a UCVM this method is generally biased and useful only as a rough approximation for data that are coarsely sampled. The main advantage of this method is that it is very fast to compute, and can be used to translate reported CRW parameters in older studies to velocities and time-scales.

For data that are high resolution (i.e. where time scales are larger than sampling intervals), fitting EVAF curves to their theoretical predictions can be an effective way to obtain parameters for any of the four models (Fig. 1). In particular, the high-lag stabilization value of the EVAF is an excellent estimate of (the square of) advective speeds, and any rotational component is usually evident and easy to fit in the VAF. This approach was suggested by Alt [15] and applied to model helical movements of motile alga [14]. Additional details of VAF fitting, including expressions for computing the EVAF and techniques for dealing with autocorrelated residuals are provided in Additional file 1: Appendix C.2.

#### Maximum likelihood estimators

Maximum likelihood based estimates of CVM processes have many fundamental advantages over method of moment estimators. In particular, they provide tools to assess and compare models and to quantify the accuracy of an estimate. The distributions of the velocity and positions of the CVM processes catalogued in Additional file 1: Appendix B can be leveraged directly to write the likelihood of parameter values given observations.

The simplest approach to likelihood estimation is to use the estimated velocities, i.e. sequential displacements of the process divided by the time intervals: **V**_*i*_=(**Z**_*i*_−**Z**_*i*−1_)/(*T*_*i*_−*T*_*i*−1_). The conditional distribution of the velocities (i.e. **V**_*i*_|**V**_*i*−1_) is Gaussian (Additional file 1: Appendix C.3), and the joint distribution of the vector of velocities can be numerically maximized efficiently. This method works best for relatively high frequency sampling, but the data need not be regularly sampled. Directly computed velocities necessarily underestimate the true velocity of the process because they assume straight line movements between locations. This bias can be mitigated, somewhat, by an X-Y-T spline of the positions (Additional file 1: Appendix Figure A1).

Finally, it is possible to estimate the parameters using only the location data, without recourse to computed velocities (Additional file 1: Appendix C.3). In order to maximally leverage all the location data, all of the correlations across all points are included into this “full-position” maximum likelihood. A direct maximization of the likelihood is typically computationally much more intensive than the velocity likelihood. Johnson et al. [12] developed an invaluable computational method for maximizing this likelihood with the aid of a Kálmán filter (see also the crawl R package [18]). We refer the reader to the original article and the associated appendices, noting that the parameters those authors refer to as *β* and *σ* correspond in our parameterization to 1/*τ* and *η* ^2^/*τ*, respectively (Table 1).

### Change point analysis

The CVM models are flexible characterizations of movement paths controlled by a stable set of underlying parameters. The particular model and parameter values can reflect fundamental behavioral modes which serve particular functions, i.e. the *movement phases* sensu Nathan et al. [1], which might be associated with directed traveling, foraging, resting, escaping, or any other important function. It is a common and important first-order challenge when first confronting movement data to attempt to identify and quantify those fundamental phases [20, 21].

The likelihood based estimation of the CVM movement models provides a framework for implementing an exploration of movement phases using a variation of the behavioral change point analysis (BCPA) [11, 20]. The fundamental assumption behind the analysis is that a movement phase is identified either by a unique fundamental model or by significant shift in parameter values at unknown times. We enumerate the steps of the heuristic below:

1. Define a subset of the data of a certain sample size or time duration (the *window size*) and subset of CVM models which are of biological interest.
2. Find the time point within the window (the most likely change point - MLCP) for which the likelihood of fitting two models on either side of the window is maximized.
3. Record the MLCP, move the window forward some small step (the *step size*) and repeat step 2, logging the MLCP at each scan. This set of MLCP’s can be initially thinned by merging selected change points that are within some time interval (*cluster width*).
4. For the final set of candidate MLCP’s, determine the significance of a change point based on a comparison of the BIC of fitted models on either side of the candidate point to the BIC of a model with no change point. Note that a BIC based model selection process from the set of candidate CVM’s occurs within each estimation. For example, consider a case where the selected model for Phase I is ACVM, for Phase II is RCVM and for the combined movement subset is UCVM. In that case, the no-change point model has only two parameters (*ν* and *τ*) while the change point model would have 8 parameters (4 for the ACVM(*τ*,*η*,*μ*_*x*_,*μ*_*y*_), 3 for the RCVM(*η*,*τ*,*ω*), and the change point itself *t* ^∗^). The BIC analysis will identify two kinds of differences: Did the fundamental model change (e.g. did the movement switch to an advective movement from a random movement)? Or did the values of the parameter change (e.g. did the movement speed up, become more tortuous, etc.)?

The output of this analysis is a fully parameterized sequence of fundamental movement phases, together with the times (and locations) of the switches between the phases. The method does require the setting of two free parameters: the window size and the cluster width. There are no hard and fast rules for the selection of these criteria. Larger window size will mask very short phases, while shorter windows will have a harder time detecting significant changes. But for a reasonable range of values, the results will generally be consistent (see the package vignette in the Additional file 2 for a mini study of the sensitivity of the change point analysis).

Code to simulate and estimate the CVM models and perform a change point analysis is bundled in an R package called smoove, available in the Additional file 1 as well as on GitHub at: https://github.com/EliGurarie/smoove. The package vignette includes examples of simulation and estimation of homegeneous CVM processes and a step-by-step illustration of the change point analysis.

### Simulation study

To illustrate the autocorrelation structure of the velocities in the general context of the CVM models, we simulated four tracks at high resolution (500 steps at interval *Δ* *t* = 0.01): a UCVM, ACVM(*μ*_*x*_=2), RCVM(*ω*=2), and RACVM (*μ*_*x*_=2,*ω*=2) and compared the empirical autocovariance function with the theoretical predictions. We randomly sampled 400 points from the complete tracks illustrated in Fig. 1 and used the position likelihood method to obtain estimates of model parameters and to select the different CVM models with BIC.

We also performed a more comprehensive simulation experiment assessing the four estimation methods according to precision, accuracy and speed for regular and irregular, high and low resolution tracks. The setup and results of those simulations are detailed in Additional file 1: Appendix D.

### Application to bowhead whale data

We analyzed a portion of movement data of a GPS tagged female bowhead whale tracked in Disko Bay in western Greenland (inset map in Fig. 3). The tag was a Fastloc GPS retrievable data and dive logger (www.wildlifecomputers.com). The track consists of 954 locations collected between April 28 and May 21, 2008 (further details: [38]). These data were an excellent candidate for testing the methods developed here as they are highly precise (typical GPS error < 25 m), but very irregularly sampled (mean interval between locations 34.5 min, median 23 min, minimum 6 min, maximum 10 h), thereby combining several common features of telemetry data on marine organisms.

**Fig. 2:**
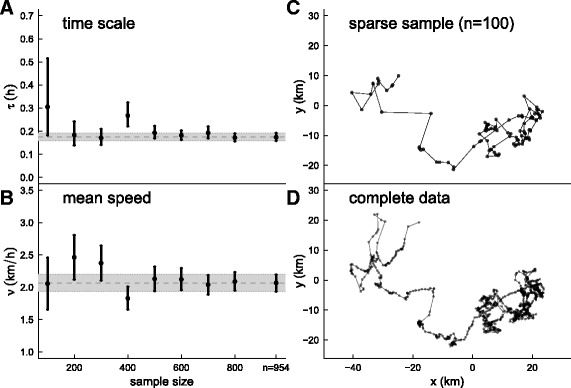
Results of estimation of UCVM parameters for the Greenland bowhead whale (see inset in Fig. 3). Panels **a** and **b** indicate the full position likelihood estimates of time scale *τ* and speed *ν* for a range of random subsamplings from 100 observations (illustrated in panel **c**) to the complete dataset with 954 observations (panel **d**) intervals for the estimates. The *vertical bars* indicate the 95% confidence interval of the estimate, while the *horizontal grey bar* shows the point estimate and confidence intervals for the compete data (i.e. *n*=954) for comparison

**Fig. 3:**
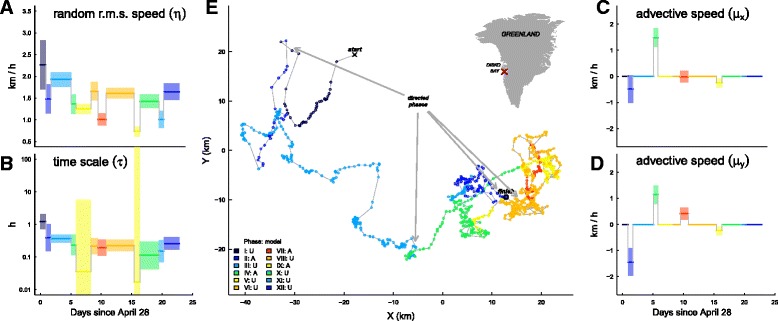
Change point analysis of the bowhead track in Disko Bay, Greenland (inset map). In the *left* panels are the estimates of (**a**) random r.m.s. speed *η* and (**b**) time scale *τ*. On the *right* panels, estimates of the (**c**) *x* and (**d**) *y* components of the advective velocity ***μ***. These are non-zero only for those four phases (II, IV, VII, IX) for which the advective CVM was selected over the unbiased CVM. Each color corresponds to a particular phase, matching the mapped track (**e**), with enumerated phases (legend in panel (**e**)) reporting whether the movement phase was determined to be unbiased (U) or advective (**a**). The *arrows* point to the first location of the four directed phases

We performed two analyses of the bowhead whale data. First, we tested the robustness of the likelihood estimation by estimating the UCVM parameters on random subsamples of the bowhead data, drawing between *n*=100 and the maximum *n*=954 observations, such that the mean sampling intervals ranged from 5.3 to 0.57 h. For each sub-sampling, we estimated the mean speed *ν* and the time scale *τ* of the movement.

Second, we performed a change point analysis of the whale’s movement, testing for unbiased and advective movements, with a window size of 50 and a cluster width of 0.5 h using the full 954 observation dataset. Additionally, we explored the consistency of the change point analysis against subsampling by repeating the analysis with 75, 50 and 25% of the data. In order to make the analyses comparable, we scaled the window size to the subsampling, using windows of 100, 75, 50, and 25 data points for the 100, 75, 50 and 25% subsampling (details in Additional file 1: Appendix G).

### Application to kestrel data

We analyzed the flight path of a lesser kestrel (*Falco naumanni*) tracked in southwestern Spain using high frequency (1 second) GPS dataloggers [39–41]. Like other birds, lesser kestrels can fly either by flapping their wings or by soaring-gliding through harvesting kinetic energy from the atmosphere. In thermal soaring, birds rise in a circular pattern when soaring on a heated thermal and then glide down to catch another thermal pocket. Thus, lesser kestrels alternate between directed flapping or gliding flight and loopy thermal soaring and the track is a complex mixture of unoriented, advective, and advective-rotational movements.

To explore the basic estimation and selection of CVM models, we analyzed the segments of a flight that were (a) clearly advective, (b) rotational-advective and (c) random, using AIC as a criterion for the model selection.

We then performed a comprehensive CVM change point analysis across the entire 7 minute kestrel flight track, identifying moments at which the bird switched between advective, rotating, rotating-advective and unbiased movements. In the change point analysis, we used a window size of 50 and a cluster width of 1 sec.

## Results

### Simulation study

Simulated high-resolution tracks (Fig. 1) have characteristically “smooth” trajectories with speeds that vary along the tracks (darker and lighter colors in the figure). The corresponding empirical and theoretical velocity autocovariance functions illustrate the main kinds of information that can be derived from their inspection: the characteristic rates of decay of autocorrelation, the mean speeds (tracks B and D) and the periodicity (tracks C and D).

For the likelihood estimation of the randomly sub-sampled tracks with *n*=400, all the true simulation parameters were within the 95% confidence intervals of the estimates (Table 3). The estimates of *τ* were the least precise, whereas the estimates of the advective bias and rotation were very precise, within a few percent of the true values. The AIC based model selection correctly selected the true model in all cases, with the strongest relative signal (greatest *Δ*AIC) for the rotational models, suggesting that it is easier to pick out a movement with consistent rotational bias than with consistent advective bias.

**Table 3:** Table of four simulation tracks (Fig. 1 - see details of data sampling in text) and three segments of the kestrel flight data (Fig. 4) with estimates and 95% confidence intervals of parameters (time scale *τ*, random rms speed *ν*, advective speed components *μ*_*x*_ and *μ*_*y*_ and angular velocity *ω*). The model selection is based on comparing BIC values for the four models while the reported estimates are only for the selected model. The kestrel portions are numbers VI, VIII and XII - orange, dark blue, and yellow portions in Fig. 1

Data	Parameter values		Model	*Δ* *B* *I* *C*
**Simulation**	True values	Estimates (C.I.)		
A	*τ*=5	4.20 (2.65 - 6.03)	**UCVM**	**0**
	*η*=3	2.19 (1.75 - 2.63)	ACVM	2.00
			RCVM	1.99
			RACVM	4.00
B	*τ*=5	6.3 (3.74 - 10.61)	UCVM	1.01
	*η*=3	2.71 (2.02 - 3.41)	**ACVM**	**0**
	*μ*_*x*_=2	1.91 (0.54 - 3.28)	RCVM	2.17
	*μ*_*y*=0	0.08 (-1.29 - 1.46)	RACVM	1.08
C	*τ*=5	7.22 (3.39 - 15.35)	UCVM	830
	*η*=3	2.85 (1.78 - 3.92)	ACVM	834
	*ω*=2	2.04 (1.94 - 2.15)	**RCVM**	**0**
			RACVM	4.79
D	*τ*=5	9.22 (5.0-17.0)	UCVM	1300
	*η*=3	3.30 (2.31 - 4.3)	ACVM	1287
	*μ*_*x*_=2	1.99 (1.89 - 2.11)	RCVM	629
	*μ*_*y*_=0	0.04 (-0.07 - 0.15)	**RACVM**	**0**
	*ω*=2	2.02 (1.95 - 2.09)		
**Kestrel**	Parameters (units)			
Segment VI	$\widehat {\tau }$ (sec)	29.1 (8.7-97.2)	**UCVM**	**0**
(*n*=68)	$\widehat {\eta }$ (m/sec)	7.7 (3.1 - 8.7)	ACVM	4.4
			RCVM	2.3
			RACVM	6.1
Segment VIII	$\widehat {\tau }$	38.9 (8.8 - 172.3)	UCVM	246
(*n*=69)	$\widehat {\eta }$	7.61 (2.02 - 13.2)	ACVM	253
	$\widehat {\mu _{x}}$ (m/sec)	2.77 (2.24 - 3.31)	RCVM	68.7
	$\widehat {\mu _{y}}$ (m/sec)	-0.87 (-1.41 - -0.33)	**RACVM**	**0**
	$\widehat {\omega }$ (rad/sec)	0.56 (0.52 - 0.60)		
Segment XII	$\widehat {\tau }$	14.4 (5.3 - 38.6)	UCVM	4.8
(*n*=31)	$\widehat {\eta }$	4.04 (2.04 - 6.05)	**ACVM**	**0**
	$\widehat {\mu _{x}}$	-10.1 (-17.6 - -2.6)	RCVM	8.0
	$\widehat {\mu _{y}}$	-8.8 (-17.3 - -0.35)	RACVM	6.8

### Whale movement analysis

The estimates for the UCVM parameters of the whale track using all *n*=954 datapoints were $\widehat {\tau }$ = 10.4 min (95% CI: 9.5-11.5) and $\widehat {\nu } = 2.07$ km/h (95% CI: 1.93-2.20). Estimates of *ν* were consistent between 1.7 and 2.6 km/h across sampling rates (Fig. 2 b). Estimates of the time scale were also mostly consistent for the random subsampling, ranging from 10.2 to 18.6 min, but tended to be somewhat higher for the more sparse subsamplings (Fig. 2 a).

The change point analysis suggested a division into 12 phases of homogeneous behavior across the 33 day track (Table 4, Fig. 3). Four of those phases (II, IV, VII, IX) were identified as advective, while the remaining eight were unbiased. The random root mean squared speed *η* tends to be lower when there is a significant advection, as expected since including a mean component of velocity absorbs some of the total magnitude of variation. For ACVM models with parameters *ν* and ***μ***, the estimated mean speed of movement is given by equation A6. We report this speed for each phase in Table 4.

**Table 4:** Table of bowhead change point analysis results (Fig. 3), presenting for each phase: the times of initiation and completion and duration, estimates of the time scale parameter *τ*, and, in the right four columns, speed estimates: the random r.m.s. speed *η*, the *x* and *y* components of the mean velocity ***μ***, and the mean tangential speed of the process *ν*. All speeds are in km/h

Phase	Model	Start	End	Duration	Time scale	Random	Advective		Mean
		(mm-dd hh:mm)		(h)	*τ*	r.m.s. *η*	*μ*_*x*_	*μ*_*y*_	*ν*
I	UCVM	04-28 18:41	04-29 16:06	21.4	1.214	2.263			2.005
II	ACVM	04-29 16:06	04-30 11:50	19.7	0.401	1.480	-0.49	-1.44	1.926
III	UCVM	04-30 11:50	05-03 20:28	80.6	0.375	1.930			1.711
IV	ACVM	05-03 20:28	05-04 14:22	17.9	0.234	1.361	1.49	1.14	2.144
V	UCVM	05-04 14:22	05-07 02:04	59.7	0.036	1.247			1.105
VI	UCVM	05-07 02:04	05-08 05:50	27.8	0.215	1.660			1.471
VII	ACVM	05-08 05:50	05-09 14:19	32.5	0.196	1.010	-0.01	0.42	0.969
VIII	UCVM	05-09 14:19	05-14 04:02	109.7	0.228	1.610			1.427
IX	ACVM	05-14 04:02	05-15 03:24	23.4	0.017	0.733	-0.25	-0.24	0.721
X	UCVM	05-15 03:24	05-18 05:58	74.6	0.114	1.430			1.267
XI	UCVM	05-18 05:58	05-19 00:40	18.7	0.154	1.005			0.891
XII	UCVM	05-19 00:40	05-21 14:26	61.8	0.263	1.647			1.460

The whale began its movement on April 28 with a fast (mean 2.01 km/h), and highly correlated random movement (*τ*=1.2 h) before switching, still at relatively high speed, to a directed southward movement for 0.8 days (Phase II, medium blue), followed by a less correlated (*τ*=0.3 h) and extended 3.3 day long Phase III (light blue) with a slow drift eastward. On May 3 (at around 18:00) it began a sudden, highly directed and rapid (mean speed 2.14 km/h) northeast transition (Phase IV, pale green color), traveling 81 km over an eighteen hour period. This was followed by, essentially, 17 days of behaviors that were highly tortuous (time scales between 0.01 and 0.22 h), variable, and relatively slow (under 1.5 km/h mean speeds). During this period, we detected two periods of directed movement towards the north and the southwest (Phases VII and IX, respectively, red and yellow colors), but these were also slow (less than 1 km/h).

The intervals between observations (mean 27 min) were generally larger than the estimates for *τ* (Table 4), requiring the use of the position likelihood to obtain reliable estimates. There were no relationships between *τ* estimates across the behavioral phases, suggesting that they represent distinct features of the whale’s behavior. Furthermore, there were no relationships between the mean sampling intervals and the estimates of *τ* and *ν* (Table 4), suggesting that within the range of the mean sampling intervals in the eight identified phases (20 to 416 min) the sampling intensity was not confounding the estimates.

Results for the subsampling validation of the bowhead change point analysis are presented in Additional file 1: Appendix G. Generally, there was good agreement between the timing of selected change points (Additional file 3: Figure A4), though that deteriorated with more sparse samples. Predicted estimates across particular subsamplings and the complete data were most correlated at the highest subsampling (*r* ^2^=0.89 for *η* and 0.80 for *τ* at the 75% subsampling), but even at a 25% subsampling agreement was high (*r* ^2^=0.86 and 0.68 for *η* and *τ*, respectively). The *η* estimates tended to be lower as sparser subsamplings, the *τ* estimates were more variable but less biased.

It is worth noting that the preprocessing of this dataset was minimal. The only data that were removed were observations at the beginning of the track with intervals shorter than 5 minutes, which were an artifact of the tagging process itself (i.e. transmitting on the vessel before deployment).

### Kestrel flight analysis

**Fig. 4:**
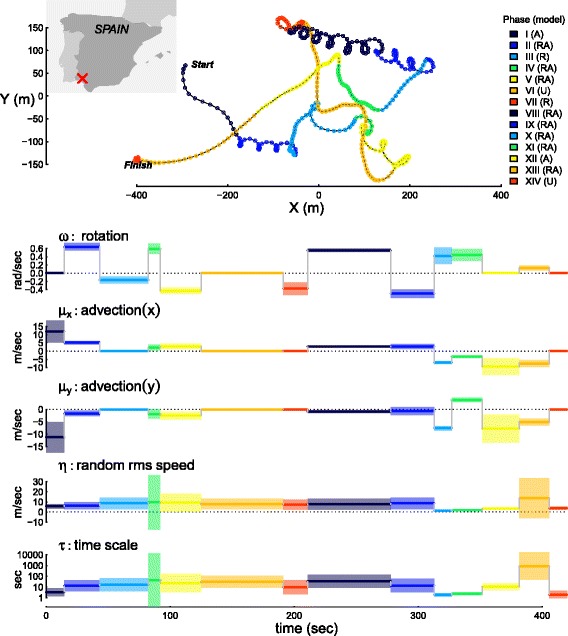
Change point analysis of a lesser kestrel’s 7 min flight in southwestern Spain (inset map). The *upper* panel illustrates the track of the flight, with the colors indicating 14 identified phases starting with the *dark blue* (phase I, at the indicated *start*) and cycling twice through high contrast rainbow colors to the final *red* roost (phase XIV, *finish*). The legend indicates whether a particular portion of the track contained a significant advective (**a**) or rotational component(R), both (RA), or neither (U). The *lower* panels indicate the estimated values of the five RACVM parameters for each phase over time, with the width of the bars indicating 95% confidence intervals. Note that positive and negative values for *ω* represent clockwise and counterclockwise rotation, respectively, and values of 0 for *ω*, *μ*_*x*_ and *μ*_*y*_ indicate that a non-rotational and/or advective model was selected

The multi-model change point analysis broke the 7 minute kestrel flight into 14 phases varying in duration between 10 and 67 seconds (Fig. 4, Table 5). According to the partitioning, the kestrel spent 56% of this flight engaged in rotational advective movement (RACVM), likely associated with thermal soaring. The partition identified moments when the kestrel switched behaviors, e.g. from directed flight (flapping or gliding) to soaring on a thermal (phases I and II, Fig. 4), or within a thermal soaring event between rotating clockwise and counter-clockwise (e.g. two transitions between phases VII, VIII and IX).

**Table 5:** Table of kestrel change point analysis results (Fig. 4). The data were collected at 1 second intervals for 7 minutes. The random r.m.s. (*η*) and advective speeds (*μ*_*x*_, *μ*_*y*_) are in m/sec

Phase	Model	Duration (sec)	*τ* (sec)	*η*	*μ*_*x*_	*μ*_*y*_	*ω* (sec ^−1^)
I	ACVM	14.5	3.43	5.62	11.9	-11.29	
II	RACVM	28.5	13.01	6.25	5.15	-1.65	0.64
III	RCVM	39.4	16.63	8.67			-0.16
IV	RACVM	9.6	44.1	9.57	2.23	-1.81	0.59
V	RACVM	32.83	23.61	9.21	2.9	-2.46	-0.43
VI	UCVM	66.17	30.49	8.03			
VII	RCVM	19.86	9.58	7.23			-0.37
VIII	RACVM	67.02	33.99	7.74	2.69	-0.94	0.56
IX	RACVM	34.96	13.97	8.53	2.77	-0.66	-0.50
X	RACVM	14.17	1.89	1.3	-7.02	-7.6	0.42
XI	RACVM	24.4	2.43	1.87	-3.43	3.78	0.44
XII	ACVM	30.1	10.9	3.42	-9.45	-7.76	
XIII	RACVM	24	904.71	13.67	-7.59	-5.14	0.13
XIV	UCVM	14.5	1.99	3.99			

When engaging in RACVM flight, the kestrel rotated somewhat more often to the right (total: 143 sec. clockwise compared to 67 sec. counter-clockwise, at |*ω*|>0.4). The values of *τ* varied considerably, from an extreme outlier at 904 seconds (95% C.I. 48-16 760) in phase XIII, to a particularly smooth and auto-correlated trajectory immediately preceding the very distinct end of the kestrel’s flight, i.e. phase XIV, with a corresponding *τ* of 1.99 sec (95% C.I. 0.9 - 4.4).

## Discussion

Integrated OU velocity models for movement have been described for several decades (our greatest debt is to Alt [15]) but have yet to be widely or routinely applied. We propose a simple taxonomy and uniform parameterization that encompasses a range of fundamental movement modes. By providing an R package (supplementary materials) for the estimation of these models, including the change point analysis, our aim is to lower the barrier for the application of continuous time movement models by movement ecologists.

We place particular emphasis on the biological interpretability of the CVM parameters in terms of speeds and characteristic time scales. For unbiased movements, the combination of speed and autocorrelation time scale gives perhaps the most succinct measure of the tortuosity, the quantification of which has defied consensus in the literature [42]. These parameters have the further advantage that their definitions are independent of the sampling scale or the regularity of the sampling, unlike CRW parameters [2], and that they link large-scale dispersal and short-term ballistic motion in a consistent and well-defined way not possible with diffusion models [9]. Furthermore, these parameters are sufficient to predict important ecological processes like encounter rates [9, 16].

### Utility of estimation methods

The most accurate and generally applicable methods for parameter estimation are those based on likelihoods. However, the phenomenological methods (VAF fitting and CRW matching) provide useful insights. The VAF method highlights the importance of exploring the autocorrelation structure of velocities (Fig. 1 [2, 6]), a powerful diagnostic tool for high-frequency movement data. Examples of data for which a VAF based analysis is most relevant includes videography [14, 15, 32], hydroacoustic telemetry of fishes, or biologged movements of birds, where data are available at intervals on the order of seconds. For most remote telemetry of large animals, sampling resolutions are substantially lower and at least somewhat irregular. In those cases, the empirical VAF will generally be too noisy and difficult to estimate to provide useful insights.

The CRW matching approach underlines the fact that the UCVM model is a continuous time analogue of the CRW and conversion between the two is straightforward. Although CRW matching is applicable for regular data sampled at frequencies more or less on the order of the characteristic time scale of movement, it can be quite a useful tool for exploring and simulating movement processes. For example, Laidre et al. [43] used observed CRW parameters for polar bear movements, sampled at 4-day intervals (i.e. regular and weakly correlated), to generate a range of UCVM tracks for a simulation-based analysis of encounter rates. Furthermore, the conversions are useful for interpreting reported CRW parameter estimates in terms of approximate speeds and time scales of movement.

The likelihood maximization tools are, in principle and practice, the superior methods as they provide the most natural framework for model comparison and selection are are robust to irregular sampling. The position likelihood fully exploits the dependency between all locations directly, without the extra step of estimating velocities. For sparsely sampled data, this is the only way to obtain estimates of the movement speed for a tortuous path, itself a useful application. Similar dense likelihood matrices have been applied to fit movement models where correlated velocities are coupled with spatial centers of attraction [13] and to model Brownian movements with errors [44]. The CVM models are spatially non-stationary, requiring estimation to be conditioned on the initial state (location and estimated velocity) which are of little intrinsic biological interest. Recent innovations allow for the estimation of the speed and time scale parameters without this conditioning by taking the OUF model [a mixed spatial and velocity OU model, see [13]] and letting the spatial time scale of relaxation go to infinity [17, 45].

The main drawback of the position likelihood method is the computational cost of maximizing over a dense matrix [13]. The problem is largely mitigated by the Kálmán filter developed by Johnson et al. [12]. However, for many applications the velocity likelihood is a useful compromise between accuracy, speed, and simplicity. The change point analysis we performed on the bowhead whale illustrates how the relative strengths of the two likelihood methods can be leveraged: the sweeping search of the change points requires that estimates be obtained thousands of times over a single time series. This is doable within minutes on a typical laptop computer. But because the mean intervals in the bowhead whale data were greater than the estimated time scales, the position likelihood was necessary in the second step: to obtain accurate estimates within the identified phases.

The fact that sparse sub-samplings of the bowhead whale data yielded consistent estimates is a testament to the robustness of the likelihood method. In fact, an irregular sampling of observations can provide more precise estimates than a regular sampling. This possibly counterintuitive result is explained simply by the fact that points that are closer in time providing more information about the autocorrelation structure than points that are more separated.

Although we placed some emphasis throughout this study on accuracy of parameter estimates, when applied to real data, bias in movement parameters is often of secondary importance. Heuristically, it is more important to analyze the structure, i.e. identify the moments in time or causes for changes in the parameter values. With that in mind, any of the methods, including more biased ones, should be able to accurately identify changes in properties of movement tracks.

### Interpretation and applications

The estimation of scale-independent and biologically meaningful parameters from data is increasingly important as movement research is increasingly driven by large data sets. Ever more individuals are tracked, with ever more opportunities to make comparisons across and within populations [46]. The methods developed here allow for robust, consistent estimation of movement parameters for variable data, thereby facilitating meta-analyses. It is straightforward to obtain and compare these estimates for any number of individuals across different populations or in different seasons, without major concern for differences in sampling regimes, thereby obviating the need for interpolation or subsampling.

There are dangers, however, to fitting and interpreting the CVM model blindly to data of long duration. The CVM models are Markovian in velocity, which implicitly exclude the possibility of long term memory effects or structures. Most organisms have a strong diel cycling to their behavior, alternating between periods of activity and rest or returning to dedicated nesting, bedding or denning sites, or are spatially constrained by home ranges or territories. None of these phenomena are captured in the CVM model, which has neither periodicity nor spatial constraints. The estimated parameters from a behaviorally complex track, for example the complete bowhead whale track, summarize all the behaviors (foraging, transitional, diving, non-moving), as well as the frequency of transitions between different states (a higher rate of change will lower the time scale of autocorrelation).

For these reasons, the CVMs are, much like the CRW, best to consider as null models of movement most applicable for the characterization of behaviorally homogeneous sections of movement data. They are analogous to classical summary statistics, like means and variances, the structure of which can be modeled and tested. We applied the CVM models as fundamental behavioral units in a change point analysis that provides multiple important advantages over existing tools. First, the parameters are more biologically meaningful than, e.g., the mean, standard deviation and autocorrelation of the persistence and turning velocities recommended in [11], the Brownian diffusion parameter [22], or the step lengths of the Bayesian partitioning (*sensu* [20, 23]). Second, CVM estimates come with confidence intervals which help contrast different behavioral modes and lead to broader inference. A movement counterpart to a classic *t*-test emerges directly from these statistics. Third, the CVM family of models facilitates identification of fundamentally different modes of behaviors, including the directed swimming of the whale and the looping flights of the kestrel, whereas most previous methods assume a single fundamental movement model. Finally, the resulting estimates, sets of selected models, and confidence intervals, allow for the simulation of realistic movements which can be useful, for example, to simulate null distributions of animal dispersal in space and time.

The kestrel analysis exemplifies a deep, model-based exploratory analysis. The kestrel’s flight was highly structured, with multiple shifts not only in parameter values but also in the fundamental movement model. This structure appeared to be well-captured by the (R/A)CVM set of models. In conjunction with biologging devices, such as tri-axial accelerometers, the results of the partitioning can be used to separate the signatures of different flight behaviors (flapping, soaring, gliding), which can then be used to model the bioenergetics of bird movement, even from 2D trajectories [47, 48]. Such fine-scaled analysis can inform population-level understanding. For example, it has been shown that some young birds (e.g. vultures) are less efficient at thermal soaring than adults [49], while individual level differences (in, e.g., storks) in flight abilities can impact population level processes by contributing to higher mortality during migration [50].

It bears noting that it is difficult to quantify the accuracy, sensitivity and robustness of the change point analysis without a simulation or resampling-based analysis, as in Additional file 1: Appendix G. A deeper exploration of the theoretical properties of the change point analyses is a potentially interesting problem to be pursued by applied statisticians.

### Future developments

Viewing the CVM models as a family of fundamental behavioral modes allows for a wide array of potential extensions. The output of change point analysis can be combined with a clustering tool that pools movements with similar parameters, informing an objective classification of the number of fundamental movement states. The basic model can be extended, such that its parameters are functions of covariates that may relate either to the external environment (e.g. meteorological conditions, intraspecific interactions) or internal states (age, sex, etc.) of the individual. Alternatively, fundamental movements and parameters might switch between values corresponding to specific states via a hidden Markov model. Models can be extended hierarchically to explore the structure of variation among individuals and among populations.

While the overwhelming majority of animal movement data is two-dimensional, both the whale and kestrel do move in three dimensions, an aspect which we entirely ignored (or lacked data on). As a rule, vertical movements have rather different characteristics than movements along the earth’s surface: they are much shallower and constrained (e.g. to the surface for an air-breathing marine organisms, or for a bird that roosts and rests). In both of our examples the two-dimensional analysis did, in fact, yield indirect insights into third-dimensional behavior: the loopy thermal soaring of the bird occurs at higher altitudes, and the more intensive feeding phase of the whale is punctuated by a higher frequency of diving bouts. That said, extending the basic correlated velocity model to three dimensions should in principle be straightforward, as many results presented here are valid in any spatial dimension. For a narrow application to vertical helical movements, see [14].

In contrast to terrestrial animals, the whale and the kestrel are moving through media that are themselves moving, with ocean and air currents both important components of the overall movement. Under certain conditions the bulk of the advective component might be externally determined. Given independent information on those currents, it would be straightforward to subtract away those vectors and thereby isolate the component that is determined by the animal itself.

One remaining challenge (that is generally under-addressed in movement analysis) is the development of diagnostic tools to assess the validity of CVM models. For highly irregular data and a continuous stochastic model, it is difficult to summarize or visualize the distributions or to separate a deterministic term from a residual term. One approach might be to simulate tracks using the estimated parameters at the observed sampling regime and compare the distribution of some derived quantities, like per-step or total displacements.

An additional forward-looking challenge in continuous-time movement modeling is to provide an alternative for the discrete step-selection function (SSF) framework [27, 51], a powerful and widely implemented approach for quantifying animal movement responses to environmental covariates. As their name implies, step-selection functions rely on a discrete and regularly sampled unit of movement. Developing an analogous tool using a continuous time movement model as a fundamental, scale-independent, unit would be a significant advance.

## Conclusion

We review and unify a family of continuous time correlated velocity movement (CVM) models that allow for combinations of random, advective, and rotational movement with consistent and biologically meaningful parameterizations like time scales and speeds. We discuss the importance of movement velocity autocovariance functions and fundamental links to commonly used correlated random walk (CRW) models and argue that they are particularly useful for fitting movement data that are highly resolved and/or irregularly sampled. As one useful example, fitting an unbiased CVM to a bowhead whale swimming track provided a minimally biased estimate of actual mean surface speeds, even at low sub-samplings of the already irregular data. We argue that CVM’s are especially suitable as fundamental, behaviorally homogeneous units, and present an heuristic approach to applying them in a likelihood-based behavioral change point analysis. The resulting model fit yields detailed descriptions of complex trajectories in terms of biologically meaningful parameters with accompanying confidence intervals, thereby improving greatly on currently existing tools. When applied to data, the analysis identified advective from random foraging behavior in the bowhead whale track and exact moments when a lesser kestrel switched from loopy, advective thermal soaring to directed flights. In the supplementary materials, we describe the mathematical and statistical details of these models and their estimation, and - importantly - provide an R package (smoove) for the implementation of all the presented tools. We hope this paper serves to make these models more comprehensible and accessible to movement ecologists, who are constantly striving to make sense of highly structured and often inconsistently collected data, even as sample sizes, resolution and precision have increased with improving technology.

## Endnotes

^1^ The quantity **w**_*t*_ - the integral of continuous independent random fluctuations - is commonly referred to as a *Wiener process* or *Brownian motion*, while its derivate **w**_*t*_/*d* *t* is *white noise* [31].

## Additional files

Additional file 1 Appendix to: Correlated velocity models as a fundamental unit of animal movement: synthesis and applications. (PDF 621 kb)

Additional file 2 Vignette document (pdf) describing the functions and tools in the smoove, including replication of many of the results in the manuscript. (PDF 2375.68 kb)

Additional file 3 The source bundle for the smoove package. (GZ 7034.88 kb)

## Abbreviations

ACVM: advective CVM
BCPA: behavioral change point analysis
CRW: correlated random walk
CVM: correlated velocity movement
EVAF: empirical velocity autocovariance function
OU: Ornstein-Uhlenbeck process
RACVM: rotational-advective CVM
RCVM: rotational CVM
UCVM: unbiased CVM
VAF: velocity covariance function
