# Supplementary material for: Correlated velocity models as a fundamental unit of animal movement: synthesis and applications
Source: Mov Ecol. 2017 May 10;5:13. doi: 10.1186/s40462-017-0103-3 (PMC5424322; doi:10.1186/s40462-017-0103-3)
Supplement: Supplementary file 1 — Appendix to: Correlated velocity models as a fundamental unit of animal movement: synthesis and applications. (PDF 621 kb) [file 40462_2017_103_MOESM1_ESM.pdf]

Appendix to: *Correlated velocity models as a fundamental unit of animal movement: synthesis and applications*

EG, CF, WF, KL, JH-P, OO

March 17, 2017

A Definitions

All of the CVM models presented in the main text are special cases of the velocity stochastic differential equation:

$$d\mathbf{v} \quad = \quad \alpha(\boldsymbol{\mu}(t) - \mathbf{v})\, dt + \beta\, d\mathbf{w}_t \tag{A1}$$

In the definitions and derivations below, bold-facing represents variables in $d \geq 2$ dimensions, bra-ket notation and lower cased symbols to represent moments of true processes (e.g. $\langle \mathbf{v}(t) \rangle$) and overlines and capital letters to represent means of sampled data (e.g. $\overline{\mathbf{V}}(T)$).

A.1 Unbiased correlated velocity model (UCVM)

The UCVM (figure 1A) has no mean velocity component ($\boldsymbol{\mu}(t) = 0$) and a real, non-negative relaxation parameter α. Defined as $\alpha = \frac{1}{\tau}$, τ represents a characteristic time scale of auto-correlation [1, 2]. The parameter β has units of velocity $\times$ time$^{-1/2}$ which makes it difficult to interpret biologically. However, the mean squared speed of the UCVM is given by $\eta^2 \equiv \lim_{t \to \infty} \langle |\mathbf{v}(t)|^2 \rangle = \beta^2 \tau$, such that it can be written

$$d\mathbf{v} = -\frac{1}{\tau} \mathbf{v}\, dt + \frac{\eta}{\sqrt{\tau}}\, d\mathbf{w}_t. \tag{A2}$$

The velocity is perturbed by the two-dimensional white noise $d\mathbf{w}_t$, with a tendency to relax towards zero at a characteristic time scale τ. The η parameter can be thought of as the random component of the root mean squared speed. The actual mean speed of the UCVM is given by $\nu = \frac{\sqrt{\pi}}{2} \eta$ [3, Appendix B]. Thus, the unbiased continuous time movement can also be expressed $\mathbf{z} \sim \text{UCVM}(\tau, \nu)$, which is conveniently explicit in terms of a characteristic time scale and a mean speed of movement. Formally, the process is also conditioned on the initial velocity $\mathbf{v}_0$, but that is a parameter of little interest or impact on the process when $(t - t_0) \gg \tau$. As $\tau \to 0$, the UCVM approaches Brownian motion, whereas as $\tau \to \infty$, the UCVM approximates linear motion.

A.2 Advective CVM (ACVM):

The ACVM (figure 1B) includes a constant non-zero mean velocity $\boldsymbol{\mu}$

$$d\mathbf{v} = -\frac{1}{\tau}(\boldsymbol{\mu} - \mathbf{v})\, dt + \frac{\eta}{\sqrt{\tau}}\, d\mathbf{w}_t. \tag{A3}$$

This model has mean squared speed $\langle |\mathbf{v}|^2 \rangle = \sqrt{\eta^2 + |\boldsymbol{\mu}|^2}$, and we can thus write the model in terms of advective and random components of velocity: ACVM$(\tau, \eta, \boldsymbol{\mu})$.

To solve for the mean speed $\langle |\mathbf{v}| \rangle$ of the ACVM, we note that x and y components of velocity are independent Gaussian normal variables with mean and variance given by

$$\langle v_k \rangle = \mu_k \tag{A4}$$

$$\text{var}(v_k) = \frac{\eta^2}{2} \tag{A5}$$

where k is the index of dimensionality, and μ_k represents the x or y component of the mean velocity $\boldsymbol{\mu}$ [4]. The mean speed of the process

$$\langle |\mathbf{v}| \rangle = \sqrt{v_x^2 + v_y^2}$$

has a non-central bivariate Chi distribution, also known as a Rice distribution [5], the mean of which is given by

$$\langle |\mathbf{v}| \rangle = \frac{\nu\sqrt{\pi}}{2} e^{x/2}((1-x)I_0(-x/2) - xI_1(-x/2)) \tag{A6}$$

where $x = |\boldsymbol{\mu}|^2/\nu^2$ and I_0 and I_1 are modified Bessel functions of the first kind, of order 0 and 1 respectively.

A.3 Rotational CVM (RCVM)

If the α parameter in equation 1 is a 2×2 matrix of the form $\begin{bmatrix} 1/\tau & -\omega \\ \omega & 1/\tau \end{bmatrix}$, the general model in 2 dimensions becomes:

$$dv_x = \left(\frac{1}{\tau}(\mu_x - v_x) - \omega(\mu_y - v_y) \right) dt + \frac{\eta}{\sqrt{\tau}} dw_{x,t}$$

$$dv_y = \left(\frac{1}{\tau}(\mu_y - v_y) + \omega(\mu_x - v_x) \right) dt + \frac{\eta}{\sqrt{\tau}} dw_{y,t}$$

which introduces a rotational component with angular velocity ω (figure 1C). Following [6], this model is more compactly expressed in complex number notation as

$$d\mathbf{v} = \left(\frac{1}{\tau} + i\omega \right) (\boldsymbol{\mu} - \mathbf{v})\, dt + \frac{\eta}{\sqrt{\tau}}\, d\mathbf{w}_t, \tag{A7}$$

where the real and imaginary components represent x and y coordinates of all variables and parameters. The mean squared speed of the process is not affected by the rotation and thus it is equal to $\langle |\mathbf{v}|^2 \rangle = \mu^2 + \eta^2$. A characteristic spatial scale (i.e. diameter) of rotation can be defined as $\rho = \eta/\omega$, i.e. the ratio between the random speed component and the angular speed. Advection and rotation can also be combined (figure 1D). We express rotational models with and without the advective component as RACVM$(\tau, \eta, \omega, \boldsymbol{\mu})$ and RCVM(τ, η, ω), respectively. Note that the root mean square speed of movement for an RACVM is always given by: $\sqrt{|\overline{\mathbf{v}^2}|} = \sqrt{\eta^2 + |\boldsymbol{\mu}|^2} = \sqrt{\eta^2 + \mu_x^2 + \mu_y^2}$

B Statistical properties of the CVM

The key statistical properties of a CVM process are the expectations, variances, and autocorrelation of its velocities and positions [4, 1, 7, 8].

B.1 Moments

For a trajectory $\mathbf{z}(t) \sim \text{ACVM}(\eta, \tau, \boldsymbol{\mu}|\mathbf{v}_0)$, both of the velocity components ($v_x(t)$ and $v_y(t)$) are Gaussian variables with mean and variance

$$\langle v_k(t) \rangle = \mu_k + v_{0,k} e^{-t/\tau}, \tag{A8}$$

$$\text{var}[v_k(t)] = \eta^2 \left(1 - e^{-2t/\tau}\right). \tag{A9}$$

where k is the index of dimension x and y, dropped in subsequent expressions. After a transient period, the mean velocity decays to the advective velocity μ and the variance of the velocity approaches η^2. Note, the variance of velocity is 0 at time lag 0, reflecting the basic property of "smoothness" in an autocorrelated velocity model.

The respective integrals ($z_x(t)$ and $z_y(t)$) are also independent Gaussian variables with:

$$\langle z(t) \rangle = z_0 + \mu\, t + v_0 \tau (1 - e^{-t/\tau}), \tag{A10}$$

$$\text{var}[z(t)] = 2\eta^2 \tau^2 \left(t/\tau - 2(1 - e^{-t/\tau}) + (1 - e^{-2t/\tau})/2\right) \tag{A11}$$

and the covariance between $v(t)$ and $z(t)$ is

$$\text{cov}[v(t), z(t)] = \eta^2 \tau \left(1 - 2e^{-t/\tau} + e^{-2t/\tau}\right). \tag{A12}$$

These results can be converted to expressions for mean squared speeds and mean squared displacements.

The expectation and variance of the RCVM can be computed by introducing a rotational frame of reference, i.e. making the substitution $\mathbf{v}^\star(t) = \mathbf{v}(t) \exp(i\omega t)$. In this frame of reference, the RCVM reduces to an unbiased CVM. Thus, back-transforming, the RACVM velocity has expectation:

$$\langle \mathbf{v}(t) \rangle = \mu + \mathbf{v}_0 \exp\left(\frac{t}{\tau} + i\omega t\right), \tag{A13}$$

with the same variance as above. Perhaps surprisingly, the x and y components of velocity are uncorrelated in this model as well, though the expressions for the positions and the cross-correlations are more complex.

B.2 Velocity autocovariance function

An important derived quantity of a correlated velocity process is the *velocity autocovariance function (v.a.f.)* C_v. Following [9, 10, 3]. The v.a.f. is defined as the expected dot product of a random velocity process over lags:

$$C_v(\Delta t) = \langle \mathbf{v}(t + \Delta t) \cdot \mathbf{v}(t) \rangle, \tag{A14}$$

where the product is the dot product of vectors and the expectation is taken over all possible starting times t. If we additionally constrain $(t - t_0)/\tau \gg 1$, where t_0 is the initial time of the process (i.e. the velocity process $\mathbf{v}(t)$ has had time to relax to a stationary state), or if the process is simply initiated from the stationary distribution, then the v.a.f. is a well defined function independent of t.

The velocity autocovariance of the RACVM (equation A7, [6]) is

$$C_v(\Delta t) = |\boldsymbol{\mu}|^2 + \eta^2 e^{-\frac{\Delta t}{\tau}} \cos(\omega \Delta t). \tag{A15}$$

The autocovariance function at lag 0 is equal to the mean squared speed ($|\boldsymbol{\mu}|^2 + \eta^2$). With increasing time lag, the function decays exponentially with scale τ to the mean velocity squared ($|\boldsymbol{\mu}|^2$). The decay is oscillatory with frequency ω if the movement is rotational (figure 1, right panels). The velocity autocovariance and autocorrelation functions are continuous time generalizations of the standard autocovariance and autocorrelation functions used in time-series analysis.

Note that the definition of the v.a.f. proposed by Alt [9] differs somewhat from the standard definition of covariance: $Cov(A,B) = \langle AB \rangle - \langle A \rangle \langle B \rangle$, which would subtract away the square of the mean term $|\boldsymbol{\mu}|^2$. For our purposes, this is an advantage of Alt's definition, since all the key parameters of the process are reflected in the theoretical v.a.f. of the RACVM.

C Estimating parameters from data

We present two general kinds of methods for estimating parameters of CVM processes: phenomenological methods that match some statistical properties of the observed trajectory, analogous to method of moments estimation, and likelihood-based methods. In the presentation that follows we consider observations of $n+1$ locations $\mathbf{Z}_0, \mathbf{Z}_1, ...\mathbf{Z}_n$ sampled from a CVM process at times $T_0, T_1....T_n$, with n intervals $\Delta T_i = T_i - T_{i-1}$.

For data sampled at high resolution (i.e. $\overline{\Delta T} \ll \tau$), whether regularly or irregularly, the velocity of the process can be estimated directly: $\mathbf{V}_i = (\mathbf{Z}_i - \mathbf{Z}_{i-1})/\Delta T_i$, and the relevant speed parameter (mean speed ν or random root mean square speed η) can be directly estimated by matching the appropriate moments: $\widehat{\nu} = \overline{|\mathbf{V}_i|}$ and $\widehat{\eta} = \sqrt{\overline{|\mathbf{V}_i|^2}}$.

For data that are of lower resolution, a direct velocity calculation from position data is always an underestimate since there is no consideration for the curve of the trajectory in the interval between the two observations. One way to correct for this bias is to compute a cubic spline interpolation of $\mathbf{Z}$ against T and to use the estimated velocities from the splined functions. Specifically, the sets of points $\{T_i, Z_{x,i}\}$ and $\{T_i, Z_{y,i}\}$ can be separately splined to obtain estimated location function $\widetilde{Z_x}(t)$ and $\widetilde{Z_y}(t)$, and recombined to obtain a smoothed, interpolated approximation of the trajectory $\widetilde{\mathbf{Z}}(t)$. Estimated velocities at times T_i can be computed at arbitrarily high resolution from the splined track and used for estimation. This somewhat *ad hoc* correction can improve the estimates depending on the relationship of the sampling intervals ΔT to τ and the validity of the spline approximation. A reasonably estimated velocity provides the basis for two of the methods desribed below (methods I and III below).

C.1 Method I: Matching to CRW parameters

For observations that are too coarse for a direct estimation of velocities - but regularly sampled - the parameters of the UCVM can be matched to the parameters of the unbiased correlated random walk (CRW). A CRW with steps of independent lengths L_i, independent turning angles θ_i and constant sampling interval ΔT has characteristic time scale

$$\tau = \begin{cases} \left(1 - \frac{1}{e}\right)\left(\frac{\lambda}{\lambda - \kappa}\right) T & \text{for } \kappa \leq \frac{\lambda}{e} \\ \frac{\log(\lambda) - 1}{\log(\kappa)} T & \text{for } \kappa > \frac{\lambda}{e} \end{cases}$$

where $\lambda = \left\langle L^2 \right\rangle / \left\langle L \right\rangle^2$ is a parameter of step length variability and the coefficient $\kappa \in [-1, 1]$ is a clustering coefficient equal to $\langle \cos(\theta) \rangle$ and exponential weighting for the residual variance structure. An estimate of the velocity parameter is

$$\widehat{\nu} = \sqrt{\frac{\pi}{8}\left(\langle L^2 \rangle + \langle L \rangle^2 \frac{2\kappa}{1-\kappa}\right)\frac{1}{\tau \Delta T}}.$$

Approximate 95% confidence intervals can be obtained for these estimates by calculating variances of the estimates of $\widehat{\kappa}$ and $\widehat{\lambda}$ and resampling from these distributions.

We derive C.I.'s for τ and ν from the C.I.'s for the CRW parameters $\widehat{\kappa}$ and $\widehat{\lambda}$. Assuming that the turning angles are drawn from a wrapped Cauchy distribution with zero mean, such that

$$f(\theta|\kappa) = \frac{1 - \kappa^2}{2\pi(1 - \kappa^2 - 2\kappa \cos(\theta))}$$

the likelihood of the parameter is given by

$$\mathcal{L}(\kappa|\theta) = \prod_{i=1}^{n} f(\kappa|\theta_i)$$

We use this likelihood to numerically obtain a maximum likelihood estimate $\widehat{\kappa}$ and obtain the variance around $\widehat{\kappa}$ from the Fisher information.

Recalling that $\lambda = \left\langle S^2 \right\rangle / \left\langle S \right\rangle^2$, the estimator is $\widehat{\lambda} = \frac{s^2+\overline{x}^2}{\overline{x}^2}$, where s^2 and $\overline{x}$ are the sample variance and mean of the step lengths, respectively. The variance of this estimator can be approximated via Taylor expansion as:

$$\begin{aligned} \mathrm{var}(\widehat{\lambda}) &= \mathrm{var}\left(s^2/\overline{x}^2\right) \\ &\approx \frac{\left\langle s^2 \right\rangle^2}{\left\langle \overline{x}^2 \right\rangle^2} \frac{\mathrm{var}(s^2)}{\left\langle s^2 \right\rangle^2} + \frac{\mathrm{var}(\overline{x}^2)}{\left\langle \overline{x}^2 \right\rangle^2} \\ &\approx \frac{\sigma^4}{\mu^4}\left(\frac{\Sigma_4}{\sigma^4} + \frac{\Xi_4}{\mu^4}\right). \end{aligned} \quad \text{(A16)}$$

where σ and μ are the true mean and variance, and Σ_4 and Ξ_4 are fourth moments of s and $\overline{x}$, respectively, given by

$$\Sigma_4 = \frac{2(n-1)\sigma^4}{n^2}$$
$$\Xi_4 = \frac{4\mu^2\sigma^2}{n}.$$

Plugging these results into (A16), and substituting in the estimates s and $\overline{x}$ for σ and μ, gives

$$\mathrm{var}(\widehat{\lambda}) \approx \frac{s^2}{\overline{x}^4}\left(\frac{2(n+1)}{n^2} + \frac{4s^2}{n\overline{x}^2}\right).$$

According to the assumptions of the CRW, the turning angles and step lengths are independent, and therefore the estimates for κ and λ are independent. In order to approximate confidence intervals for τ and ν, we sample from normal distributions with given means and variances of κ and λ and compute the resulting estimates of τ and ν. This algorithm is implemented in the `smoove` package included as an on-line supplement.

These estimates are only valid if the data conform with the assumptions of the CRW, the most important of which is independence between and within the step lengths and turning angles. We anticipate this approximation provides better results for relatively coarse subsamplings, i.e. where ΔT is on the order of τ and for cases where the total time interval is much greater than the characteristic time scale ($T_n - T_0 \gg \tau$).

C.2 Method II: Fitting the velocity auto-covariance

For regularly sampled data collected at very high resolution (e.g. video data), an analysis of the velocity auto-covariance function illuminates the structure of the movement, i.e. the time scale, advection and rotation. Given locations $\mathbf{Z}_0, \mathbf{Z}_1, ..., \mathbf{Z}_n$ sampled at a regular interval ΔT the auto-covariance function can be estimated from the empirical velocity autocovariance function (EVAF) given by

$$\widehat{C_v}(j) = \frac{1}{n-j}\sum_{i=1}^{n-j} \mathbf{V}_i \cdot \mathbf{V}_{i+j}. \quad \text{(A17)}$$

For CVM tracks with and without advection and rotation, the theoretically predicted VAF (equation A7) and the EVAF's for simulated tracks are illustrated in figure 1. The theoretical curves can be fitted to the EVAF to estimate parameters in a variety of ways, as described in [9] and applied the context of helical movement in [2].

For example, in the case of an UCVM(τ, ν), the time scale τ can be estimated by fitting the theoretical prediction of the velocity auto-covariance, e.g. with a linear fit of the model:

$$\log\left(\frac{\pi\widehat{C_v}(\Delta t)}{4\widehat{\nu}^2}\right) = \frac{1}{\widehat{\tau}}\Delta t + \epsilon,$$

where ϵ is the residual term.

Because the vector of speeds of $|\mathbf{V}|$ is itself autocorrelated, confidence intervals around $\widehat{\nu}$ are obtained by adjusting the standard error as: $se_\nu = \frac{s_\nu}{\sqrt{n}(1-\widehat{\phi})}$, where s_ν is the sample standard deviation of $\widehat{\nu}$ and $\widehat{\phi}$ is the estimate of the first order autocorrelation of $|\mathbf{V}|$.
Because $\log(C_v)$ is itself highly correlated, we account for that correlation by using a generalized least squares fit with a first order autocorrelation and exponential weighting for the residual variance structure [11], obtaining the adjusted confidence intervals around the slope, and back-transforming to obtain 95% confidence intervals around $\widehat{\tau}$. The generalized least square fitting was performed using the `gls` function in the `nlme` package in R [12].

C.3 Method III: Velocity likelihood

In terms of the observed velocity of the process $\mathbf{V}$, the likelihood for the unknown parameters θ can be expressed conditioned on the previous observation of the velocity:

$$L(\theta|\mathbf{V}, T) = \prod_{i=1}^{n} f(\mathbf{V}_i|\mathbf{V}_{i-1}, \Delta T_i, \theta). \tag{A18}$$

In the UCVM and ACVM, the x and y components of the CVM velocity are independent Gaussian processes, the distribution function $f(\mathbf{v}_{i+1}) = \phi(\boldsymbol{\mu}_i, \Sigma)$ where ϕ is the normal distribution function with mean vector $\boldsymbol{\mu}_i = \{\mu_{x,i}, \mu_{y,i}\}$ and Σ is a diagonal matrix with variances $\{\sigma^2_{x,i}, \sigma^2_{y,i}\}$, where mean and variance components obtained directly from the distributions summarized in the section above. For example, for UCVM(τ, ν):

$$\begin{aligned} \mu_i &= v_{i-1}\, e^{-\Delta T_i/\tau} \\ \sigma_i^2 &= \frac{2\nu^2}{\pi}\left(1 - e^{-2\Delta T_i/\tau}\right) \end{aligned}$$

with dimensional indices implicit. This likelihood function is smooth and can be numerically maximized to estimate τ. Confidence intervals can be approximated from the Hessian of the log-likelihood. We numerically estimated the parameters, seeding the initial values obtained from the method of moments estimators. For example, for the ACVM we seed the likelihood with $\tilde{\boldsymbol{\mu}} = |\mathbf{V}|$ and $\tilde{\eta} = \sqrt{|\mathbf{V} - \tilde{\boldsymbol{\mu}}|^2}$.

Method IV: Position likelihood

The statistical properties of the OU process can be used to obtain a likelihood of the parameters τ, ν and initial velocity $\mathbf{v}_0$ based directly on the raw location data $\mathbf{Z}$. Complete derivations are provided in appendix E. In one dimension the expressions for the MLE's of the ν and $\mathbf{v}_0$ parameters given position data Z_k at times T is:

$$\widehat{v_0}(\tau) = \frac{Z_k^T\, \mathbf{S}^{-1} M}{M^T\, \mathbf{S}^{-1} M} \tag{A19}$$

$$\widehat{\nu^2}(\tau) = \frac{1}{n}(Z - \widehat{v_0}M)^{\mathrm{T}}\, \mathbf{S}^{-1}(Z_k - \widehat{v_0}M) \tag{A20}$$

where M is a vector of length n with elements $M_i = \tau\,(1 - \exp(T_i/\tau))$, $\mathbf{S}(\tau^2, T)$ is a symmetric $n \times n$ matrix given by

$$S_{ij} = \tau^2 \left(\frac{T_i}{\tau} + (1 - \epsilon_{ij}) \left(\frac{1 + \epsilon_{ij}}{2} + (1 - \kappa_{ij})(1 - \epsilon_{ij}) - 2 \right) \right),$$

and $\epsilon_{ij} = \exp(-\min(T_i, T_j)/\tau)$ and $\kappa_{ij} = \exp(-|T_j - T_i|/\tau)$.

Because both of these parameters depend only on $\widehat{\tau}$, a numerical MLE need only be obtained for the time scale τ. Standard deviations of the MLE's are analytically derived from the second derivative of the log-likelihoods at $\widehat{v_0}$ and $\widehat{\nu^2}$:

$$\widehat{\sigma_{v_0}} = \sqrt{\frac{\widehat{\nu^2}}{M^T\,\mathbf{S}^{-1} M}} \tag{A21}$$

$$\widehat{\sigma_{\nu^2}} = \sqrt{\frac{2}{n}}\,\widehat{\nu^2}. \tag{A22}$$

Note that the estimate of ν becomes more precise ($\sigma_{\nu^2} \to 0$) at greater sample sizes ($n \to \infty$), whereas the precision of v_0, which is only meaningfully informed only by the first several data points, depends on the overall mean velocity estimate. We estimated an approximate confidence interval for τ by numerically computing the Hessian at $\widehat{\tau}$.

Even though only one parameter is computed numerically, maximizing the likelihood requires the iterated inversion of a fairly dense $n \times n$ matrix which can therefore be prohibitively slow. A computationally more efficient method to obtain the maximum likelihood estimates is with the aid of a Kalman filter, a method has been developed and described in detail by [1] and compiled in the `crawl` package in R [13]. We refer the reader to the original article and the associated appendices, noting that the parameters those authors refer to as β and σ correspond in our parameterization to $1/\tau$ and η^2/τ, respectively. We note further that the Johnson et al. [1] implementation is also capable of estimating biased movements, though not rotation.

D Simulation study

We conducted a comprehensive simulation experiment to assess the performance of the four parameter estimation methods at various sampling resolutions and time series durations for estimating the UCVM(ν, τ). In this context "resolution" refers to the sampling rate relative to the characteristic time scale, i.e. $\Delta T \ll \tau$ is high and $\Delta T \gg \tau$ is coarse. Similarly, the duration of the trajectory is relative to the characteristic time scale: a long trajectory is one where T_{max} is orders of magnitude greater than τ. We simulated 100 UCVM trajectories at high resolution ($\Delta T = 0.01$) for each of three different parameter values for τ (1/4, 1, 4) up to $T_{max} = 100$ (i.e. $n = 10\,000$). We estimated the parameters for the complete dataset using the VAF fit and velocity likelihood method (the CRW matching method is not applicable at high resolutions and the position likelihood method is prohibitively slow). Next, we subsampled the trajectories regularly at $\Delta T = 1$ ($n = 100$) and estimated the parameters using all four methods. At $\tau = 1/4$ a track can be considered low resolution but long duration, while at $\tau = 4$ a tracks can be considered high resolution but short duration. Finally, we randomly sampled 100 observations from the full trajectories consisting of 10000 observations, and estimated the parameters using the two likelihood methods, to test their robustness to irregular sampling.

For each method and parameter value set, we obtained the 95% central range of the estimates and defined a method as "significantly biased" if the true value lay outside of that range. We quantified the precision of an estimate by computing the width of the inter-quantile range (IQR) of the ν estimates and the width of the IQR of the log of the τ estimates. We also report the correlation between estimates of ν and $\log(\tau)$.

We benchmarked the computational performance for a range of tracks with sample sizes ranging from $n = 50$ to $n = 400$, timing the estimation for 50 separately simulated tracks at each sample size. The benchmarking was performed on a 2.6 GHz, 4.0 GB RAM notebook computer.

D.1 Results

The main results of the comparison between four methods for estimating the UCVM are presented in figure A1 and in the supplementary materials: confidence intervals for all methods in appendix F.2, and estimates of the x and y components of $\mathbf{v}_0$ for the position likelihood in appendix F.3.

As predicted, the VAF fit (I) and velocity likelihood (III) provided accurate estimates of both parameters at high resolutions. The velocity likelihood method was more precise for τ than the VAF fit (IQR of 100 estimates at $\tau = 1/4 : 0.08$ and 0.12, $\tau = 1 : 0.11$ and 0.26, $\tau = 4 : 0.31$ and 0.52 for the two methods, respectively) while both performed about equally well for the ν estimate. The velocity spline considerably improved the estimates, especially for speed, for both of these methods. Note the extremely high positive correlation (between 0.98 and 1.0) between $\widehat{\tau}$ and $\widehat{\nu}$ in the estimates for the velocity likelihood (figure A1).

At lower resolutions ($\tau = 1$), the VAF method provided the least precise estimates for τ (IQR between 0.39 and 0.79, compared to 0.07-0.31 for all other methods and all resolutions). At the coarsest resolution ($\tau = 4$), the VAF method was incapable of estimating confidence intervals since the drop to near zero correlation occurred within a single lag step (appendix table A1).

The CRW matching (II) provided extremely biased estimates of τ, overestimating low values and underestimating high values; the 95% range only came close to covering the true value at $\tau = 1$. However, the CRW method performed about as well as other methods at estimating ν. In fact, the precision of the speed estimates across all methods were comparable.

The position likelihood (IV) provided the most consistently unbiased estimates (figure A1D and appendix F.3) across all resolutions whether computed directly or via the Kalman filter. The 95% confidence intervals (appendix F.2) for τ appeared to provide mostly accurate inference, though the intervals for the speed ν were too narrow at low values of τ and somewhat too wide for larger values of τ.

Benchmarking results for all four methods, with the additional comparison of the position likelihood against the Kalman filter estimation, are presented in appendix F.1. For datasets on the order of a few hundred observations, the computation times even for the slowest method were on the order of seconds or faster. The CRW matching method, which is a direct computation requiring no numerical optimization, was fastest. The position likelihood was, by far, slowest, as the maximization of a likelihood requiring the inversion of dense $n \times n$ matrix scales as $\mathrm{O}(n^3)$. The Kalman filter of Johnson [1], in contrast, scales as $O(n)$, an enormous improvement (already at $n = 400$, the Kalman filter is 10 times faster). The velocity likelihood method also scaled as $O(n)$, stably about an order of magnitude faster than the Kalman filter across the range of sample sizes.

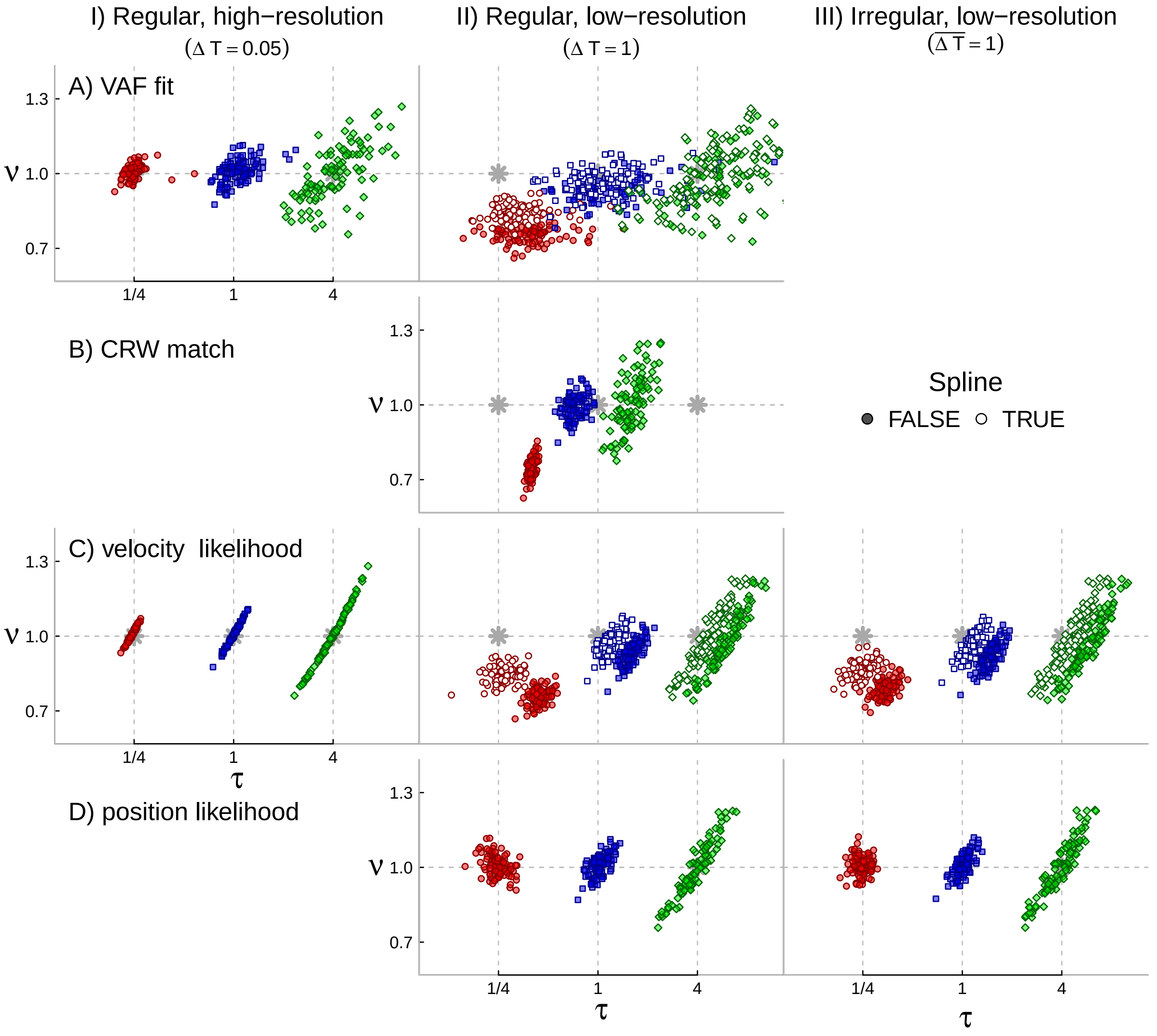

Figure A1: Results of estimation of UCVM parameters using the four methods (A-D: VAF fit, CRW match, the velocity and position likelihood methods) at different sampling resolutions and regularity (I. Regular $\Delta T = 0.05$, II. Regular $\Delta T = 1$, III. Irregular $\overline{\Delta T} = 1$). In all simulations, $T_{max} = 200$ and the true speed $\nu = 1$. Red, blue and green points respectively represent true values of $\tau = 1/4, 1, 4$ used for the simulations, reflecting a range from long - coarse resolution to short - high resolution sampling. The x-axis represents the values for the time-scale τ, the y-axis represents values for the speed ν. Open circles represent estimates using the cubic spline correction. Note that the x axis is on a log scale. At high resolution, the CRW estimates were so far off most fell off the range of the plots, whereas the position likelihood would take a prohibitively long time to compute. The VAF and CRW methods rely on regularly sampled observations. Therefore, these method/sampling combinations are omitted from the figure.

E Deriving the position Likelihood for CVM process

The likelihood in one of the dimensions observed at times T (for clarity we use Z for Z_x and v_0 is the x-component of $\mathbf{v}_0$, noting that all the equations in the y dimension are identical) is

$$\mathcal{L}(v_0, \nu, \tau | Z, T) = \phi\left(Z,\, \mu = v_0 \mathbf{m}(\tau, T),\, \Sigma = \nu^2 S(\tau^2, T)\right) \tag{A23}$$

where $\phi()$ represents the multivariate Gaussian density function with mean μ and variance Σ, and $\mathbf{m}(\tau, T)$ is a $n \times 1$ vector and $S(\tau^2, T)$ is a symmetric $n \times n$ matrix given by

$$\begin{aligned} m_i &= \tau(1 - e^{t_i/\tau}) \\ S_{ij} &= \tau^2 \left(\frac{t_i}{\tau} + (1 - \epsilon_{ij}) \left(\frac{1 + \epsilon_{ij}}{2} + (1 - \kappa_{i,j})(1 - \epsilon_{ij}) - 2 \right) \right) \end{aligned}$$

where $\epsilon_{ij} = \exp(-\min(t_i, t_j)/\tau)$ and $\kappa_{ij} = \exp(-|t_j - t_i|/\tau)$.
The log-likehood is:

$$\ell = -\frac{1}{2} \log(\nu^{2n} |S|) - \frac{1}{2\nu^2} (\mathbf{z} - v_0 \mathbf{m})^{\mathrm{T}} S^{-1} (\mathbf{z} - v_0 \mathbf{m}) - \frac{n}{2} \log(2\pi)$$

Taking the derivatives with respect to v_0 and ν and setting equal to zero gives the following expressions for the MLE's:

$$\widehat{v_0}(\tau) = \frac{\mathbf{z}^T S^{-1} \mathbf{m}}{\mathbf{m}^T S^{-1} \mathbf{m}} \tag{A24}$$

$$\widehat{\nu^2}(\tau) = \frac{1}{n} (\mathbf{z} - \widehat{v_0} \mathbf{m})^{\mathrm{T}} S^{-1} (\mathbf{z} - \widehat{v_0} \mathbf{m}) \tag{A25}$$

Because both of these parameters depend only on $\widehat{\tau}$, a numerical MLE need only be obtained for the single variable τ. Standard deviations of the MLE's can be computed obtained directly from the second derivatives of the log-likelihoods for v_0 and ν^2:

$$\sigma_{v_0} = \sqrt{\frac{\widehat{\nu^2}}{\mathbf{m}^T S^{-1} \mathbf{m}}} \tag{A26}$$

$$\sigma_{\nu^2} = \sqrt{\frac{2}{n}} \widehat{\nu^2} \tag{A27}$$

Note that the estimate of ν becomes more precise with greater sample sizes, whereas the precision of v_0 - which is only meaningfully informed only by the first several data points - depends on the overall mean velocity estimate. An approximate confidence interval for τ is estimated by numerically computing the second derivative of the Hessian at $\widehat{\tau}$.

F UCVM estimation

F.1 Benchmarking

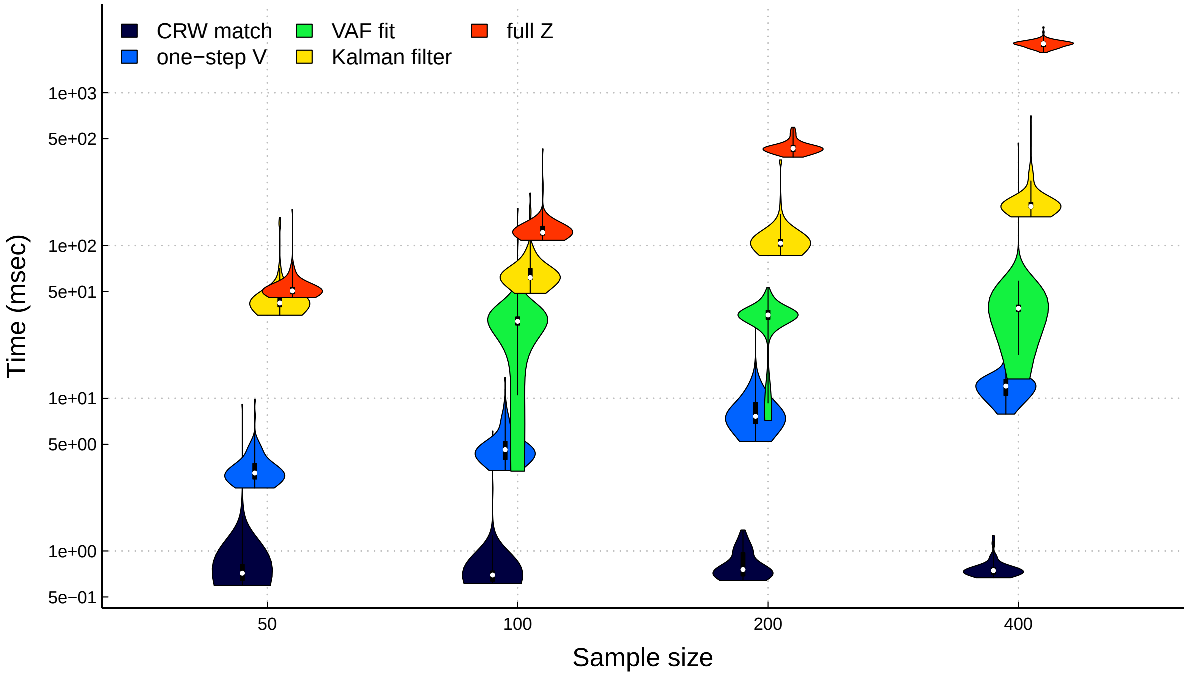

Figure A2: Violinplots of estimation times for the methods against various sample sizes ranging from 50 to 400 locations. The estimates were obtained for 100 tracks at each sample size. It is nearly impossible to obtain a fittable velocity autocorrelation function for the time series at only $n = 50$, therefore these fits were omitted from the benchmarking.

F.2 Confidence intervals

Table A1: Assessing the accuracy of the inference of all estimation procedures: N refers to the number of times an estimate was obtained. P1 refers to the proportion of times the true value of the estimates fell within the 95% CI and P2 is a "bias-corrected" proportion, referring to the proportion of times the mean of the estimates fell within the 95% CI.

		time-scale τ			speed ν	
Method	N	value	P1	P2	P1	P2
**High resolution, regular sampling**						
VAF fit	100	0.25	0.25	0.26	0.95	0.95
	100	1	0.05	0.11	1	1
	100	4	0.04	0.03	1	1
velocity likelihood	100	0.25	0.91	0.90	0.91	0.92
	100	1	0.99	0.99	0.99	0.97
	100	4	0.90	0.89	0.88	0.88
**Low resolution, regular sampling**						
VAF fit	1	0.25	0.00	1	0.00	1
	33	1	0.03	0.24	0.67	0.70
	96	4	0.27	0.40	0.88	0.88
(with spline)	0	0.25				
	28	1	0.07	0.21	0.64	0.64
	96	4	0.22	0.28	0.84	0.84
CRW match	100	0.25	0.00	1	0.00	0.99
	100	1	0.96	1	1	1
	100	4	0.00	1	1	1
velocity likelihood	100	0.25	0.01	0.99	0.00	0.94
	100	1	0.03	0.99	0.81	0.98
	100	4	0.70	0.91	0.88	0.91
(with spline)	97	0.25	0.89	0.98	0.00	0.91
	100	1	0.78	0.99	0.91	0.97
	100	4	0.94	0.90	0.87	0.88
position likelihood	100	0.25	0.97	0.97	1	1
	100	1	0.98	0.98	0.99	0.99
	100	4	0.89	0.88	0.79	0.78
**Low resolution, irregular sampling**						
velocity likelihood	100	0.25	0.33	0.97	0.00	0.94
	100	1	0.17	0.98	0.74	0.98
	100	4	0.72	0.93	0.88	0.91
(with spline)	100	0.25	0.96	0.96	0.04	0.93
	100	1	0.91	0.98	0.87	0.98
	100	4	0.96	0.91	0.88	0.89
position likelihood	100	0.25	0.97	0.97	1	1
	100	1	0.98	0.98	0.99	0.99
	100	4	0.89	0.88	0.79	0.78

F.3 Estimating all parameters for the position likelihood

Figure A3 illustrates the ability of the position likelihood to estimate parameters across ranges of values for τ and ν, notably including estimates of the initial velocity v_0 (which are, however, of considerably less biological interest). At higher values of τ, the estimates of the initial velocity were much better than at lower values, since more observations "carry over" the information about the initial velocity. At low values of τ there was less accuracy for the v_0 estimates, but that had little effect on the precision of the τ and ν estimates themselves.

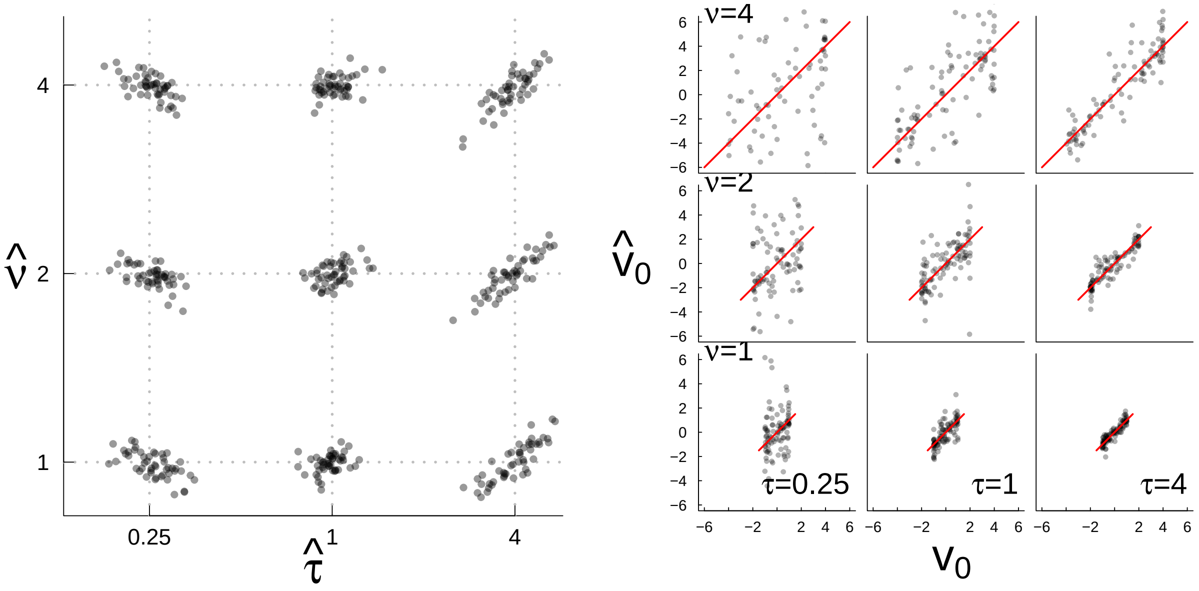

Figure A3: Predictions for ν and τ using the position likelihood method. 50 simulations were performed at each combination of given ν and τ values with initial velocities v_0 were drawn uniformly randomly from a uniform radial distribution and magnitude ν. 200 observations were sampled from each trajectory, with an exponentially distributed time gap between observations of mean 1. Each cloud in the upper panel is centered on the correct combination of initial parameters. The lower panel illustrates the estimates of initial velocities for each of the τ and ν combinations. The x and y components of $\mathbf{v}_0$ are presented simultaneously; the thick lines are the $y = x$ line.

G Change points analysis robustness

In order to assess the robustness of the CVM change point analysis, we analyzed random subsamplings of the bowhead data. We took 100%, 75%, 50% and 25% of the data points ($n = 954, 716, 477, 239$, respectively). We used a window size of 100 for the complete data, and proportionally smaller windows for the subsampled data (75, 50 and 25), fitting the UCVM, i.e. estimating only τ and η.

Figure A4 illustrates the relative likelihood plots for all four subsamplings. These plots show the likelihood for every potential break for every analysis window (rainbow colors) as it is swept across the movement time series (see also the vignette for the `smoove` package in the Supplementary Materials). Peaks in the plots correspond to candidate change points, which are then separately assessed via BIC. Figure A5 illustrates scatterplots of the parameter estimates for all observations across the two analyses. In this figure, the size of the points is proportional to the number of locations in the time series that are shared within each phase (e.g. if the analyses classify phases as: {1,1,1,2,2,2} and {1,1,2,2,2,3}, resp., the 1-1 and 2-2 points will be twice as large as the 1-2 and 2-3 points).

Although there is some variability - increasing with the sparser subsamplings - many of the change points are robustly identified across the four subsamplings (e.g. the four changes in quick succession between 80 and 120 h, around 200, 320 and 400 h). The sensitivity of the method is similar across samplings (due to the proportional shrinkage of the window size), leading to similar numbers of phases identified (respectively: 12, 13, 15 and 15). The correlation in predicted r.m.s. speed was very high across samplings (0.89, 0.84 and 0.86, figure A5), and somewhat lower for τ (between 0.58 and 0.8). There was an (unsurprising) slight bias for lower speed estimates at smaller subsamplings (compare regression lines to dotted grey lines), but the τ estimates regression, where less precise and more variable, seemed to be less biased overall.

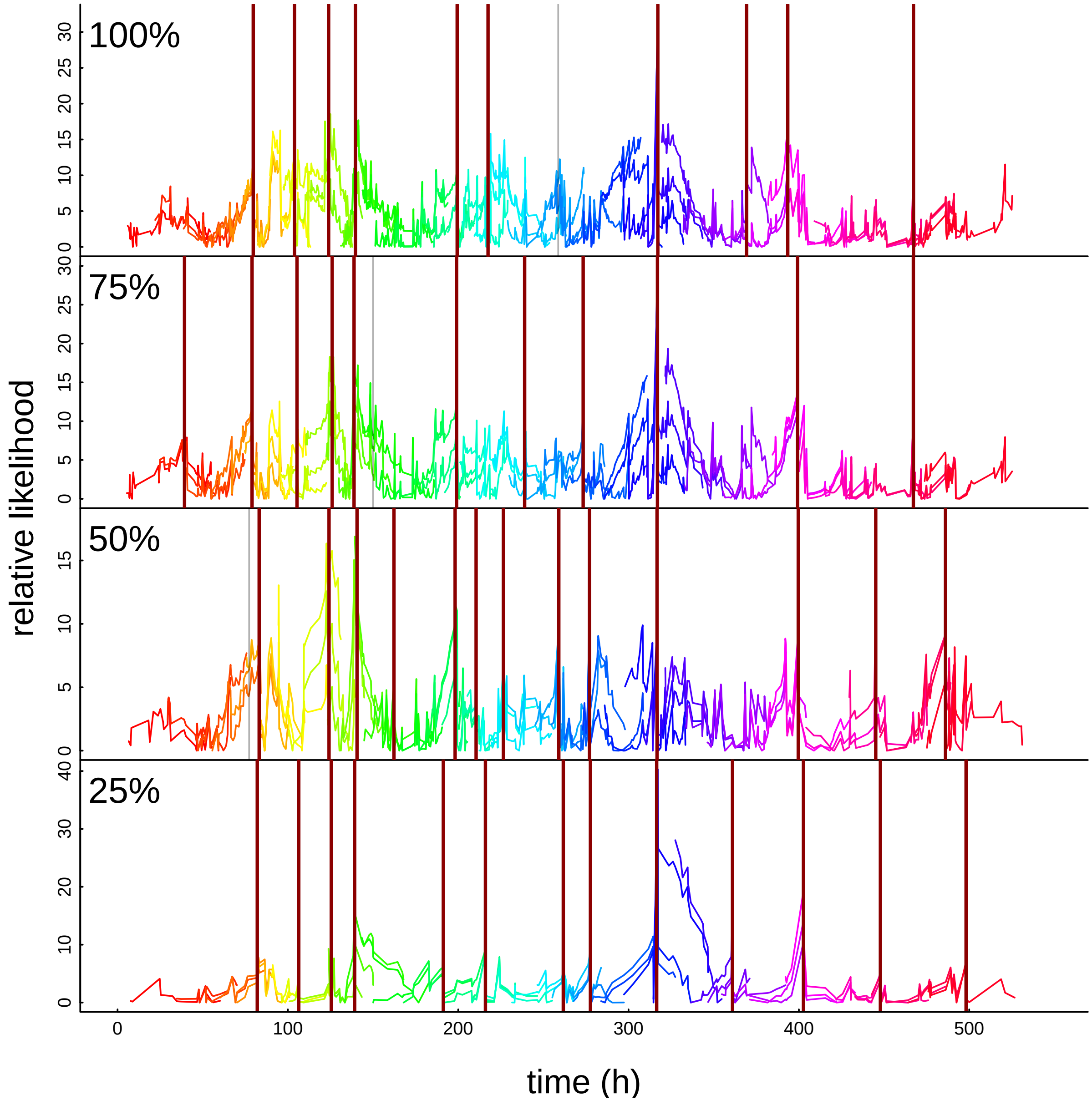

Figure A4: Relative likelihood plots of the change point analysis for 100%, 75%, 50% and 25% subsamplings of the data. Each color represents the likelihood profile for a single window. The vertical bars represent significant change points according to BIC.

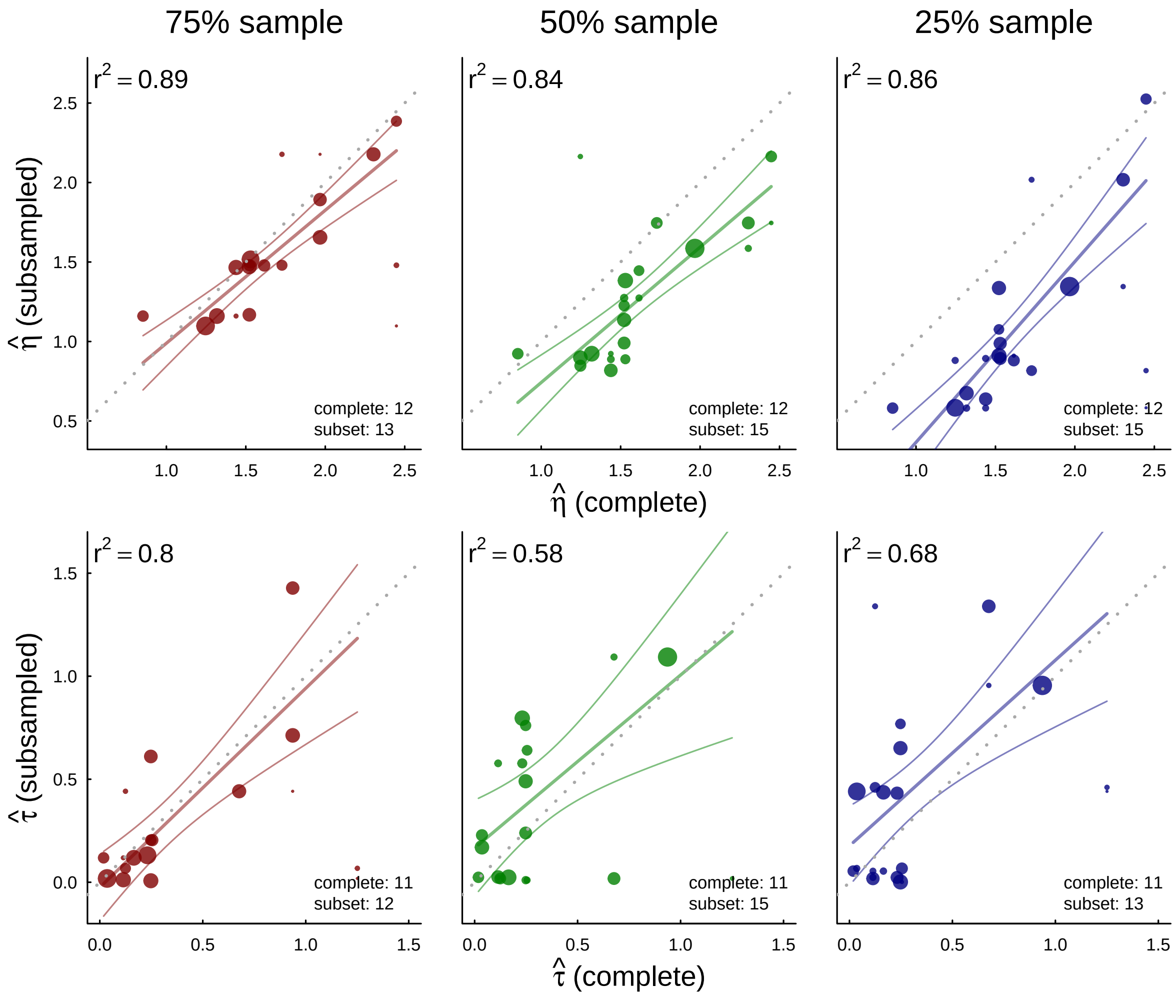

Figure A5: Scatterplots and r^2 values comparing the predicted values of time scale τ and rms speed η for a change point analysis for the complete and (left to right) the 75%, 50% and 25% subsampling. The dotted grey line is the 0,1 line of perfect fit, and the colored lines are the 95% prediction intervals around the (thicker) regression line. The size of the points is proportional to the number of locations in the time series that are shared within each phase (see text for details).

References

[1] Johnson, D.S., London, J.M., Lea, M.-A., Durban, J.W.: Continuous-time correlated random walk model for animal telemetry data. Ecology **89**(5), 1208–1215 (2008). http://www.esajournals.org/doi/pdf/10.1890/07-1032.1

[2] Gurarie, E., Grünbaum, D., Nishizaki, M.: Estimating 3d movements from 2d observations using a continuous model of helical swimming. Bulletin of Mathematical Biology **73**(6), 1358–1377 (2011). doi:10.1007/s11538-010-9575-7. 10.1007/s11538-010-9575-7

[3] Gurarie, E., Ovaskainen, O.: Characteristic spatial and temporal scales unify models of animal movement. The American Naturalist **178**, 113–123 (2011). doi:10.1086/660285

[4] Gillespie, D.T.: Exact numerical simulation of the Ornstein-Uhlenbeck process and its integral. Physical review E **54**(2), 2084–2091 (1996)

[5] Rice, S.O.: Mathematical analysis of random noise. Bell System Technical Journal, 46–156 (1945)

[6] Alt, W.: Correlation analysis of two-dimensional locomotion paths. In: Alt, W., Hoffmann, G. (eds.) Biological Motion: Proceedings of a Workshop Held in Königswinter Germany, pp. 254–268. Springer, Berlin (1990)

[7] Gurarie, E., Ovaskainen, O.: Towards a general formalization of encounter rates in ecology. Theoretical Ecology **6**, 189–202 (2013). doi:10.1007/s12080-012-0170-4

[8] Fleming, C.H., Calabrese, J.M., Mueller, T., Olson, K.A., Leimgruber, P., Fagan, W.F.: Likelihood estimation of autocorrelated movement processes. Methods in Ecology and Evolution (2014). doi:10.1111/2041-210X.12176. doi:10.1111/2041-210X.12176

[9] Alt, W.: Modelling of Motility in Biological Systems. In: McKenna, J., Temam, R. (eds.) ICIAM ’87: Proceedings of the First International Conference on Industrial and Applied Mathematics, pp. 15–30. SIAM, Philadelphia (1988)

[10] Takagi, H., Sato, M.J., Yanagida, T., Ueda, M.: Functional analysis of spontaneous cell movement under different physiological conditions. PLoS ONE **3**(7), 2648 (2008). doi:10.1371/journal.pone.0002648

[11] Venables, W.N., Ripley, B.D.: Modern Applied Statistics with S, 4th Edition. Springer, New York (2002)

[12] Pinheiro, J., Bates, D., DebRoy, S., Sarkar, D., R Core Team: `nlme`: Linear and nonlinear mixed effects models. (2013). R package version 3.1-110

[13] Johnson, D.S.: Crawl: Fit Continuous-time Correlated Random Walk Models to Animal Movement Data. (2013). R package version 1.4-1. http://CRAN.R-project.org/package=crawl
