# Supplementary material for: Correlated velocity models as a fundamental unit of animal movement: synthesis and applications
Source: Mov Ecol. 2017 May 10;5:13. doi: 10.1186/s40462-017-0103-3 (PMC5424322; doi:10.1186/s40462-017-0103-3)
Supplement: Supplementary file 2 — Vignette document (pdf) describing the functions and tools in the smoove, including replication of many of the results in the manuscript. (PDF 2375.68 kb) [file 40462_2017_103_MOESM2_ESM.pdf]

Smooth swimming and fanciful flights: Using the `smoove` package

Eliezer Gurarie
Department of Biology
University of Maryland
College Park, MD

November 22, 2016

Contents

1 Correlated velocity movement models **2**

2 Simulation **2**

2.1 Unbiased CVM 2

2.2 Rotational-Advective CVM 4

3 Empirical velocity auto-correlation function **5**

4 Estimation of CVM models **7**

4.1 UCVM 7

4.2 RACVM 11

4.3 Kestrel data 13

5 Behavioral change point analyses **15**

5.1 Identifying a single change point 15

5.2 Multiple change points 17

6 Kestrel data BCPA **21**

Abstract

The `smoove` package is a collection of functions to work with continuous time correlated velocity movement (CVM) models as described in Gurarie et al. (in review, *Movement Ecology*). There are

functions that simulate, diagnose and estimate these movement models, as well as functions for facilitating a change point analysis.

1 Correlated velocity movement models

Table 1: Summary of CVM models

Model	α	$\boldsymbol{\mu}$	Notation
Unbiased CVM	$1/\tau$	0	UCVM(τ, ν)
Advective CVM	$1/\tau$	non-zero	ACVM$(\tau, \eta, \mu_x, \mu_y)$
Rotational CVM	$1/\tau + i\omega$	0	RCVM(τ, η, ω)
Rotational-Advective CVM	$1/\tau + i\omega$	non-zero	RACVM$(\tau, \eta, \omega, \mu_x, \mu_y)$

Briefly, CVM models are movement models in which the velocity is assumed to be an autocorrelated stochastic process taking the form of an Ornstein-Uhlenbeck equation:

$$\begin{aligned} d\mathbf{v} &= \alpha(\boldsymbol{\mu} - \mathbf{v})\, dt + \frac{\eta}{\sqrt{\tau}}\, d\mathbf{w}_t, \\ \mathbf{v}(0) &= \mathbf{v}_0, \end{aligned} \quad (1)$$

where the key parameters are τ - the characteristic time scale of autocorrelation, η - the root mean squared speed of the movement process. Different valeus of the additional parameters α and $\boldsymbol{\mu}$ are related to variations of a CVM process, as per table 1. We present the models together with functions that estimate them sequentially below.

2 Simulation

2.1 Unbiased CVM

The unbiased CVM is the continuous time equivalent of an unbiased correlated random walk, i.e. a movement that has local persistence in direction. This is the most "fundamental" CVM model and its mean speed is given by a simple expression $\nu = \eta\sqrt{\pi}/2$, the a convenient parmeterization is as: UCVM(τ, ν). Also, it is for this model that the complete set of estimation methods discussed in the paper is available. For these reasons, it is treated slightly differently than the (R/A)CVM models in the `smoove` package.

The function for simulating the UCVM is `simulateUCVM`. There are two methods of simulation, *direct* and *exact*.

2.1.1 Direct method

The direct method works by discretizing the differential equation 1, i.e.:

$$V_{i+1} = V_i - V_i(dt/\tau) + \frac{2\nu}{\sqrt{\pi\tau}} dW_i$$

where V is a vector of complex numbers and dW_i is drawn from a bivariate normal distribution with variance $\sqrt{dt}$. The direct method provides good simulations relatively quickly at high resolution ($\Delta t \ll \tau$).

Loading the package:

```
require(smoove)
```

Note, the package has many dependencies related to matrix manipulations.

An example of a track with $\nu = 2$, $\tau = 5$, for a time interval of 1000:

```r
nu <- 2
tau <- 5
dt <- .1
ucvm1 <- simulateUCVM(nu=nu, tau=tau, T.max = 1000, dt = dt)
```

The resulting object contains complex velocity and position vectors, a time vector, an XY matrix, and the values of the simulated parameters.

```r
str(ucvm1)

## List of 5
##  $ T          : num [1:10001] 0 0.1 0.2 0.3 0.4 0.5 0.6 0.7 0.8 0.9 ...
##  $ V          : cplx [1:10001] 0.46+1.95i 0.43+2.35i 0.79+1.65i ...
##  $ XY         : num [1:10001, 1:2] 0.0463 0.0893 0.1688 0.3215 0.5341 ...
##   ..- attr(*, "dimnames")=List of 2
##   .. ..$ : NULL
##   .. ..$ : chr [1:2] "x" "y"
##  $ Z          : cplx [1:10001] 0.046+0.195i 0.089+0.429i 0.169+0.594i ...
##  $ parameters:List of 3
##   ..$ tau: num 5
##   ..$ nu : num 2
##   ..$ v0 : cplx 0.46+1.95i
```

The curve can be plotted in several ways:

```r
plot(ucvm1$Z, asp=1, type="l", main = "plotting Z")
plot(ucvm1$XY, asp=1, type="l", main = "plotting XY")
plot.track(ucvm1$XY, col=rgb(0,0,0,.01), main = "using plot.track")
```

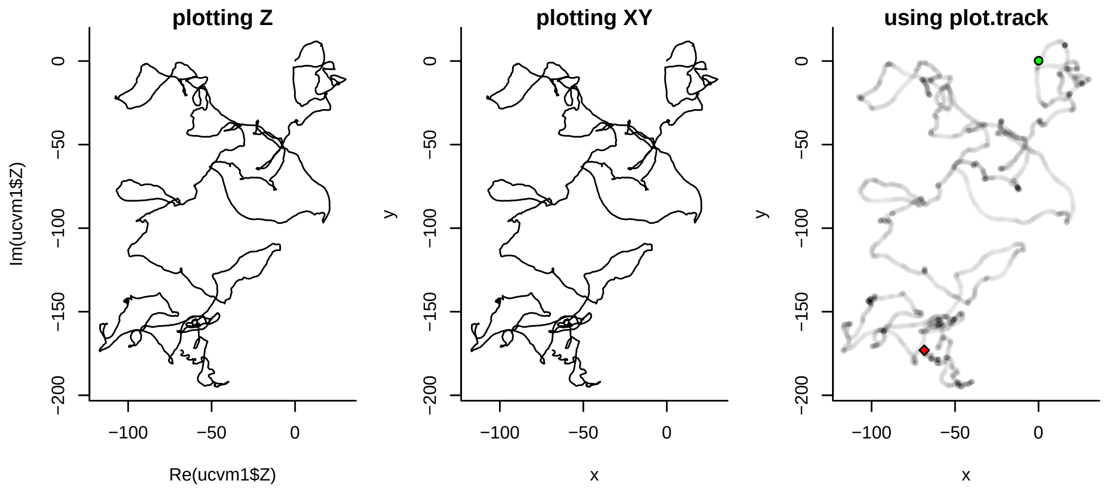

The `plot.track` function is a simple convenience function for plotting tracks. According to theory, the mean speed of this track should just be $\nu = 2$:

```r
mean(Mod(ucvm1$V))

## [1] 2.006701
```

and the root mean square speed should be $2\nu/\sqrt{\pi} = 2.2567583$:

```r
sqrt(mean(Mod(ucvm1$V)^2))

## [1] 2.244111
```

2.1.2 Exact method

The *exact* method is more flexible, as it samples the position and velocity process simultaneously from the complete mean and variance-covariance structure of the integrated OU process. The main advantage is that the process can be simulated for irregular (and totally arbitrary) times of observation:

```
T <- cumsum(rexp(100))
ucvm2 <- simulateUCVM.exact(T = T, nu = 2, tau = 2)
with(ucvm2, scan.track(time = T, z = Z))
```

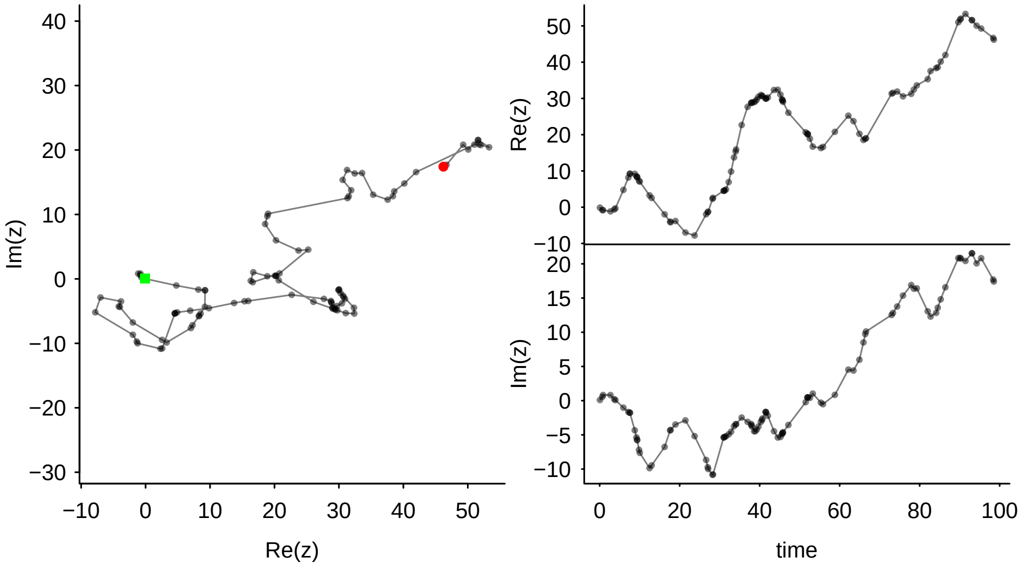

The **scan.track** function is another function that is convenient for seeing a process in time (right plots). Note the irregularity of the sampling.

```
summary(diff(ucvm2$T))

##     Min.  1st Qu.   Median     Mean  3rd Qu.     Max.
## 0.008147 0.260100 0.614000 0.994600 1.355000 6.402000
```

and that the velocity vector has the correct statistical properties (mean and 95% C.I.):

```
mean(Mod(ucvm2$V))

## [1] 2.1223

with(ucvm2, mean(Mod(V)) + c(-2,2)*sd(Mod(V)) / sqrt(length(V)))

## [1] 1.896336 2.348265
```

2.2 Rotational-Advective CVM

The (R/A)CVM models are parameterized in terms of τ, the random rms speed η, and then some combination of the advection vector $\boldsymbol{\mu}$ and/or angular speed ω.

```
rcvm <- simulateRACVM(tau = 6, eta = 2, omega = 1, mu = 0, Tmax = 1000, dt = .1)
acvm <- simulateRACVM(tau = 6, eta = 2, omega = 0, mu = 2, Tmax = 1000, dt = .1)
racvm <- simulateRACVM(tau = 6, eta = 2, omega = 1, mu = 1, Tmax = 1000, dt = .1)
```

```r
plot.track(rcvm$Z[rcvm$T < 100],  main = "RCVM")
plot.track(acvm$Z[acvm$T < 100], main = "ACVM")
plot.track(racvm$Z[racvm$T < 100], main = "RACVM")
```

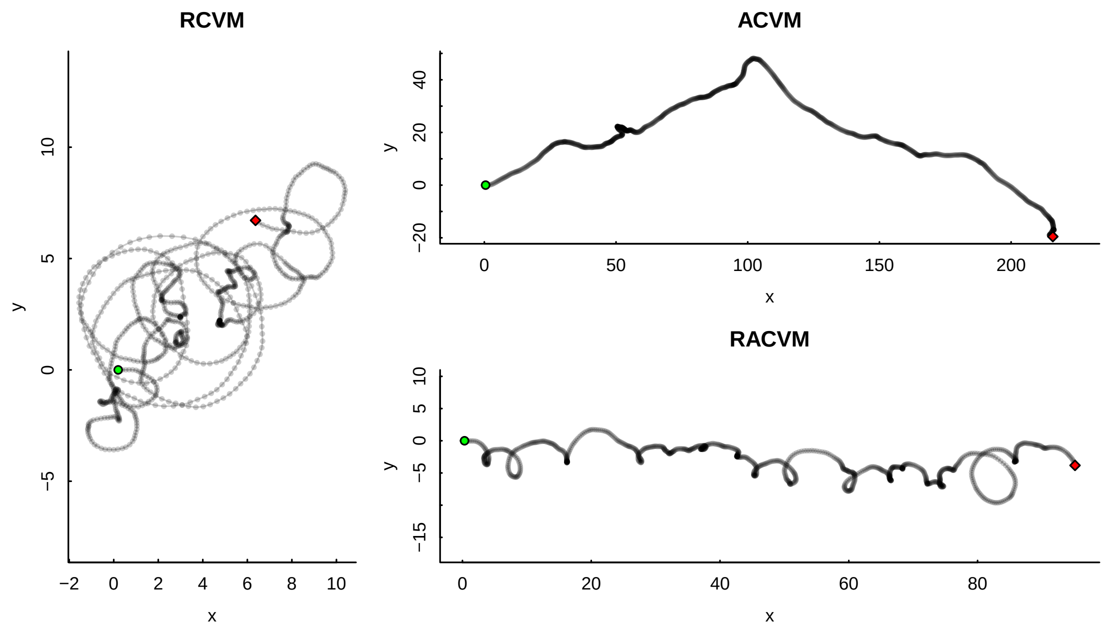

Note that we only plot these curves within the first 100 time units. For the moment, the RACVM models can only be simulated "directly", i.e. via direct forward simulations rather than sampling from the complete autocorrelated process.

3 Empirical velocity auto-correlation function

The velocity autocovariance function (VAF) is a very useful way to visualize the autocorrelation structure of the movement models. It is defined as the expectation of a dot product of the velocity vector with itself as a function of lags:

$$C_v(\Delta t) = \mathrm{E}\left[\mathbf{v}(t_0 + \Delta t) \cdot \mathbf{v}(t_0)\right], \tag{2}$$

S At lag 0, the value is mean squared speed of the process, and at long lags it is the square of the mean drift of the process. For CVM models, $C_v(0) = \eta^2 + \mu^2$, $\lim_{\Delta t \to \infty} C_v(\Delta t) = \mu^2$, and the function decays exponentially at rate τ with an oscillatory component with angular velocity ω. The main functions for computing the empirical VAF is the `getEVAF` function:

```r
ucvm.vaf <- with(ucvm1, getEVAF(Z = Z, T = T, lagmax = 30))
acvm.vaf <- with(acvm, getEVAF(Z = Z, T = T, lagmax = 30))
rcvm.vaf <- with(rcvm, getEVAF(Z = Z, T = T, lagmax = 30))
racvm.vaf <- with(racvm, getEVAF(Z = Z, T = T, lagmax = 30))
```

This function produces a two column data frame with the lag and the respective computed evaf:

```r
head(ucvm.vaf)

##   lag      vaf
## 1 0.0 5.036137
## 2 0.1 4.932982
```

```
## 3 0.2 4.834510
## 4 0.3 4.737064
## 5 0.4 4.641500
## 6 0.5 4.548041
```

A plot of the four curves and their respective empirical autocovariance functions:

```
plot.track(ucvm1$Z[1:1e3], main = "UCVM"); plot(ucvm.vaf, type="l", lwd=1.5)
plot.track(acvm$Z[1:1e3], main = "ACVM"); plot(acvm.vaf, type="l", lwd=1.5)
plot.track(rcvm$Z[1:1e3], main = "RCVM"); plot(rcvm.vaf, type="l", lwd=1.5)
plot.track(racvm$Z[1:1e3], main = "RACVM"); plot(racvm.vaf, type="l", lwd=1.5)
```

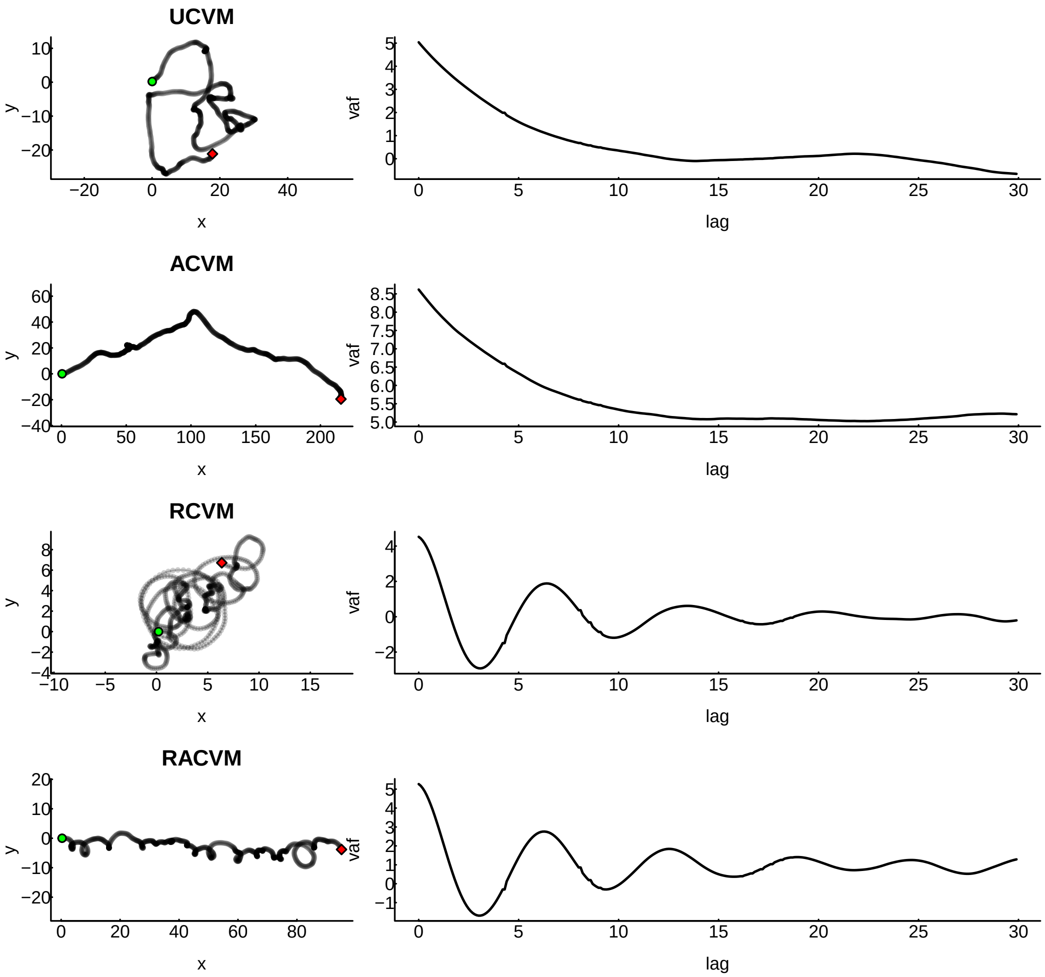

The velocity autocovariance functions is a very useful visual summary of a movement track because it refeals . Furthermore These curves can be used to estimate the parameters of these processes.

4 Estimation of CVM models

The main estimation functions are `estimateUCVM` and `estimateRACVM`. The respective help files have abundant examples of implementation.

4.1 UCVM

Following the structure of the Gurarie at al. (*in review*), manuscript, the UCVM, which focusses on estimates of time scale τ and speed ν, can be estimated using one of five different methods.

4.1.1 VAF fitting

This method relies on fitting the *empirical* velocity autocorrelation function (above) to the *theoretical* velocity autocorrelation function:

$$C_v(\Delta t) = \frac{4}{\pi}\nu^2 \exp(\Delta t/\tau)$$

```
estimateUCVM(z = ucvm1$Z, t = ucvm1$T, method = "vaf", diagnose = TRUE, CI=TRUE)
```

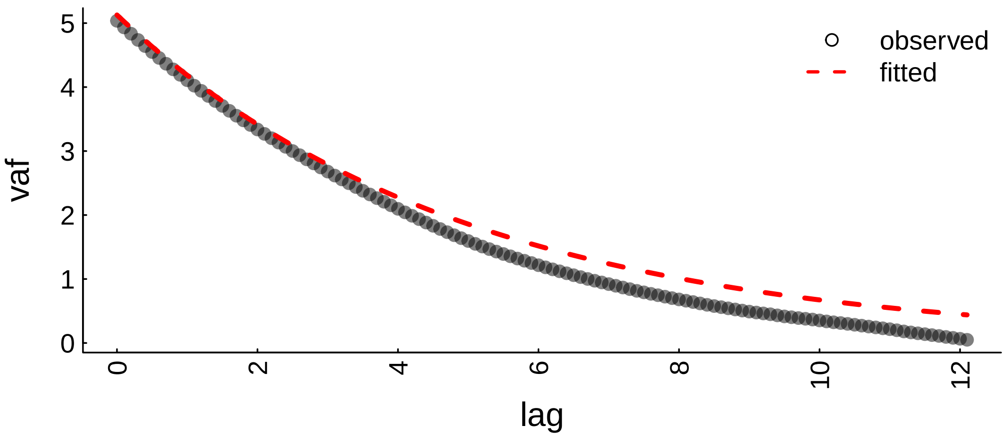


```
##     Estimate  C.I.low C.I.high
## tau 4.926240 4.867296 4.986628
## nu  2.006702 1.810426 2.202978
```

The diagnostic plot (only plotted with `diagnose=TRUE`) illustrates the quality of the fit. The estimates should be fairly good, when compared to the true parameters:

```
ucvm1$parameters[1:2]

## $tau
## [1] 5
##
## $nu
## [1] 2
```

A lower-resolution track will have wider confidence intervals:

```r
ucvm.lores <- simulateUCVM(nu=10, tau = 4, dt = 1, T.max = 1000, method = "exact")
with(ucvm.lores, estimateUCVM(z = Z, t = T, CI=TRUE, method="vaf"))
```

```
##      Estimate  C.I.low C.I.high
## tau  8.184796 4.927986 24.13544
## nu  10.135932 9.452142 10.81972
```

The speed estimate might be improved by using a spline correction

```r
with(ucvm.lores, estimateUCVM(z = Z, t = T, CI=TRUE, method="vaf", spline = TRUE))
```

```
##      Estimate  C.I.low C.I.high
## tau  5.515561 3.573607 12.08006
## nu  10.324379 9.769988 10.87877
```

NB: This method works only for regularly sampled data, and the confidence intervals tend to be unreliable.

4.1.2 CRW matching

The Correlated Random Walk (CRW) matching method computes the CRW parameters (shape and scale of length steps and wrapped Cauchy clustering coefficient) and converts those to time-scales and speed estimates. Confidence intervals are obtained by Monte Carlo draws from the confidence intervals around likelihood estimates of the CRW parameters.

```r
tau <- 2; nu <- 8
ucvm3 <- simulateUCVM(T=1:1000, nu = nu, tau = tau, method = "exact")
plot(ucvm3$Z, asp=1, type="o", cex=0.5, pch=19)
```

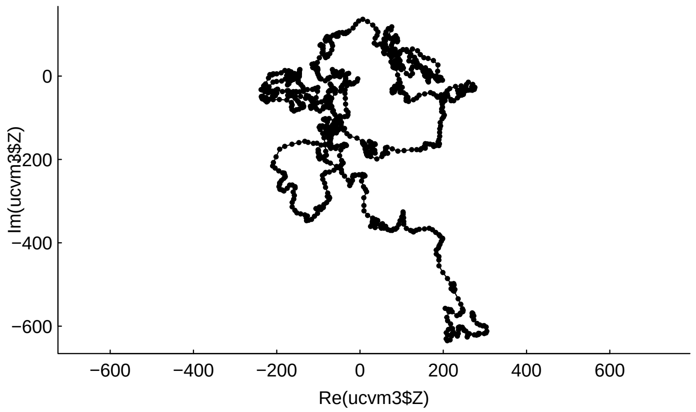


```r
estimateUCVM(z = ucvm3$Z, t = ucvm3$T, CI=TRUE, method="crw", diagnose=TRUE)
```

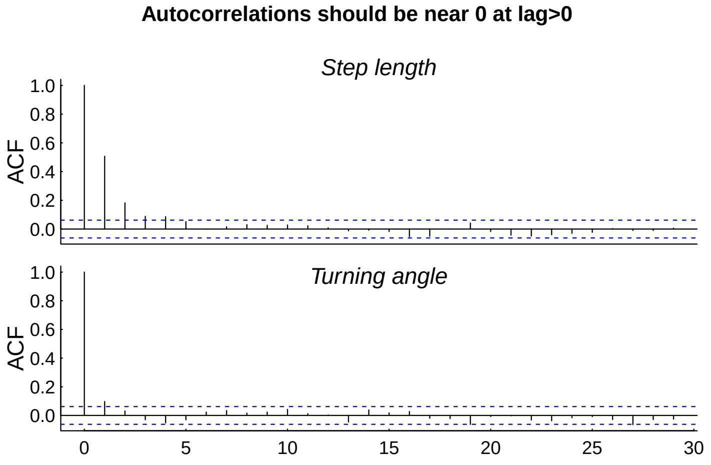


```
##     Estimate  C.I.low C.I.high
## tau 1.919065 1.721424 2.198559
## nu  6.214343 5.686990 6.715880
```

The diagnosis plots (crudely) illustrates the standard autocorrelation function for the step lengths and turning angles. Under the normal assumptions of the CRW, these should both be around 0 at lags greater than 0. The more autocorrelated either of these time series is, the more biased the estimates are likely to be.
NB: This method is illustrative, generally biased, and recommended except for data that are relatively coarsely sampled. It is, however, very fast to compute.

4.1.3 Velocity likelihood

The velocity likelihood method is based on using the known distributional properties of the integrated OU obtain a "one-step" likelihood of each velocity based on the previous velocity. This method is robust to irregularly sampled data and is fairly fast. We will estimate the original track

```
estimateUCVM(z = ucvm1$Z, t = ucvm1$T, method = "vLike", CI=TRUE)
```

```
##     Estimate  C.I.low C.I.high
## tau 4.829304 4.208526 5.541650
## nu  1.988235 1.852858 2.123612
```

This method gives very reliable confidence intervals, but may underestimate velocities somewhat. We can also run it on the irregularly sampled track from above:

```
estimateUCVM(z = ucvm2$Z, t = ucvm2$T, method = "vLike", CI=TRUE)
```

```
##     Estimate  C.I.low C.I.high
## tau 2.951035 1.941522 4.485455
## nu  1.976168 1.619847 2.332489
```

The confidence intervals are more wide this time (due to a much smaller data set: 100 versus 10001 data points), but the estimates should be fairly accurate.

4.1.4 Position likelihood

The position likelihood (`zLike`) takes the complete correlation structure between the velocities and the positions to estimate the parameters. It is prohibitively slow on a data set the size of `ucvm1`, but quite fast on the second (irregular, short) sampling:

```
estimateUCVM(z = ucvm2$Z, t = ucvm2$T, method = "zLike", CI=TRUE)

##       Estimate    C.I.low  C.I.high
## tau  2.094470  1.4632720  2.997941
## nu   2.159361  1.7361266  2.582596
## v0x -1.651185 -2.1744301 -1.127939
## v0y  1.347167  0.8239216  1.870413
```

Note, improvements in the parameter estimates and confidence intervals, and the inclusion of estimates for the initial speed (usually a parameter of less interest).

4.1.5 Position likelihood: Kalman filter

We (OO and EG) had independently worked out many of the details of the estimation of full position likelihood before we discovered that the same likelihood was estimated in a much more efficient way by Johnson et al. (2008), and encoded in an excellent package called "crawl" (*Correlated RAndom Walk Library*). We have incorporated a wrapper for this package in **smoove** for the estimation of UCVM parameters - a fairly narrow application of the capabilities of **crawl**. First, we run it on the irregular data set:

```
with(ucvm2, estimateUCVM(z = Z, t = T, method = "crawl"))

## Beginning SANN initialization ...
## Beginning likelihood optimization ...

## $fit
##
##
## Continuous-Time Correlated Random Walk fit
##
## Models:
## --------
## Movement   ~ 1
## Error
##
##
##                         Parameter Est. St. Err. 95% Lower 95% Upper
## ln sigma (Intercept)           1.257    0.155     0.953     1.562
## ln beta (Intercept)           -0.726    0.184    -1.086    -0.366
##
##
## Log Likelihood = 55.259
## AIC = -106.517
##
##
##
##
## $nutau
##     Estimate        L        U
## tau 2.067232 1.442205 2.963135
## nu  2.167422 1.599077 2.937767
```

The estimates and C.I.'s for ν and τ are very similar to the full position likelihood above. The additional output is specific to the parameterization Johnson et al. use, the lower output (**nutau**) is consistent with the output for the other methods.
The **crawl** method can handle a very long ($n = 10000$) dataset as well.

```
with(ucvm1, estimateUCVM(z = Z, t = T, method = "crawl"))$nutau

## Beginning SANN initialization ...
## Beginning likelihood optimization ...

##     Estimate        L        U
## tau 2.767653 2.491838 3.073997
## nu  1.999750 1.803014 2.217952
```

with correspondingly smaller confidence intervals. There are many additional options that can be passed to the `crawl` solver, detailed in the help file for `crwMLE`.

4.2 RACVM

Estimating the RACVM processes is different mainly in that there are now four different models to compare (with and without each of rotation and advection), and a model selection method is intrinsically built in. For the time being, the method implemented for fitting these models is the *velocity likelihood*. In the examples below, we estimate the parameters from each of the RACVM simulations and them from a portion of actual kestrel flight data:

```
ucvm <- simulateRACVM(tau = 5, eta = 2, omega = 0, mu = 0, Tmax = 100, dt = .1)
acvm <- simulateRACVM(tau = 5, eta = 2, omega = 0, mu = 2, Tmax = 100, dt = .1)
rcvm <- simulateRACVM(tau = 5, eta = 2, omega = 2, mu = 0, Tmax = 100, dt = .1)
racvm <- simulateRACVM(tau = 5, eta = 2, omega = 2, mu = 2, Tmax = 100, dt = .1)
```

4.2.1 Unbiased CVM

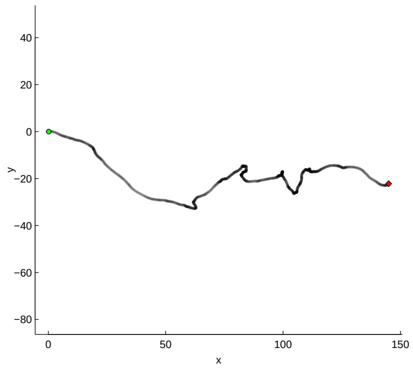


```
with(ucvm, estimateRACVM(Z=Z, T=T, compare.models=TRUE))

## $results
##                eta       omega      mu.x       mu.y      tau
## Estimate 1.688483 -0.03081577 1.4618791 -0.1893302 3.451136
## CI.low   1.362429 -0.13983355 0.8369856 -0.8137050 2.333424
## CI.high  2.014536  0.07820201 2.0867727  0.4350445 5.104232
##
## $LL
## [1] -309.4763
##
## $CompareTable
##         logLike k      AIC deltaAIC      BIC deltaBIC
## UCVM  -315.0095 2 634.0190 7.263868 643.8345 0.000000
## ACVM  -309.3776 4 626.7551 0.000000 646.3861 2.551642
## RCVM  -314.9840 3 635.9681 9.212973 650.6914 6.856860
## RACVM -309.4763 5 628.9526 2.197506 653.4914 9.656903
```

The estimation function (by default) fits the most complex RACVM model. The comparison table indicates that the most parsimonious model, according to both AIC and the more conservative BIC, is the UCVM. Note, also, that the confidence intervals around ω, μ_x and μ_y all include 0 - additional evidence that the model may not be advective or rotational. The following code estimates the "selected" model.

```
with(ucvm, estimateRACVM(Z=Z, T=T, model="UCVM", compare.models=FALSE))

## $results
##                eta       tau
## Estimate 2.231088  6.101591
## CI.low   1.681067  3.711691
## CI.high  2.781109 10.030310
```

```
##
## $LL
## [1] -315.0095
```

Every time this document is compiled, the results change somewhat. But in most cases the model selection should return the correct model and the estimated confidence intervals should include the true values ($\eta = 5, \omega = 2, \mu_x = 2, \mu_y = 0$).

4.2.2 Advective CVM

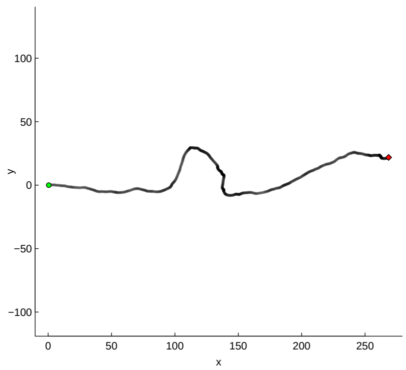


```
with(acvm, estimateRACVM(Z=Z, T=T, model = "ACVM",
                         compare.models=TRUE))

## $results
##                 eta     mu.x       mu.y      tau
## Estimate 2.210154 2.496207  0.2981604 5.897381
## CI.low   1.677453 1.418440 -0.7765446 3.627084
## CI.high  2.742856 3.573974  1.3728654 9.588723
##
## $LL
## [1] -329.6863
##
## $CompareTable
##          logLike k      AIC deltaAIC      BIC  deltaBIC
## UCVM  -334.1435 2 672.2869 4.914438 682.1025  0.000000
## ACVM  -329.6863 4 667.3725 0.000000 687.0035  4.901073
## RCVM  -334.0695 3 674.1389 6.766424 688.8622  6.759742
## RACVM -329.6182 5 669.2363 1.863818 693.7751 11.672646
```

4.2.3 Rotational CVM

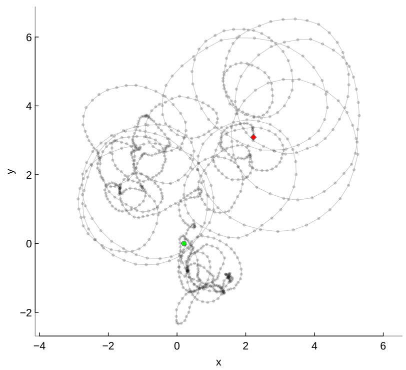


```
with(rcvm, estimateRACVM(Z=Z, T=T, model = "RCVM",
                         compare.models=TRUE))

## $results
##                 eta    omega      tau
## Estimate 2.225867 2.055675 5.825114
## CI.low   1.693867 1.973025 3.597220
## CI.high  2.757867 2.138325 9.432827
##
## $LL
## [1] -355.8245
##
## $CompareTable
##           logLike k      AIC    deltaAIC       BIC   deltaBIC
## UCVM  -1153.1937 2 2310.387 1592.738458 2320.2030 1587.83070
## ACVM  -1153.1857 4 2314.371 1596.722385 2334.0024 1601.63014
## RCVM   -355.8245 3  717.649    0.000000  732.3723    0.00000
## RACVM  -355.5865 5  721.173    3.524005  745.7118   13.33952
```

4.2.4 Rotational-Advective CVM

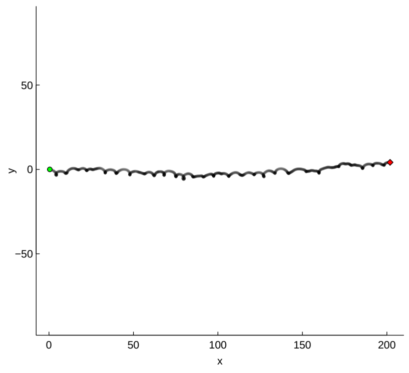


```
with(racvm, estimateRACVM(Z=Z, T=T, model = "RACVM",
                          compare.models=TRUE))

## $results
##                eta    omega     mu.x        mu.y      tau
## Estimate 1.854341 1.927175 2.016319  0.05260617 4.140568
## CI.low   1.483918 1.829585 1.922283 -0.04142948 2.763997
## CI.high  2.224763 2.024766 2.110355  0.14664182 6.202723
##
## $LL
## [1] -325.1292
##
## $CompareTable
##          logLike k       AIC  deltaAIC       BIC  deltaBIC
## UCVM  -907.2552 2 1818.5105 1158.2521 1828.3260 1143.5289
## ACVM  -896.2117 4 1800.4235 1140.1651 1820.0545 1135.2574
## RCVM  -683.6415 3 1373.2831  713.0247 1388.0064  703.2092
## RACVM -325.1292 5  660.2584    0.0000  684.7971    0.0000
```

4.3 Kestrel data

In the paper, we analyze a phenomenal data set of a single lesser kestrel (*Falco naumanni*) flight in southern Spain from Hernández-Pliego et al (2015a, 2015b). One kestrel's flight is in the package:

```
data(Kestrel); plot.track(Kestrel[,c("X","Y")], cex=0.3)
```

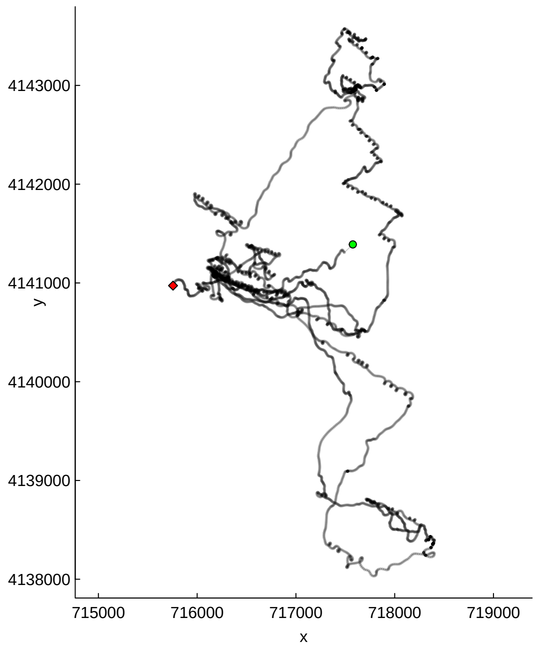

We can analyze one portions of the kestrel's flight, mirroring the analysis in Gurarie (in review). The following 90 second snippet is clearly advective and rotational:

```
K1 <- Kestrel[3360:3450,]
with(K1, scan.track(x=X, y=Y))
```

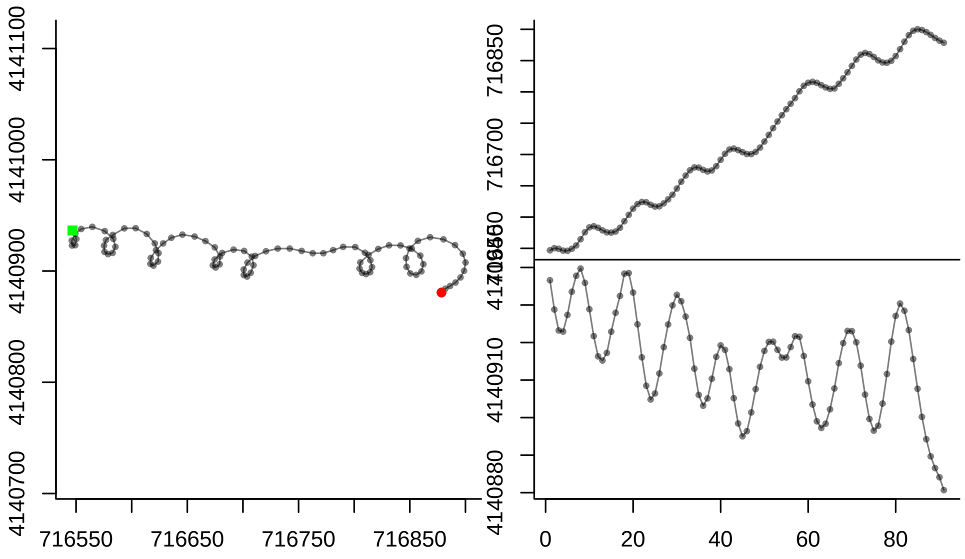

The model selection confirms this (note that we specify the time unit of analysis - in this case, seconds).

```
(fit1 <- with(K1, estimateRACVM(XY = cbind(X,Y), T = timestamp, spline=TRUE,
                                model = "RACVM", time.units = "sec")))

## $results
##                 eta     omega     mu.x       mu.y        tau
## Estimate  7.588318 0.5088868 3.867659 -0.3625973  31.508582
## CI.low    3.044545 0.4702505 3.299105 -0.9311530   9.331181
## CI.high  12.132090 0.5475230 4.436212  0.2059585 106.394968
##
## $LL
## [1] -303.429
##
## $CompareTable
##         logLike k      AIC deltaAIC      BIC  deltaBIC
## UCVM  -439.6961 2 883.3922 266.5342 888.3918 259.03478
## ACVM  -438.7136 4 885.4271 268.5691 895.4263 266.06930
## RCVM  -357.2247 3 720.4495 103.5915 727.9489  98.59185
## RACVM -303.4290 5 616.8580   0.0000 629.3570   0.00000
```

The track is very regularly but very slightly coarsely sampled. We therefore use the spline correction (though its impact is probably minimal). The model selection strongly prefers an RACVM model. The rotational speed is about 0.5 radians per second, the advective speed is aruond 4 m/sec. We feed these estimates back into the simulation algorithm to simulate a trajectory:

```
p.fit1 <- with(fit1$results, list(eta=eta[1], tau = tau[1],
                                  mu = mu.x[1] + 1i*mu.y[1],
                                  omega = omega[1], v0 = diff(K1$Z)[1]))
K1.sim <- with(p.fit1, simulateRACVM(eta=eta, tau=tau, mu = mu, omega=omega,
                                     Tmax = nrow(K1), v0 = v0, dt = 1))
with(K1.sim, scan.track(z=Z))
```

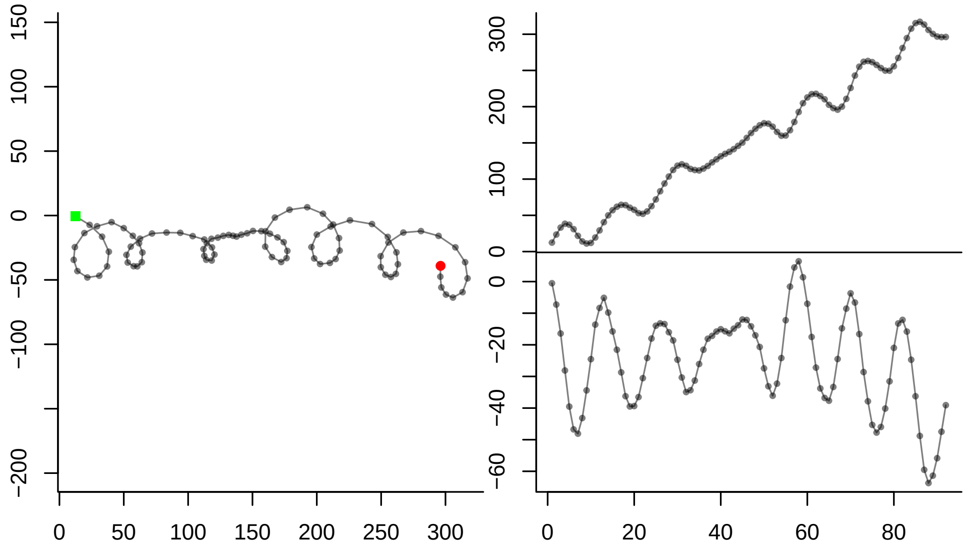

Visually, the simulation looks rather similar with respect to radii and frequence of rotations, randomness and advective tendency to the data snippet.

5 Behavioral change point analyses

Change point analysis (like most of ecological analysis) is not an exact science. However, the existence of likelihoods and model selection criteria makes it possible to develop an heuristic that can propose and assess candidate change points, select "significant" ones, and estimate the movement model and parameters between those change points.

5.1 Identifying a single change point

Simulate a two-phase UCVM:

```
ucvm1 <- simulateUCVM(T=cumsum(rexp(100)), nu=2, tau=1, method="exact")
ucvm2 <- simulateUCVM(T=cumsum(rexp(100)), nu=2, tau=10, v0 = ucvm1$V[100], method="exact")
T <- c(ucvm1$T, ucvm1$T[100] + ucvm2$T)
Z <- c(ucvm1$Z, ucvm1$Z[100] + ucvm2$Z)
plot.track(Z)
```

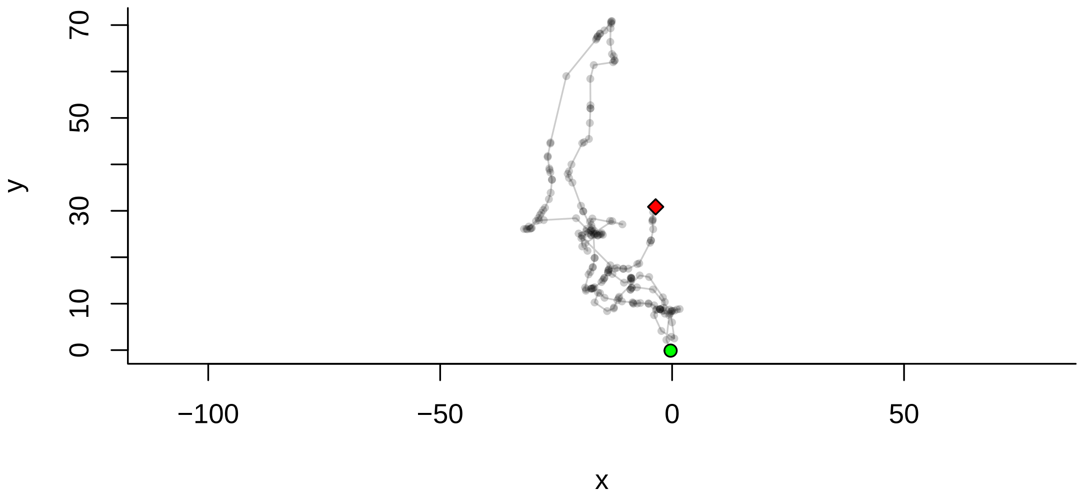

The only parameter that changed in this simulation is the time scale - it is not necessarily easy to identify by eye where the model switched. The likelihood of all candidate change points within this track is found using the `findSingleBreakPoint` as follows:

```
findSingleBreakPoint(Z,T, method = "sweep")
```

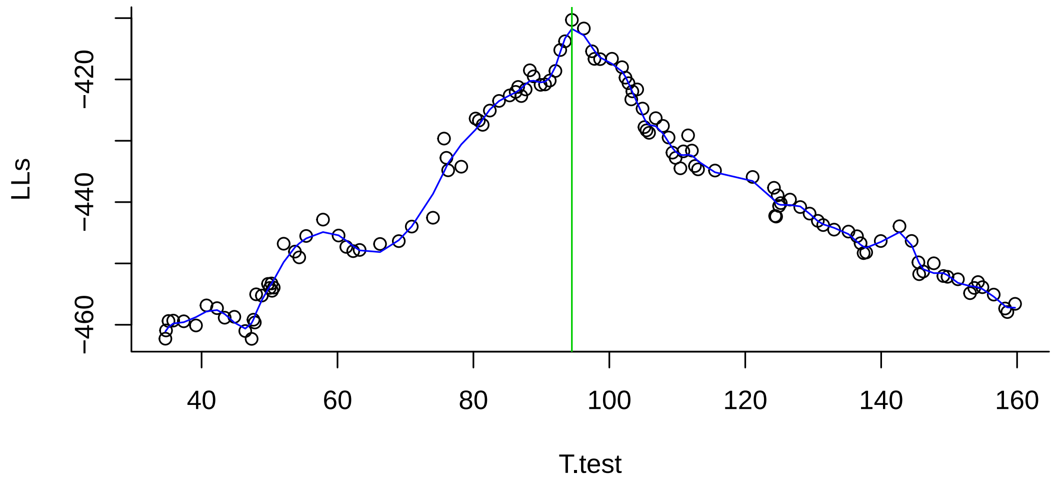


```
## [1] 94.48907
```

The true change point occured at time:

```
max(ucvm1$T)

## [1] 97.97526
```

5.2 Multiple change points

To find multiple change points where the number of changes is unknown *a priori*, we sweep change point analysis windows across the entire time series to obtain candidate change points. Simulate an all-UCVM trajectory with 4 behavioral phases:

```
ucvm1 <- simulateUCVM(T=cumsum(rexp(100)), nu=2, tau=1, method="exact")
ucvm2 <- simulateUCVM(T=cumsum(rexp(100)), nu=5, tau=5, v0 = ucvm1$V[100], method="exact")
ucvm3 <- simulateUCVM(T=cumsum(rexp(100)), nu=5, tau=1, v0 = ucvm2$V[100], method="exact")

T <- c(ucvm1$T, ucvm1$T[100] + ucvm2$T, ucvm1$T[100] + ucvm2$T[100] + ucvm3$T)
Z <- c(ucvm1$Z, ucvm1$Z[100] + ucvm2$Z, ucvm1$Z[100] + ucvm2$Z[100] + ucvm3$Z)
plot.track(Z)
```

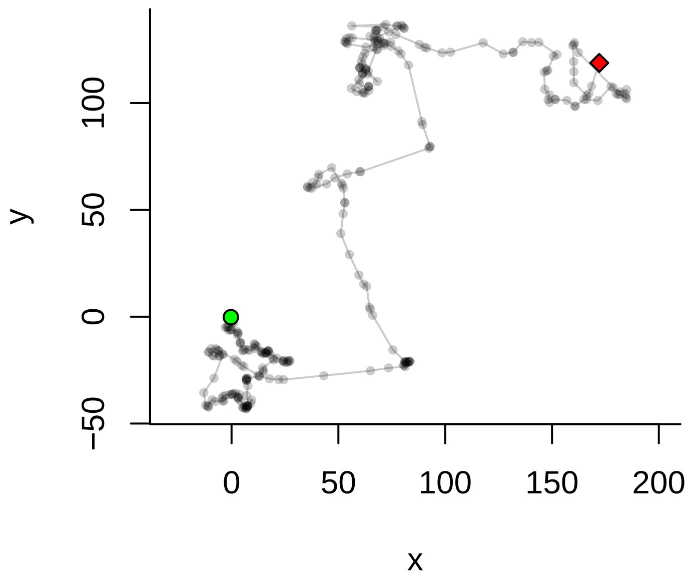

The actual change points occur at times 88.31 and 181.55.

5.2.1 Step I - the Window Sweep

TO perform a window sweep, we need to set two variables: the windowsize of analysis and the windowstep. Determining the appropriate windowsize is black magic, i.e. should be driven by "biological" considerations. As a rule of thumb, less than 30 is too short.

```
simSweep <- sweepRACVM(Z=Z, T=T, windowsize = 100, windowstep = 10, model = "UCVM", progress=FALSE)
```

Toggling the `progress` to TRUE will output a series of progress bars in the R console.

```r
plotWindowSweep(simSweep)
```

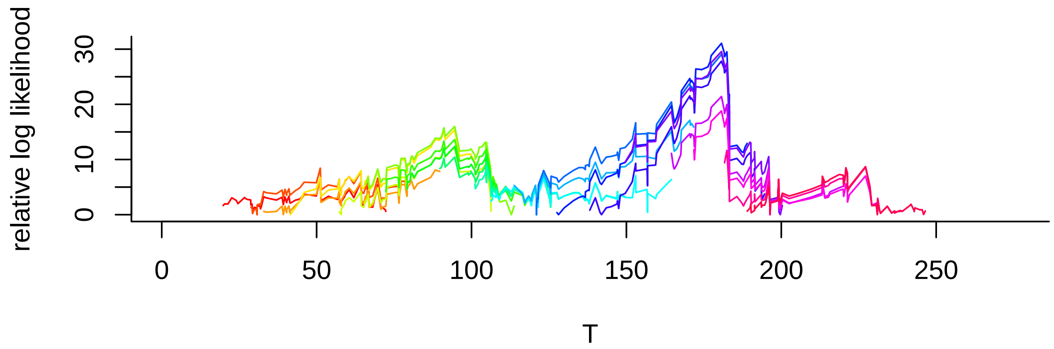

In the image above, each color represents the relative log-likelihood profile for a single window. There are two distinct peaks, the remainder of the algorithm boils these down to "significant" change points.

5.2.2 Step II - Obtain Candidate Change Points

We obtain candidate change points by taking all of the most likely change points (MLCP) in each of the windows, listing all of the MLCP's. The function that does this is `findCandidateChangePoints`. All the candidates:

```r
CP.all <- findCandidateChangePoints(windowsweep = simSweep, clusterwidth = 0)

## Warning in findCandidateChangePoints(windowsweep = simSweep, clusterwidth = 0):
##  Some of your partitions are very small - probably too small.  You might consider re-clustering
the change points with a threshold of at least 1.75.

CP.all

##  [1]  51.18221  88.30959  91.09221  94.57748 104.80393 153.00793 170.47928
##  [8] 180.69264 182.44672 227.19244
```

The warning lets us know that some of the candiate change points are rather close to each other, and it might be a good idea to cluster them, i.e. assign multiple points within a given time interval to a single cluster. If we take a rather generous cluster width below:

```r
CP.clustered <- findCandidateChangePoints(windowsweep = simSweep, clusterwidth = 4)
CP.clustered

## [1]  51.18221  93.18417 180.94322
```

We now have a more limited set of change points to assess.

5.2.3 Step III - Selecting Significant Change Points

We use the set of candidate change points to separate the track into phases and for each change point (i.e. pair of neighboring phases) we assess the "significance" of the change point by comparing the BIC (or AIC) of a two-model versus one-model fit. The function that performs this is `getCPtable`

```r
getCPtable(CPs = CP.clustered, Z = Z, T = T, modelset = "UCVM", tidy = FALSE, iterate = FALSE)

##           CP     start       end      dAIC     dBIC extremes   M1   M2
## 1   51.18221  1.181941  93.18417  15.86907  7.93590           UCVM UCVM
```

```
## 2  93.18417 51.182212 180.94322  33.27595 24.38741  eta-tau UCVM UCVM
## 3 180.94322 93.184175 275.47416 107.15667 97.32233      tau UCVM UCVM
##   Mboth
## 1  UCVM
## 2  UCVM
## 3  UCVM
```

In the output of `selectRACVM`, the `extremes` column indicates which parameter showed significant changes in parameter values defined by no overlap in the 95% confidence interval, and the `dAIC` and `dBIC` compare the change point model to the no change point model. We can ask this functin to tidy the selection table according to any combination of several criteria: by the extreme, by negative dAIC or dBIC, or by a mismatch in the models selected (see the help file).

```
getCPtable(CPs = CP.clustered, Z = Z, T = T, modelset = "UCVM", tidy = "extremes", iterate = TRUE)
```

```
##          CP     start      end      dAIC     dBIC extremes   M1   M2 Mboth
## 1  93.18417  1.181941 180.9432  67.18937 57.30945  eta-tau UCVM UCVM  UCVM
## 2 180.94322 93.184175 275.4742 107.15667 97.32233      tau UCVM UCVM  UCVM
```

We now have a final selected set of change points and, perhaps more usefully, a summary of which parameters precisely changed. Note that thanks to the magic of `magrittr` piping, the process of finding and selecting change points can be reduced to a single line of code, which is convenient for comparing the robustness of the different settings. Thus, replicating the above for several cluster widths yields the same basic result:

```
simSweep %>% findCandidateChangePoints(clusterwidth = 1) %>%
  getCPtable(Z = Z, T = T, modelset = "UCVM")
```

```
##          CP     start      end      dAIC     dBIC extremes   M1   M2 Mboth
## 1  94.57748  1.181941 180.6926  67.18937 57.30945  eta-tau UCVM UCVM  UCVM
## 2 180.69264 94.577483 275.4742 107.15667 97.32233      tau UCVM UCVM  UCVM
```

```
simSweep %>% findCandidateChangePoints(clusterwidth = 4) %>%
  getCPtable(Z = Z, T = T, modelset = "UCVM")
```

```
##          CP     start      end      dAIC     dBIC extremes   M1   M2 Mboth
## 1  93.18417  1.181941 180.9432  67.18937 57.30945  eta-tau UCVM UCVM  UCVM
## 2 180.94322 93.184175 275.4742 107.15667 97.32233      tau UCVM UCVM  UCVM
```

```
simSweep %>% findCandidateChangePoints(clusterwidth = 10) %>%
  getCPtable(Z = Z, T = T, modelset = "UCVM")
```

```
##          CP     start      end      dAIC     dBIC extremes   M1   M2 Mboth
## 1  93.18417  1.181941 180.9432  67.18937 57.30945  eta-tau UCVM UCVM  UCVM
## 2 180.94322 93.184175 275.4742 107.15667 97.32233      tau UCVM UCVM  UCVM
```

Compared to the true values of 88.31 and 181.55, these are excellent estimates. Note, we can also expand our "model set" to include all four CVM model:

```
simSweep %>% findCandidateChangePoints(clusterwidth = 4) %>% getCPtable(Z = Z, T = T, modelset ="all")
```

```
##          CP     start      end      dAIC     dBIC extremes   M1   M2 Mboth
## 1  93.18417  1.181941 180.9432  63.68794 57.10133  eta-tau UCVM UCVM  RCVM
## 2 180.94322 93.184175 275.4742 107.15667 97.32233      tau UCVM UCVM  UCVM
```

But the main model selected in each significant partition is still the UCVM, with the only difference being an additional selection of rotation in a (reject) joint model spanning the first two phases.

5.2.4 Step IV - estimating the full model

The final, straightforward, step is simply to estimate the parameters for the model within each of the phases defined by the selected change points, and thereby obtain a fully parameterized model of the

movement. This is done with the getPhases function, which takes a change point table (i.e. the output of getCPtable]) and returns a named list of phases and the estimated parameters:

```
simCP.table <- simSweep %>% findCandidateChangePoints(clusterwidth = 4) %>%
  getCPtable(Z = Z, T = T, modelset = "UCVM")
simPhases <- getPhases(simCP.table, Z = Z, T = T)

## [1] TRUE
##   phase      start       end model      eta      tau
## 1     I   1.181941  93.18417  UCVM 1.883136 1.253826
## 2    II  93.184175 180.94322  UCVM 5.497431 7.290881
## 3   III 180.943223 275.47416  UCVM 5.587432 1.442225
```

The summarizePhases, getPhaseParameter and plotPhaseParameter functions are useful for obtaining the estimates (with confidence intervals) and plotting their values. In the code below, we plot the track, color it by partition, and illustrate the values of the parameters.

```
  require(gplots)
  cols <- rich.colors(length(simPhases))
  T.cuts <- c(T[1], simCP.table$CP, T[length(T)])
  Z.cols <- cols[cut(T, T.cuts, include.lowest = TRUE)]

  phaseTable <- summarizePhases(simPhases)

## [1] TRUE

  plot(Z, asp=1, type="l", xpd=FALSE)
  points(Z, col=Z.cols, pch=21, bg = alpha(Z.cols, 0.5), cex=0.8)
  legend("topright", legend = paste0(phaseTable$phase, ": ", phaseTable$model),
         fill=cols, ncol=1, bty="n", title = "Phase: model")

  par(mar=c(1,0,1,0), xpd=NA)
  plotPhaseParameter("tau", simPhases, ylab="time units", xaxt="n", xlab="", col=cols, log="y")
  plotPhaseParameter("eta", simPhases,  ylab="distance / time", xlab="time", col=cols)
```

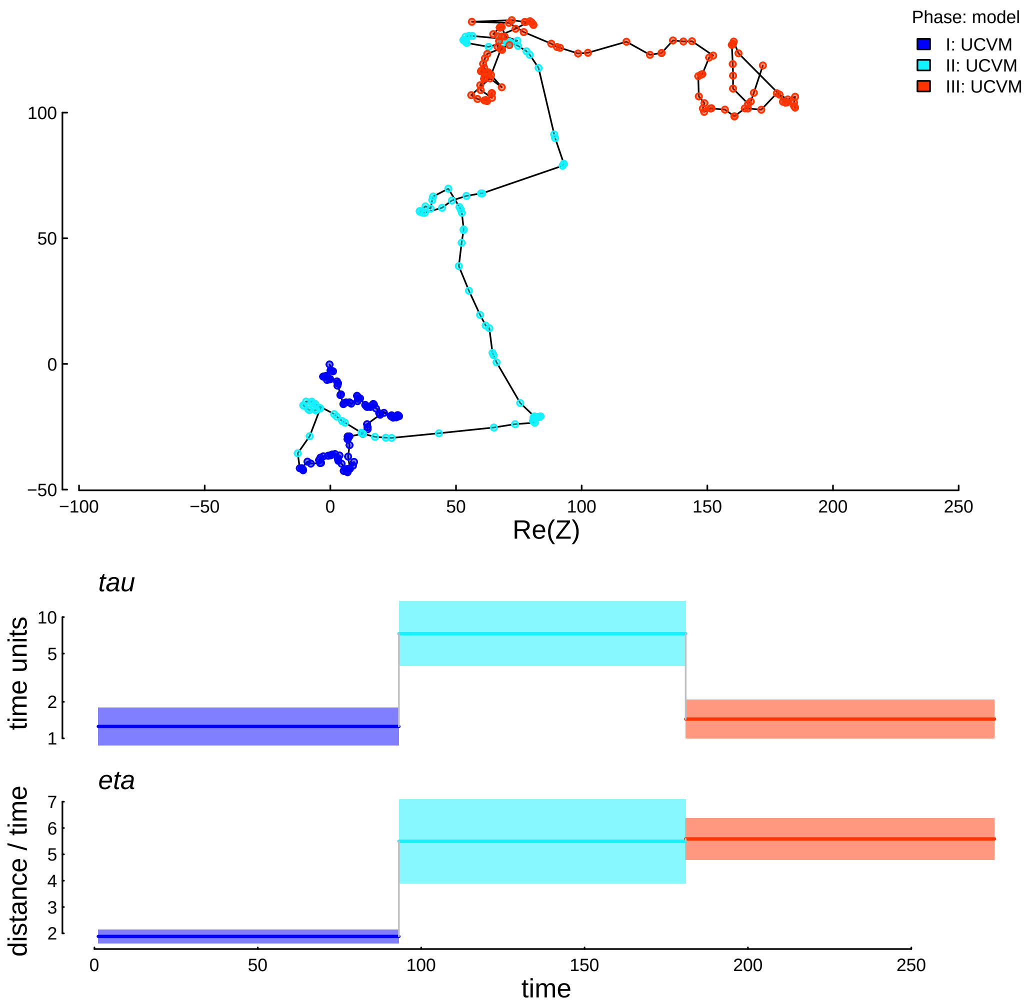

The estimates are fairly accurate with respect to the true τ and η values (1-5-1 and 2-5-5) respectively.

6 Kestrel data BCPA

We perform this analysis now on a portion of the kestrel data analyzed in the Gurarie et al. (in review) manuscript. The basic steps are the same as above, but the selection occurs across all four models. The portion of data we analyze is:

```
data(Kestrel)
k <- Kestrel[3730:4150,]
head(k)
```

```
##          event.id visible           timestamp longitude latitude
## 91352 274467264    true 2012-05-15 16:40:31 -6.556618 37.39312
## 91353 274467265    true 2012-05-15 16:40:32 -6.556670 37.39305
## 91354 274467266    true 2012-05-15 16:40:33 -6.556688 37.39297
## 91355 274467267    true 2012-05-15 16:40:34 -6.556680 37.39283
```

```
## 91356 274467268    true 2012-05-15 16:40:35 -6.556643 37.39268
## 91357 274467269    true 2012-05-15 16:40:36 -6.556557 37.39256
##       manually.marked.outlier sensor.type individual.taxon.canonical.name
## 91352                                 gps                   Falco naumanni
## 91353                                 gps                   Falco naumanni
## 91354                                 gps                   Falco naumanni
## 91355                                 gps                   Falco naumanni
## 91356                                 gps                   Falco naumanni
## 91357                                 gps                   Falco naumanni
##       tag.local.identifier     ID          study.name         X       Y
## 91352                  457 B[6.X] Lesser Kestrels EBD 716295.5 4141286
## 91353                  457 B[6.X] Lesser Kestrels EBD 716291.1 4141278
## 91354                  457 B[6.X] Lesser Kestrels EBD 716289.8 4141269
## 91355                  457 B[6.X] Lesser Kestrels EBD 716290.9 4141254
## 91356                  457 B[6.X] Lesser Kestrels EBD 716294.6 4141237
## 91357                  457 B[6.X] Lesser Kestrels EBD 716302.5 4141224

with(k, scan.track(x=X, y=Y))
```

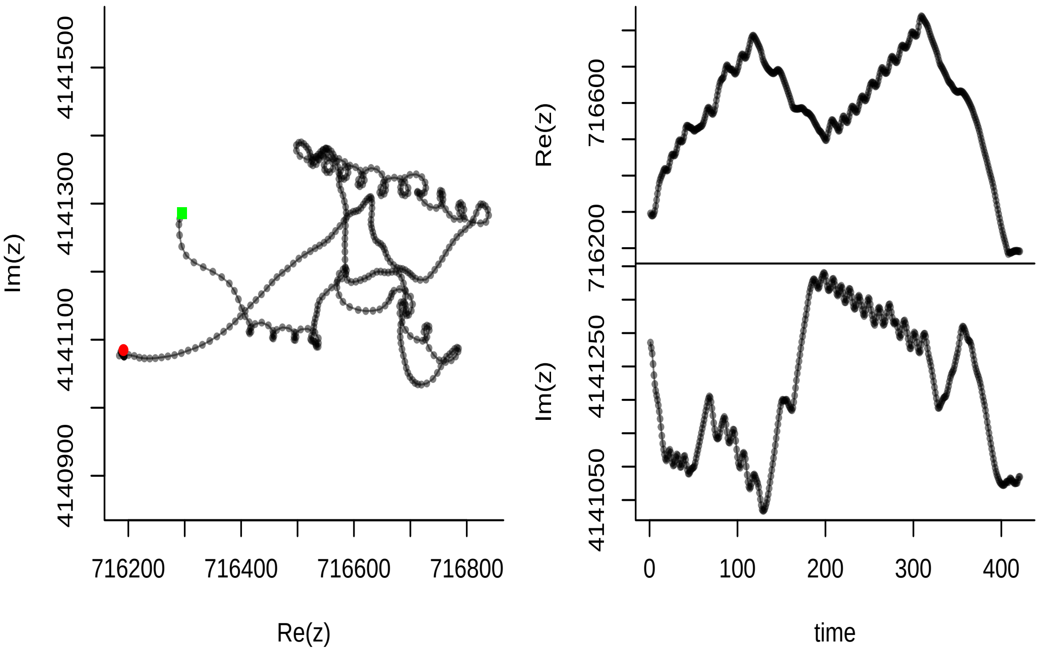

The window sweep can take some time. Note that we convert the time to seconds from start (in this case, exactly 420 seconds), which is somewhat more convenient for the analysis (though feeding POSIX objects will also work).

```
k$T <- as.numeric(k$timestamp - min(k$timestamp))
k.sweep <- with(k, sweepRACVM(XY=cbind(X,Y), T=T, windowsize = 50, windowstep = 5, model = "RACVM", time.units
```

Note that the time data are POSIX objects, and it is important to specify the time units of the analysis (in this case, seconds). We plot the relative likelihoods:

```r
plotWindowSweep(k.sweep)
```

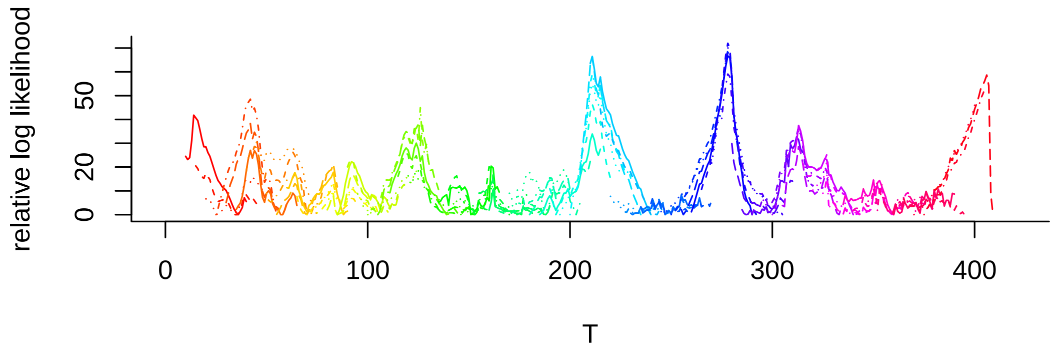

There are several clear peaks corresponding to likely change points. We find candidate change points:

```r
k.CPs <- findCandidateChangePoints(windowsweep = k.sweep, clusterwidth = 2)
```

Note that the selected cluster width (2) was the minimum one that did not produce a warning for clusters that were "too close". We now iteratively test all of the change points for parameter values and model changes. Specifying `modelset = 'all'` is a shortcut for `modelset = c('UCVM', 'ACVM', 'RCVM', 'RACVM')`:

```r
XY <- cbind(k$X, k$Y)
k.CPtable <- getCPtable(CPs = k.CPs, XY = XY, T = k$T,
            modelset = "all", tidy = c("extremes"), iterate = FALSE, spline=TRUE)
```

Note that in this implementation, we chose the option `spline=TRUE`, as the regularity and apparent precision of these data and the curvature of the track suggest that the spline approximation will give improved estimates of the parameters.

```r
Z <- XY[,1] + 1i*XY[,2]
k.phases <- getPhases(k.CPtable, T=k$T, Z=Z, verbose=FALSE)
summarizePhases(k.phases)

## [1] FALSE
##    phase    start      end model      eta       mu.x        mu.y         tau       omega
## 1      I   0.0000  14.5000  ACVM 5.134427 11.261642 -11.8672904   3.3547568          NA
## 2     II  14.5000  43.0000 RACVM 7.558733  4.563634  -0.8733237  80.0370013  0.72948891
## 3    III  43.0000  83.0000 RACVM 7.234778  3.164869   2.8879738  16.1918966 -0.21836126
## 4     IV  83.0000  92.0000 RACVM 5.385657  2.479384  -2.0118591  35.9808688  0.62230177
## 5      V  92.0000 124.8333 RACVM 8.717702  3.091361  -3.0767722  25.8196192 -0.44562541
## 6     VI 124.8333 191.0000  UCVM 7.125503        NA          NA  24.7488758          NA
## 7    VII 191.0000 210.8571  RCVM 8.496581        NA          NA  16.8598201 -0.40791531
## 8   VIII 210.8571 255.0000 RACVM 7.061061  2.846705  -1.0539028  40.1353836  0.59172459
## 9     IX 255.0000 278.2857 RACVM 9.649773  2.999946   0.1531566 163.8918866  0.55457148
## 10     X 278.2857 312.8333 RACVM 9.054524  3.459657  -0.7632422  21.8552904 -0.53171439
## 11    XI 312.8333 327.0000 RACVM 1.819282 -7.211241  -5.9798120   5.9448499  0.27339664
## 12   XII 327.0000 351.4000  RCVM 5.033194        NA          NA  21.0032120  0.11583676
## 13  XIII 351.4000 381.5000  ACVM 2.142204 -7.784177  -6.8648865   5.3203728          NA
## 14   XIV 381.5000 405.5000  RCVM 4.710963        NA          NA  76.4658816  0.03997513
## 15    XV 405.5000 420.0000  UCVM 2.760968        NA          NA   0.0738589          NA
```

```r
  cols <- rich.colors(length(k.phases))
  T.cuts <- c(k$T[1], k.CPtable$CP, k$T[length(k$T)])
```

```r
  Z.cols <- cols[cut(k$T, T.cuts, include.lowest = TRUE)]

  phaseTable <- summarizePhases(k.phases)

## [1] FALSE

  plot(Z, asp=1, type="l", xpd=FALSE, xlab="X (m)")
  points(Z, col=Z.cols, pch=21, bg = alpha(Z.cols, 0.5), cex=0.8)
  legend("topleft", legend = paste0(phaseTable$phase, ": ", phaseTable$model),
         fill=cols, ncol=2, bty="n", title = "Phase: model")

  par(mar=c(1,0,1,0), xpd=NA)
  plotPhaseParameter("tau", k.phases, ylab="sec", xaxt="n", xlab="", col=cols, log="y")
  plotPhaseParameter("eta", k.phases,  ylab="m / sec", xlab="time", col=cols)
  plotPhaseParameter("omega", k.phases,  ylab="rad / sec", xlab="time", col=cols)
  plotPhaseParameter("mu.x", k.phases,  ylab="m / sec", xlab="time", col=cols)
  plotPhaseParameter("mu.y", k.phases,  ylab="m / sec", xlab="time", col=cols)
```

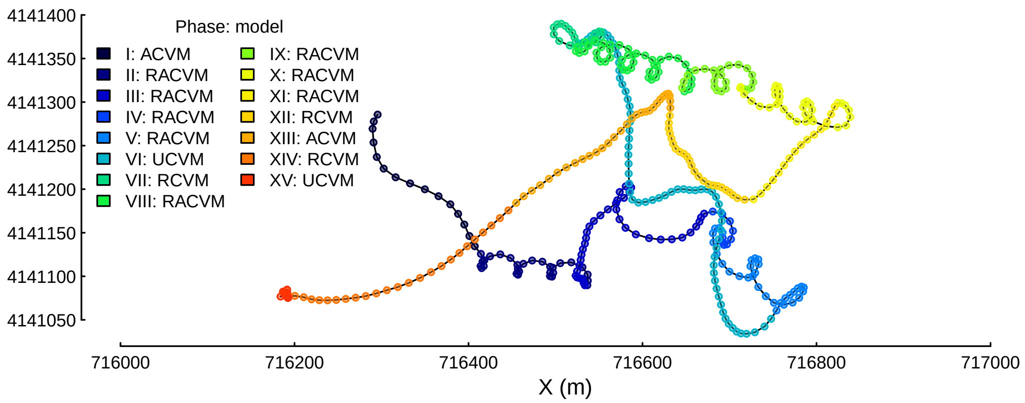

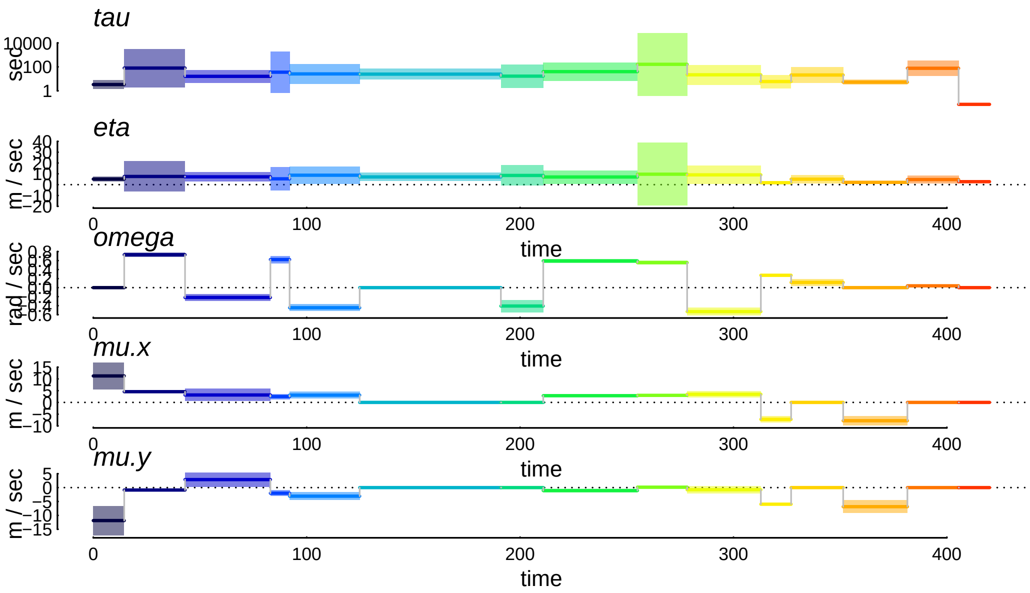

References

- Gurarie, E., C. Fleming, K. Laidre, W. Fagan, O. Ovaskainen (in review) *Methods in Ecology and Evolution*.
- Johnson, D., J. London, M. -A. Lea, and J. Durban (2008) Continuous-time correlated random walk model for animal telemetry data. Ecology 89(5) 1208-1215.
- Hernández-Pliego, J., C. Rodríguez, J. Bustamante (2015) 'Data from: Why do kestrels soar?', Movebank Data Repository. https://www.datarepository.movebank.org/handle/10255/move.486
- Hernández-Pliego, J., C. Rodríguez, J. Bustamante (2015) 'Why do kestrels soar?', PLoS ONE 10(12).
