# Supplementary material for: Correlated velocity models as a fundamental unit of animal movement: synthesis and applications
Source: Mov Ecol. 2017 May 10;5:13. doi: 10.1186/s40462-017-0103-3 (PMC5424322; doi:10.1186/s40462-017-0103-3)
Supplement: Supplementary file 3 — The source bundle for the smoove package. (GZ 7034.88 kb) [file 40462_2017_103_MOESM3_ESM.gz › smoove/vignettes/bibliography.rtf]

Hernández-Pliego, J.; Rodríguez, C. & Bustamante, J. (2015), 'Data from: Why do kestrels soar?', Movebank Data Repository.

Hernández-Pliego, J.; Rodríguez, C. & Bustamante, J. (2014), 'Gone with the wind: Seasonal trends in foraging movement directions for a central place forager.', Current Zoology 60(604-615).

Hernández-Pliego, J.; Rodríguez, C. & Bustamante, J. (2015), 'Why do kestrels soar?', PLoS ONE 10(12).
