# Supplementary figures and images for: Correlated velocity models as a fundamental unit of animal movement: synthesis and applications
Source: Mov Ecol. 2017 May 10;5:13. doi: 10.1186/s40462-017-0103-3 (PMC5424322; doi:10.1186/s40462-017-0103-3)

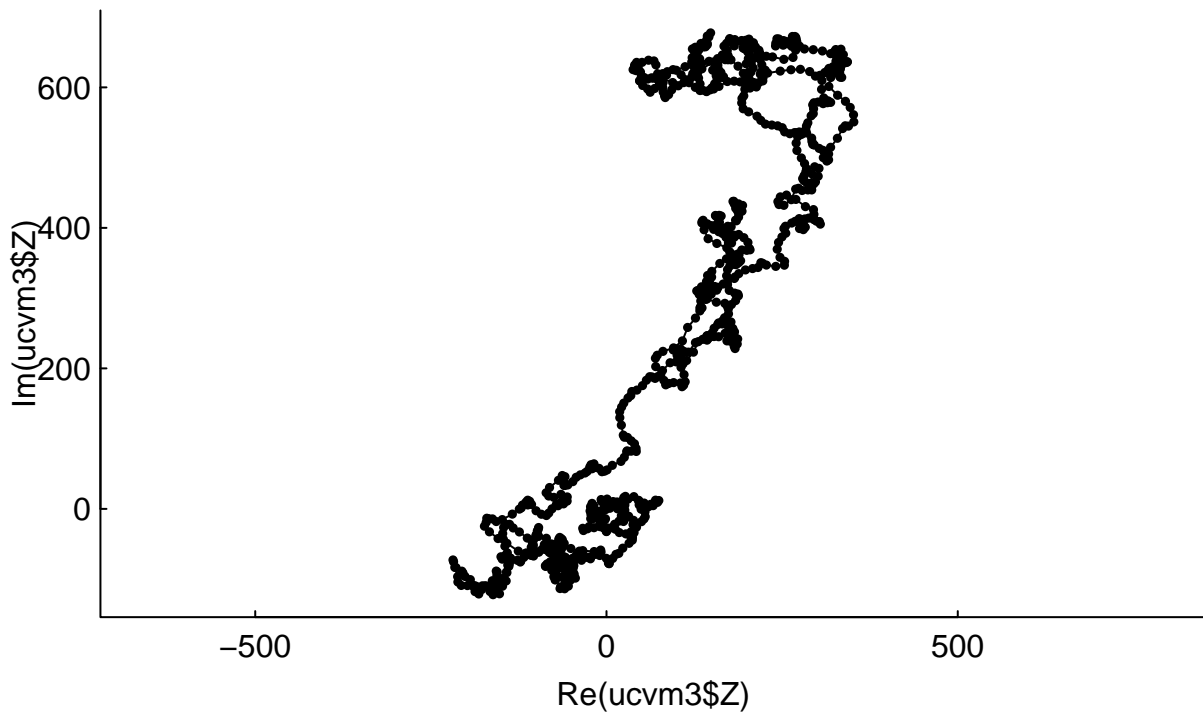

Supplement: Supplementary file 3 — The source bundle for the smoove package. (GZ 7034.88 kb) [file 40462_2017_103_MOESM3_ESM.gz › smoove/vignettes/figure/CRW-1.pdf]

**Autocorrelations should be near 0 at lag>0**

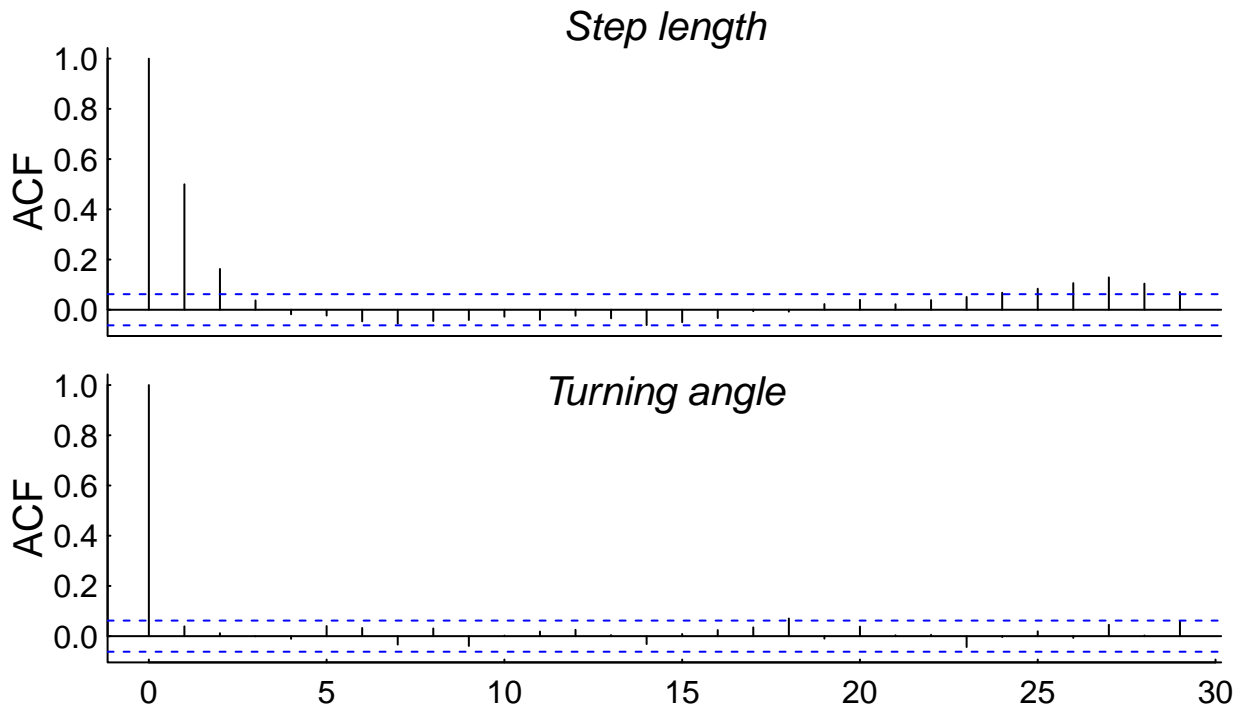

Supplement: Supplementary file 3 — The source bundle for the smoove package. (GZ 7034.88 kb) [file 40462_2017_103_MOESM3_ESM.gz › smoove/vignettes/figure/CRW-2.pdf]

**UCVM**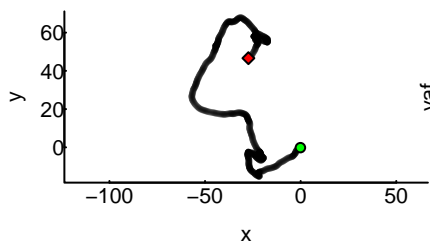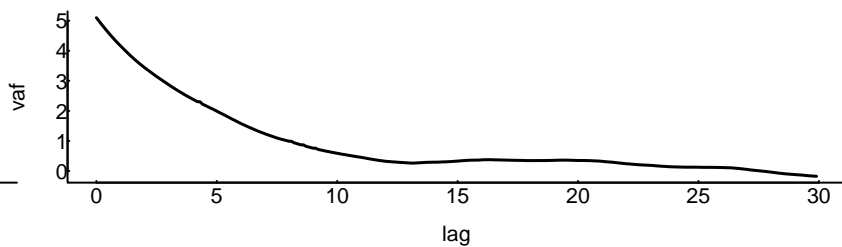**ACVM**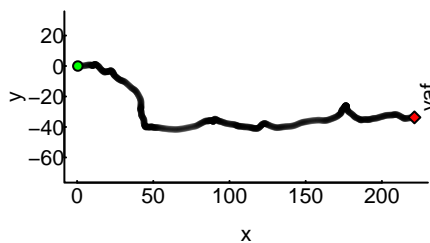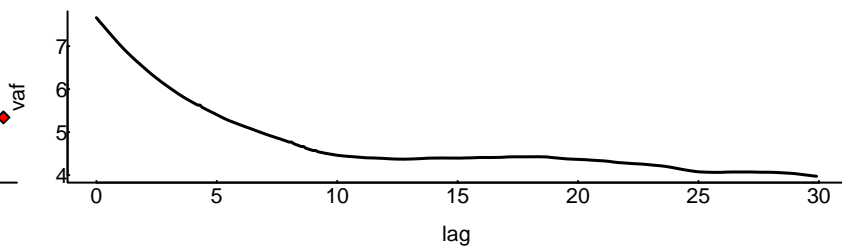**RCVM**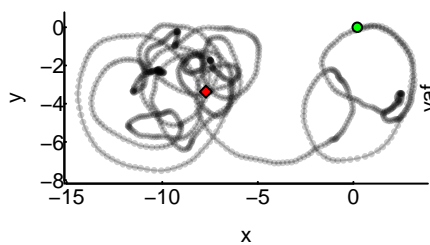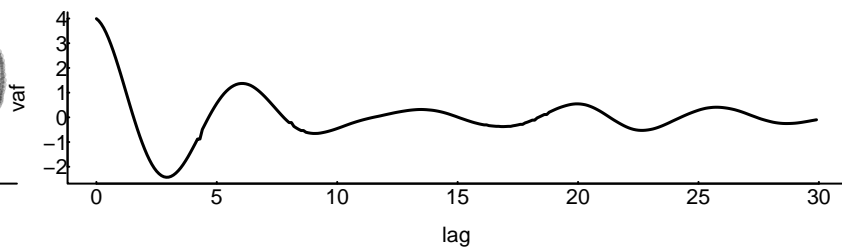**RACVM**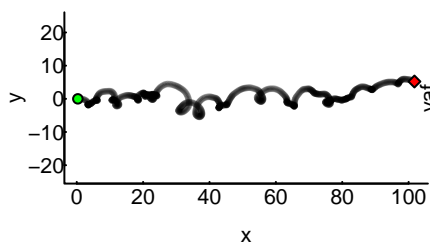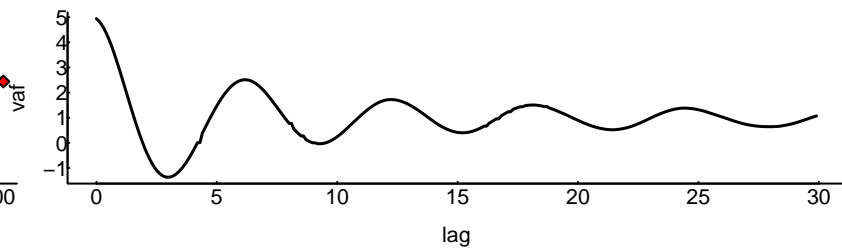

Supplement: Supplementary file 3 — The source bundle for the smoove package. (GZ 7034.88 kb) [file 40462_2017_103_MOESM3_ESM.gz › smoove/vignettes/figure/EVAFplots-1.pdf]

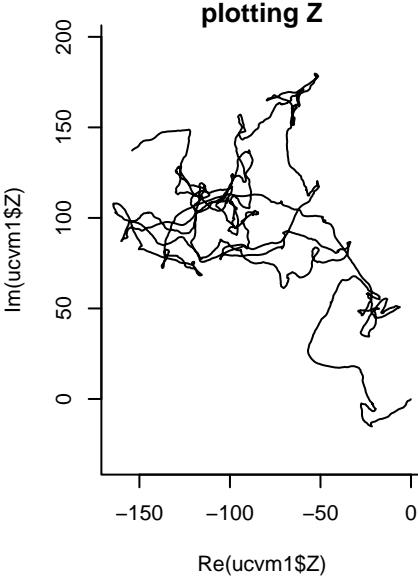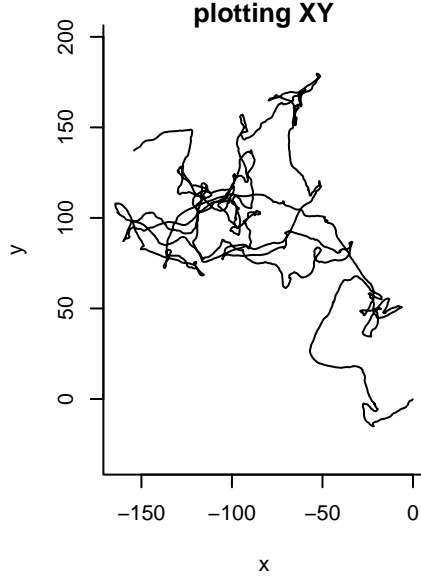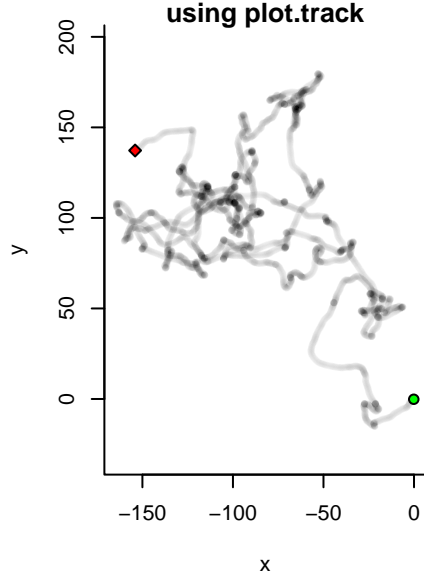

Supplement: Supplementary file 3 — The source bundle for the smoove package. (GZ 7034.88 kb) [file 40462_2017_103_MOESM3_ESM.gz › smoove/vignettes/figure/FirstPlot-1.pdf]

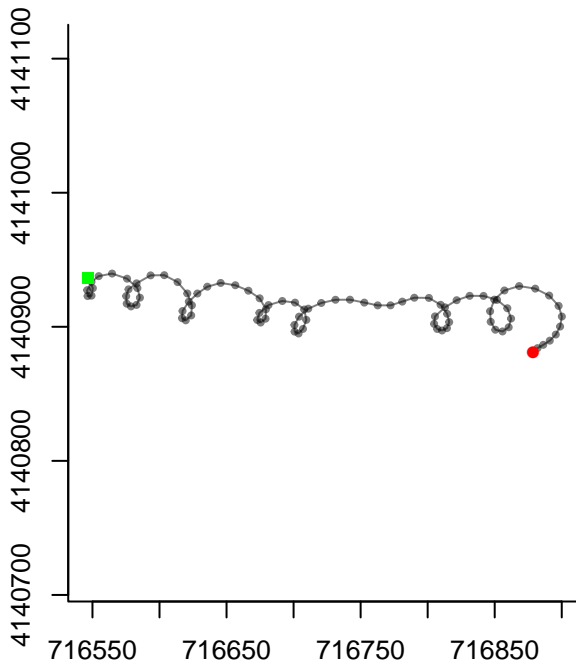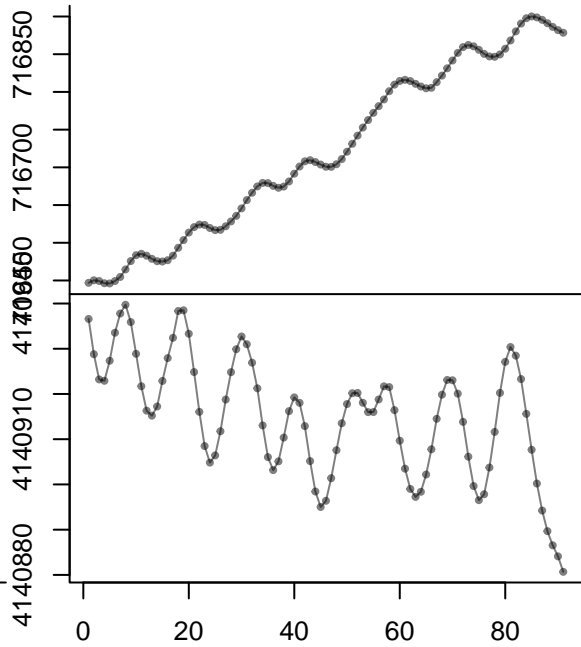

Supplement: Supplementary file 3 — The source bundle for the smoove package. (GZ 7034.88 kb) [file 40462_2017_103_MOESM3_ESM.gz › smoove/vignettes/figure/K1-1.pdf]

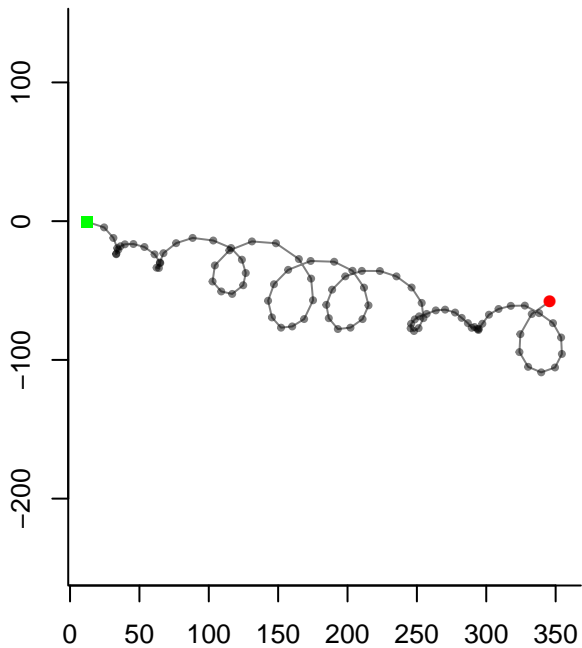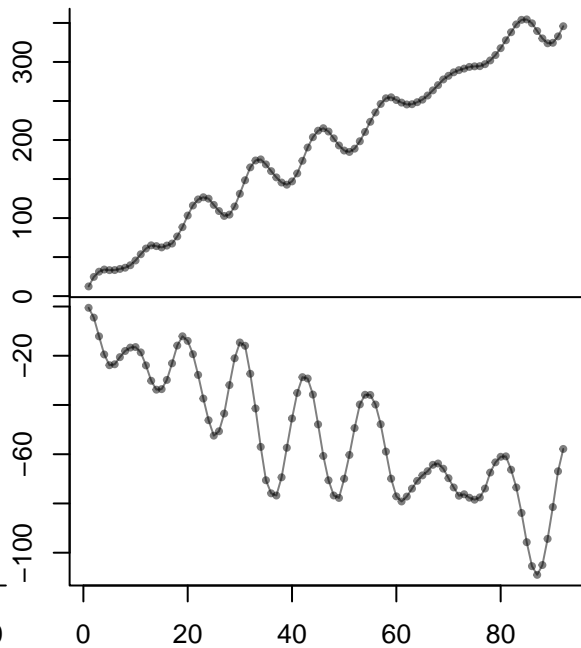

Supplement: Supplementary file 3 — The source bundle for the smoove package. (GZ 7034.88 kb) [file 40462_2017_103_MOESM3_ESM.gz › smoove/vignettes/figure/K1_sim-1.pdf]

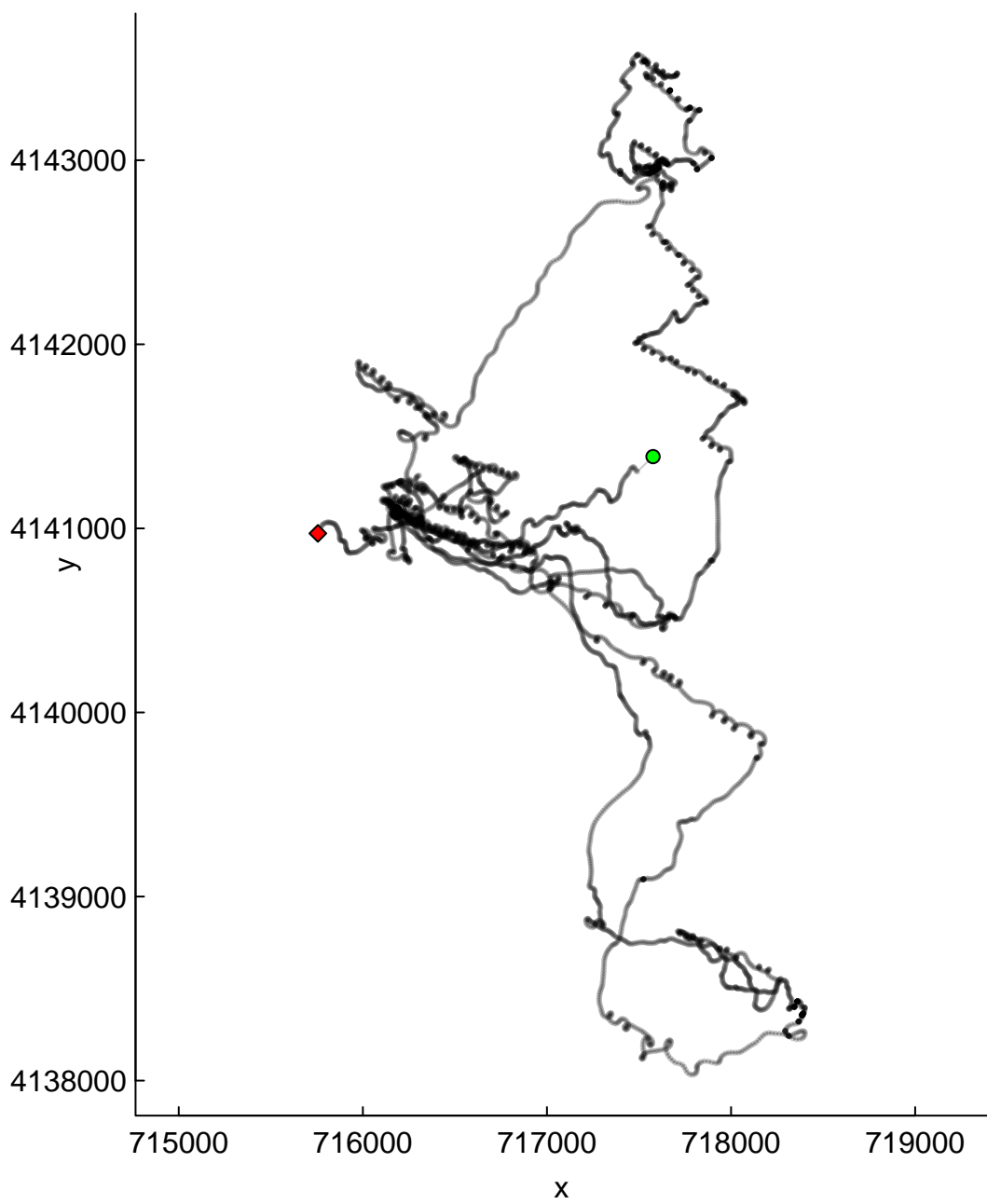

Supplement: Supplementary file 3 — The source bundle for the smoove package. (GZ 7034.88 kb) [file 40462_2017_103_MOESM3_ESM.gz › smoove/vignettes/figure/KestrelFlight-1.pdf]

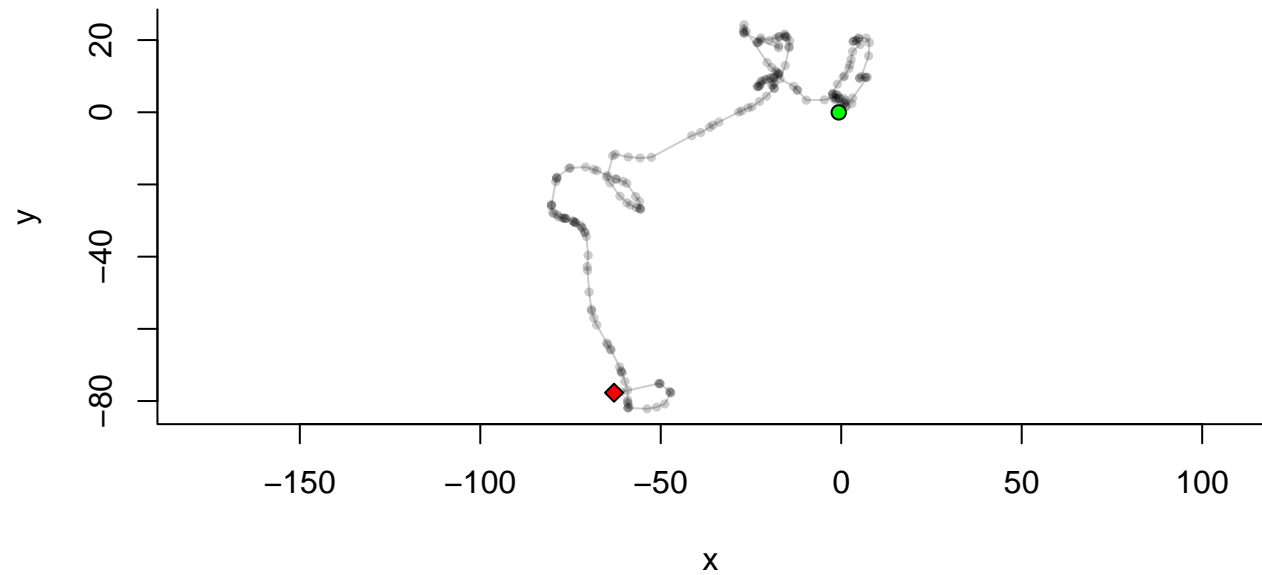

Supplement: Supplementary file 3 — The source bundle for the smoove package. (GZ 7034.88 kb) [file 40462_2017_103_MOESM3_ESM.gz › smoove/vignettes/figure/TwoPhaseCVM-1.pdf]

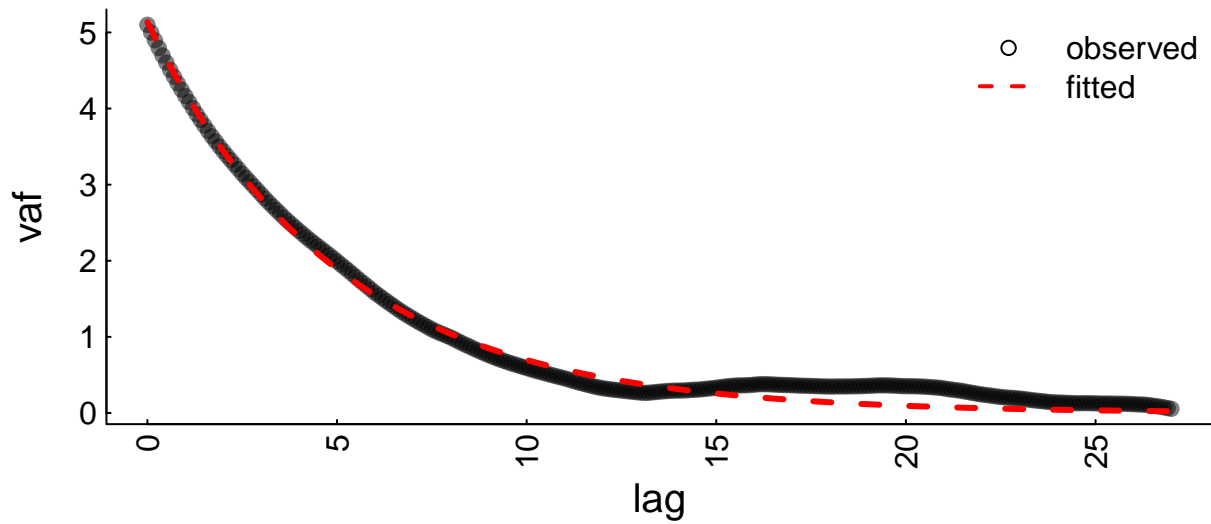

Supplement: Supplementary file 3 — The source bundle for the smoove package. (GZ 7034.88 kb) [file 40462_2017_103_MOESM3_ESM.gz › smoove/vignettes/figure/VAF1-1.pdf]

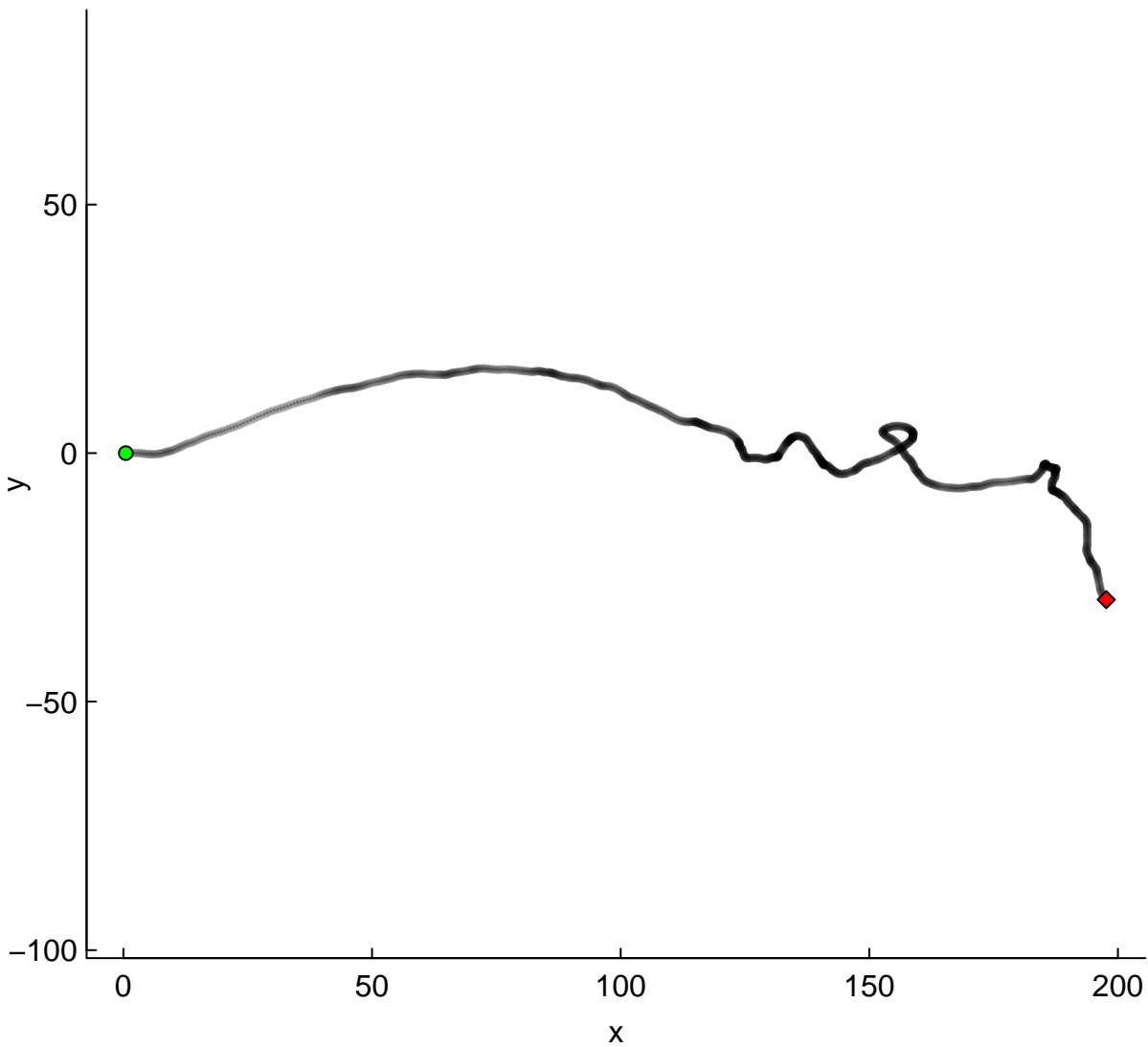

Supplement: Supplementary file 3 — The source bundle for the smoove package. (GZ 7034.88 kb) [file 40462_2017_103_MOESM3_ESM.gz › smoove/vignettes/figure/acvm-1.pdf]

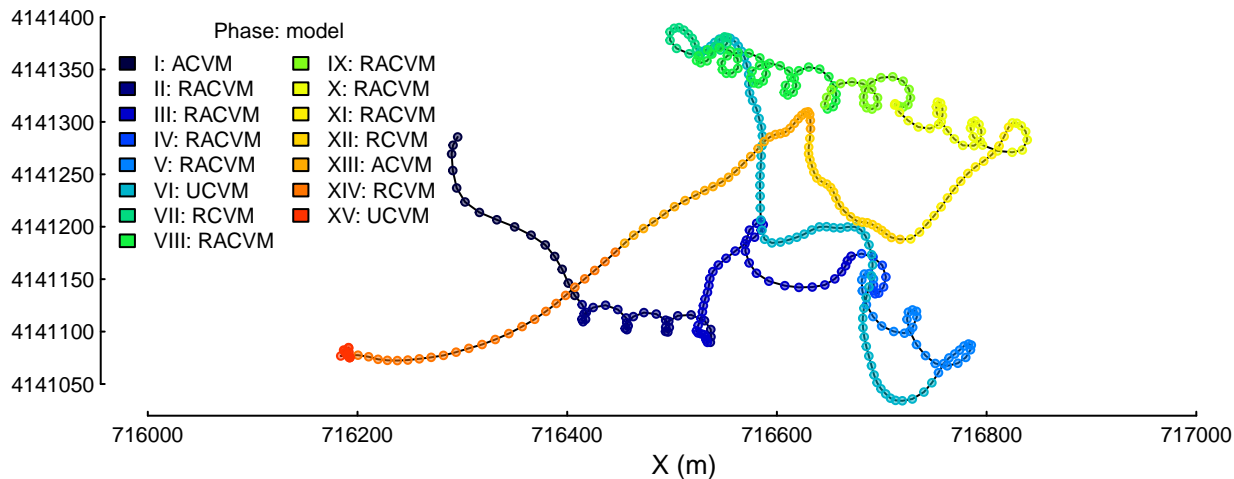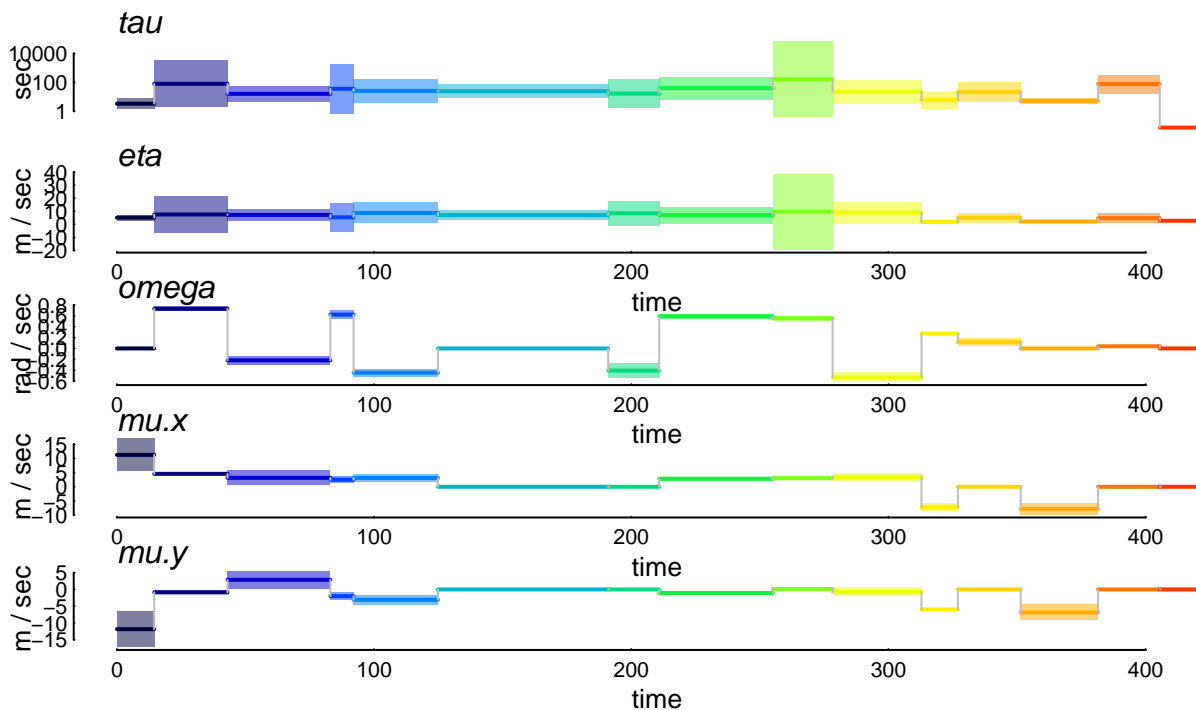

Supplement: Supplementary file 3 — The source bundle for the smoove package. (GZ 7034.88 kb) [file 40462_2017_103_MOESM3_ESM.gz › smoove/vignettes/figure/kestrelMegaPlot-1.pdf]

relative log likelihood

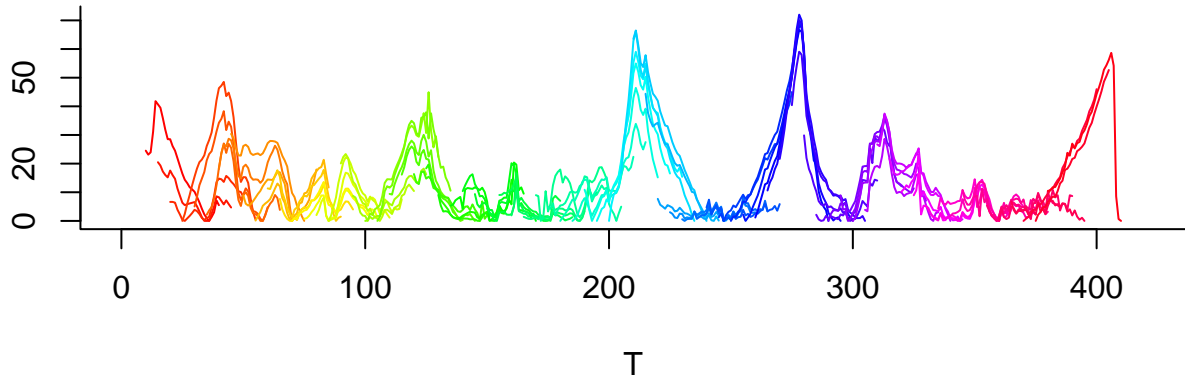

Supplement: Supplementary file 3 — The source bundle for the smoove package. (GZ 7034.88 kb) [file 40462_2017_103_MOESM3_ESM.gz › smoove/vignettes/figure/plotKestrelSweep-1.pdf]

**RCVM**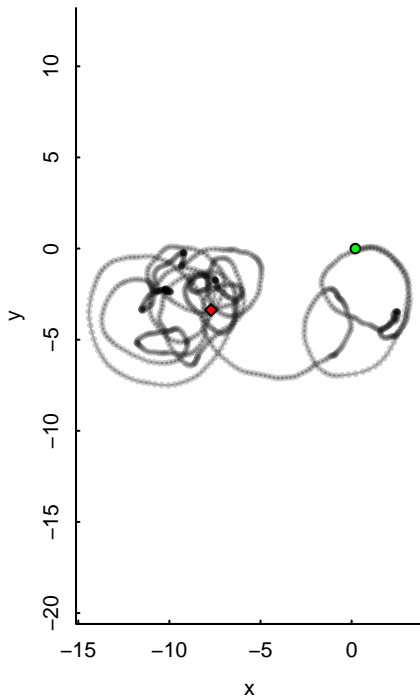**ACVM**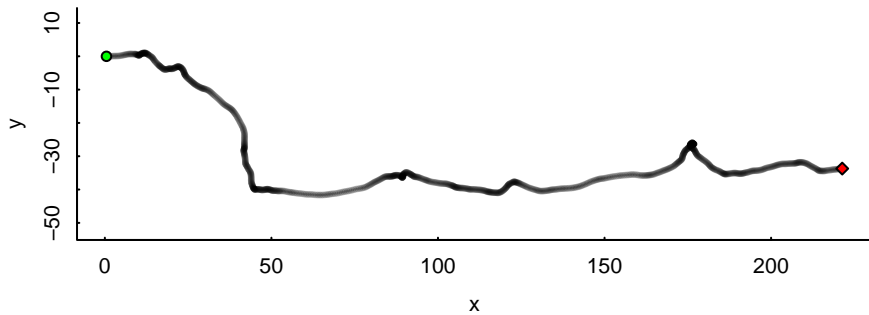**RACVM**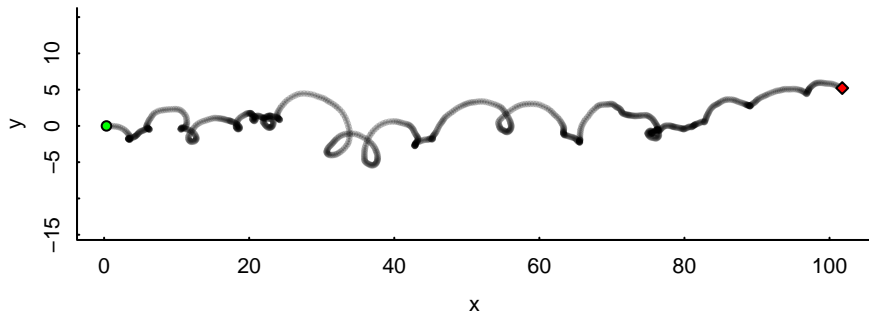

Supplement: Supplementary file 3 — The source bundle for the smoove package. (GZ 7034.88 kb) [file 40462_2017_103_MOESM3_ESM.gz › smoove/vignettes/figure/plotRACVM-1.pdf]

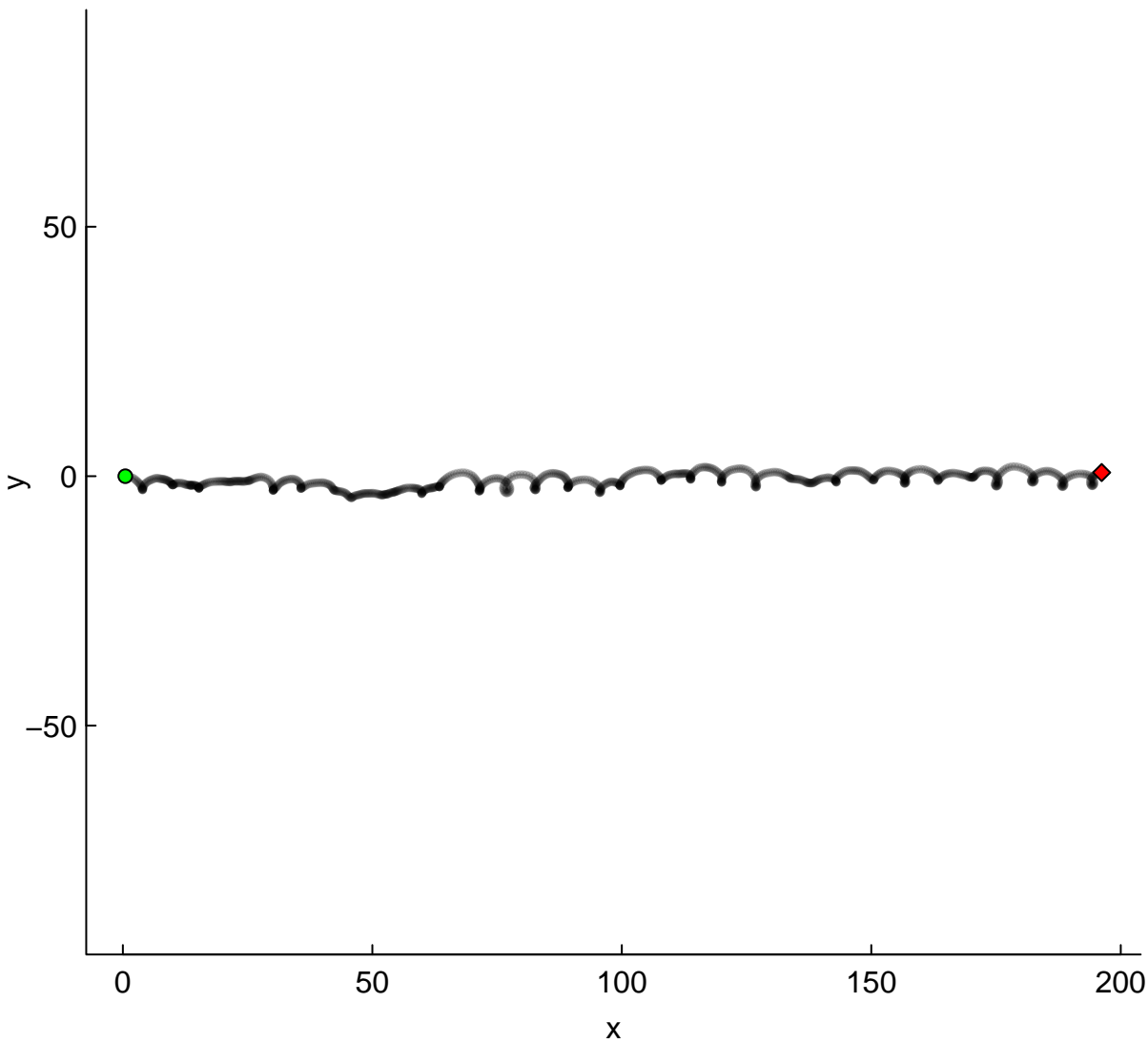

Supplement: Supplementary file 3 — The source bundle for the smoove package. (GZ 7034.88 kb) [file 40462_2017_103_MOESM3_ESM.gz › smoove/vignettes/figure/racvm-1.pdf]

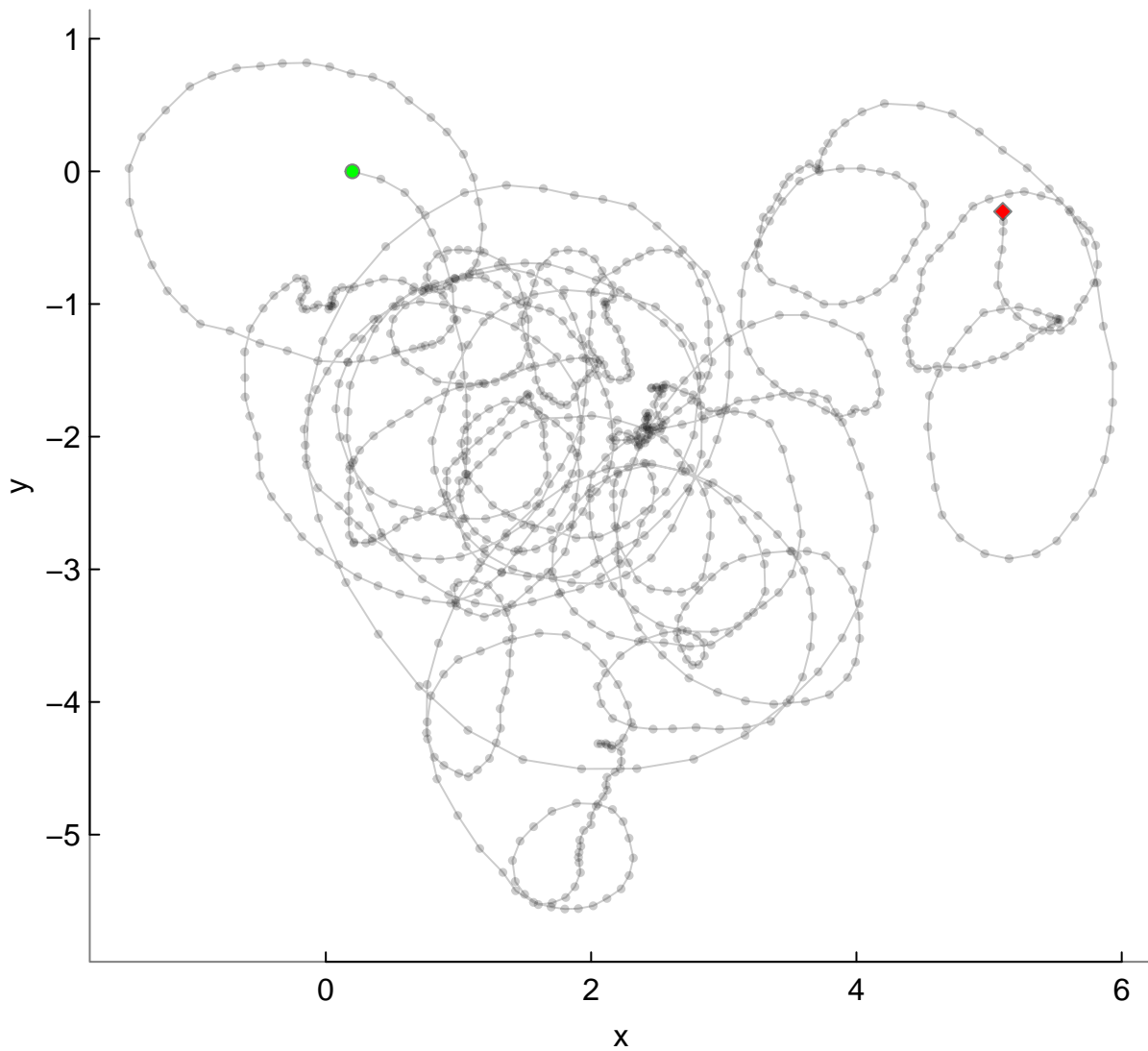

Supplement: Supplementary file 3 — The source bundle for the smoove package. (GZ 7034.88 kb) [file 40462_2017_103_MOESM3_ESM.gz › smoove/vignettes/figure/rcvm-1.pdf]

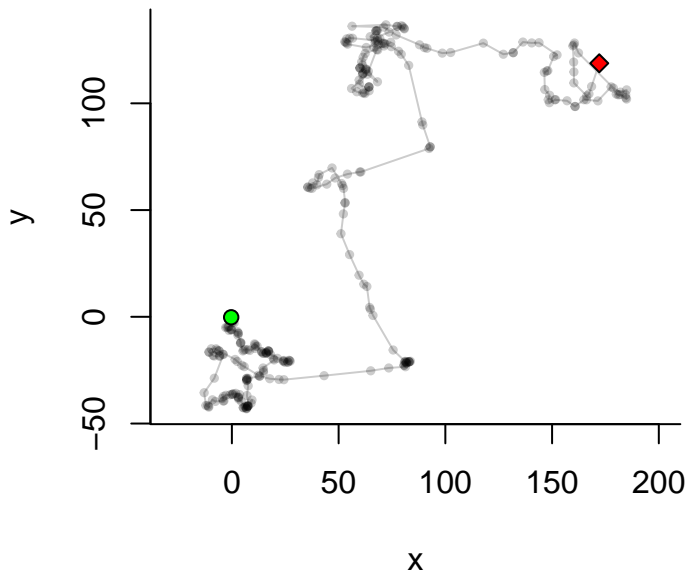

Supplement: Supplementary file 3 — The source bundle for the smoove package. (GZ 7034.88 kb) [file 40462_2017_103_MOESM3_ESM.gz › smoove/vignettes/figure/simSweepGenerate-1.pdf]

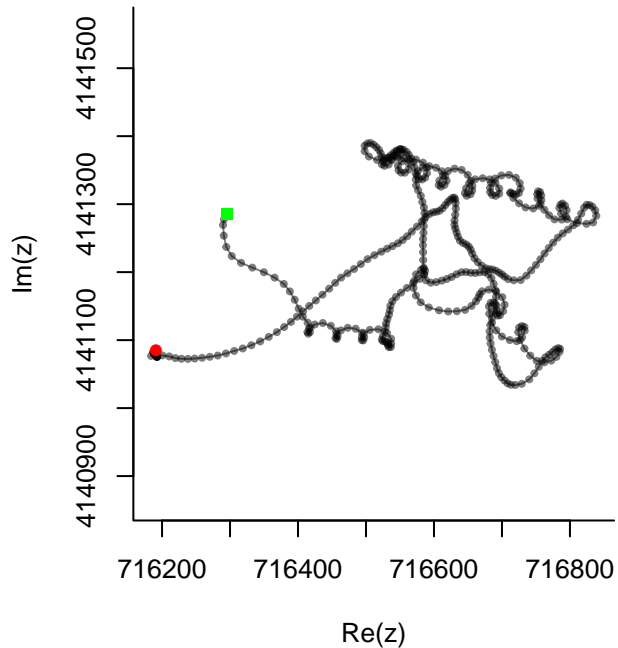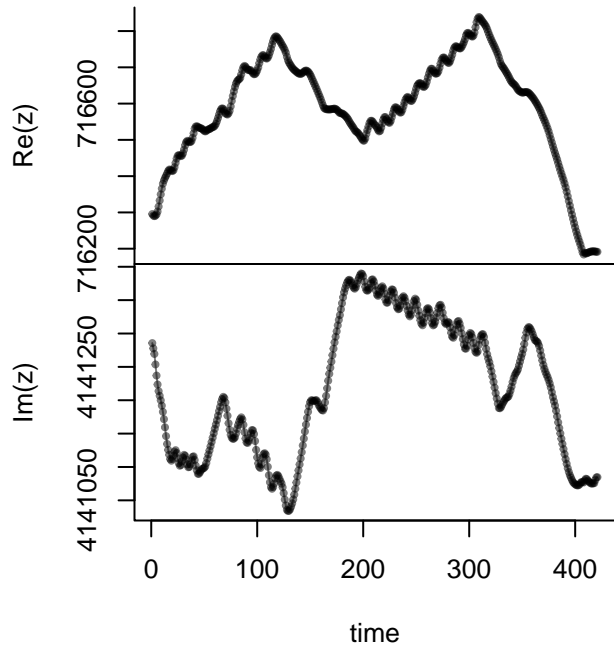

Supplement: Supplementary file 3 — The source bundle for the smoove package. (GZ 7034.88 kb) [file 40462_2017_103_MOESM3_ESM.gz › smoove/vignettes/figure/subsetKestrel-1.pdf]

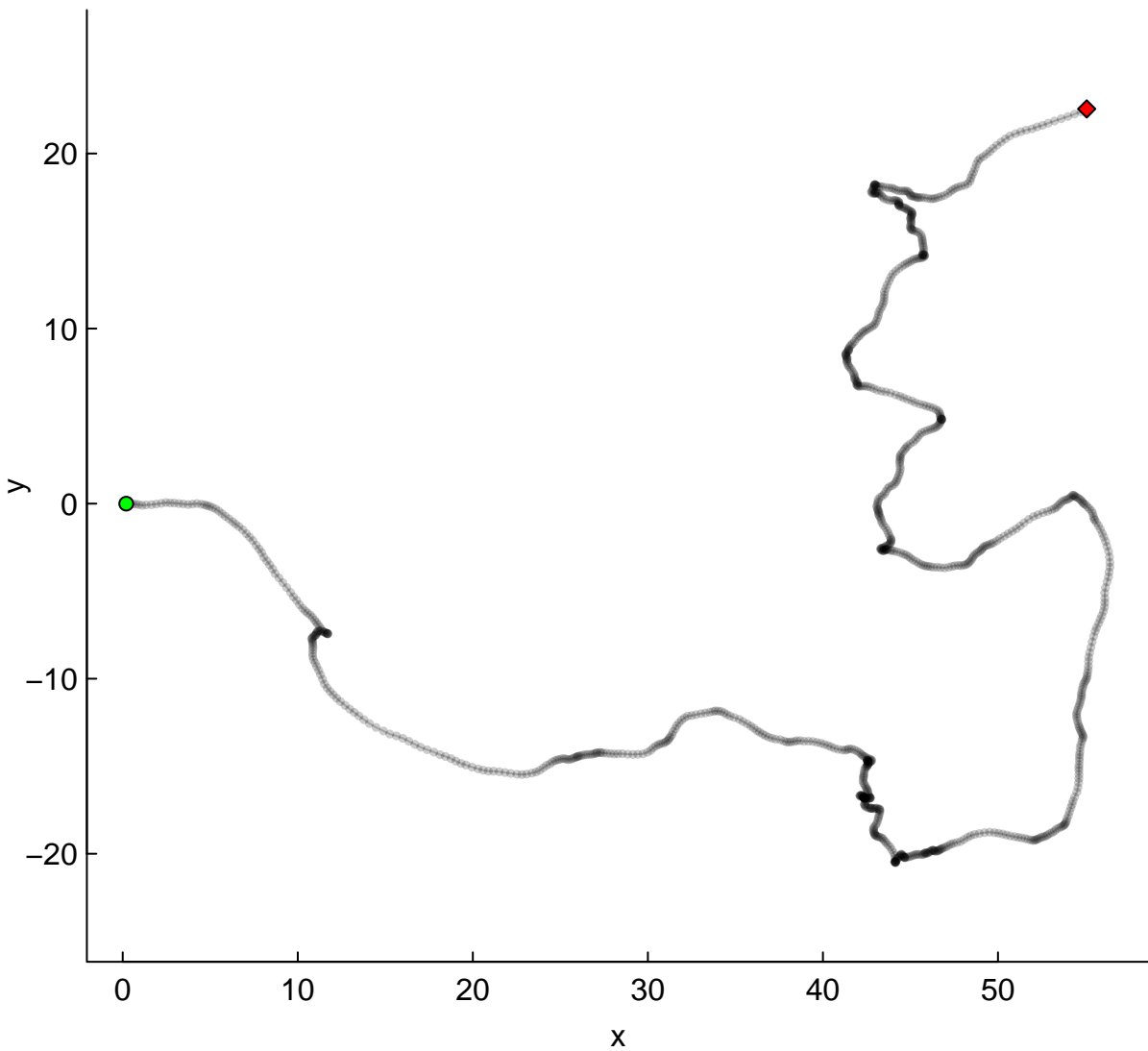

Supplement: Supplementary file 3 — The source bundle for the smoove package. (GZ 7034.88 kb) [file 40462_2017_103_MOESM3_ESM.gz › smoove/vignettes/figure/ucvm-1.pdf]

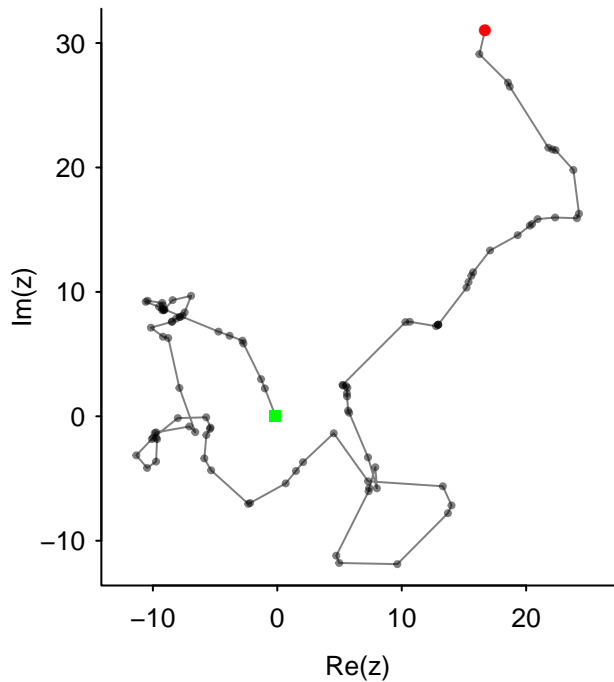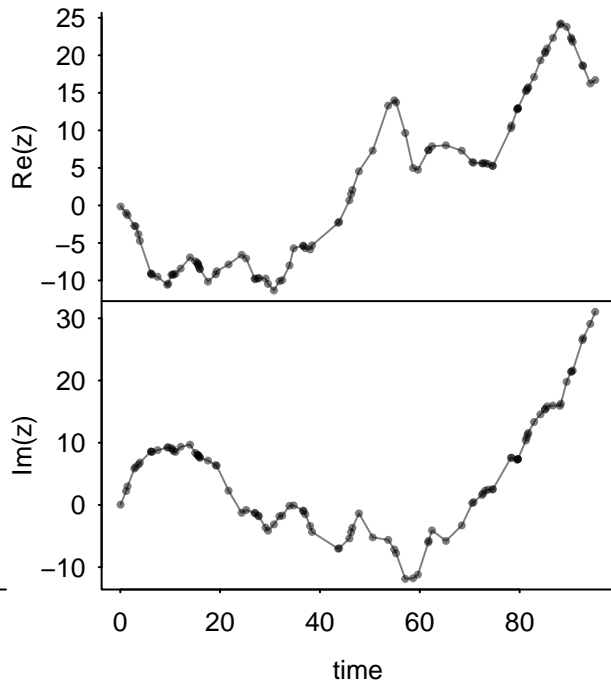

Supplement: Supplementary file 3 — The source bundle for the smoove package. (GZ 7034.88 kb) [file 40462_2017_103_MOESM3_ESM.gz › smoove/vignettes/figure/ucvm_exact-1.pdf]

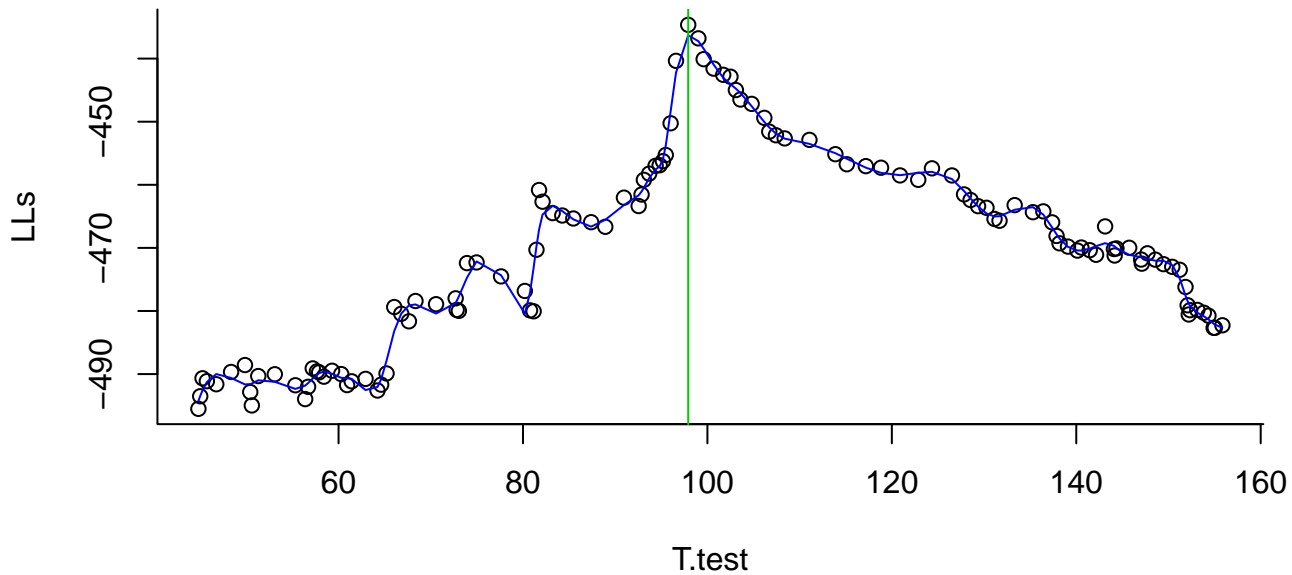

Supplement: Supplementary file 3 — The source bundle for the smoove package. (GZ 7034.88 kb) [file 40462_2017_103_MOESM3_ESM.gz › smoove/vignettes/figure/unnamed-chunk-11-1.pdf]

relative log likelihood

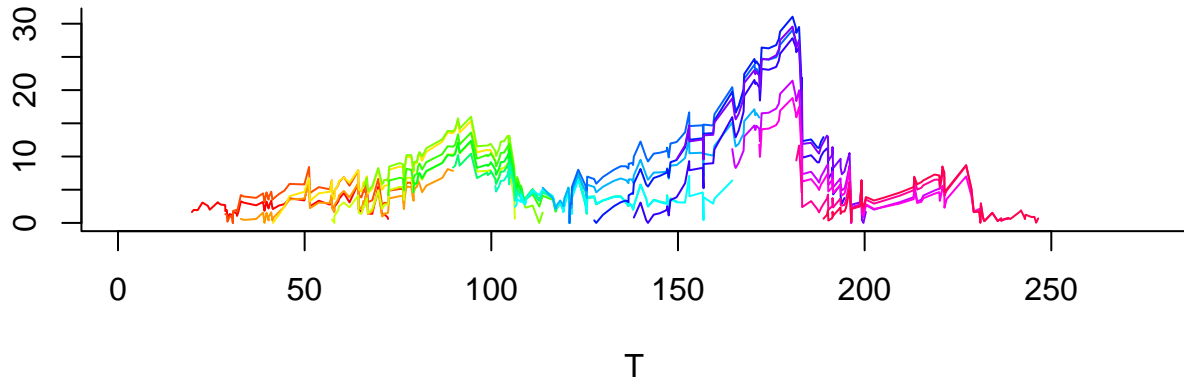

Supplement: Supplementary file 3 — The source bundle for the smoove package. (GZ 7034.88 kb) [file 40462_2017_103_MOESM3_ESM.gz › smoove/vignettes/figure/unnamed-chunk-14-1.pdf]

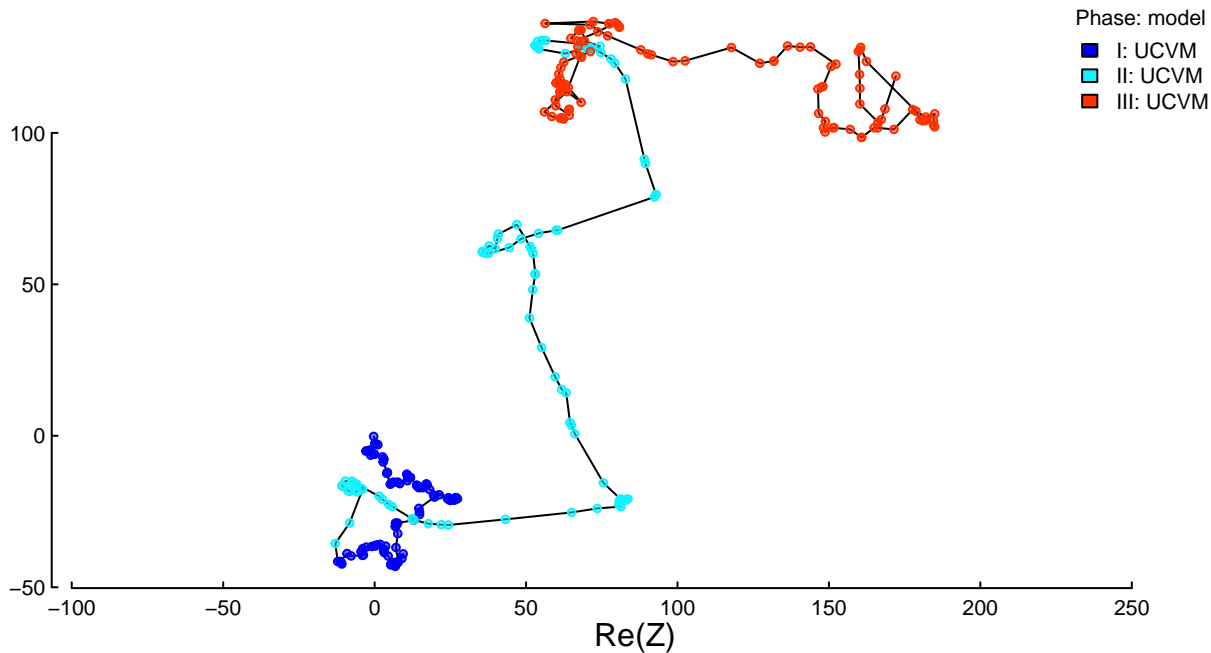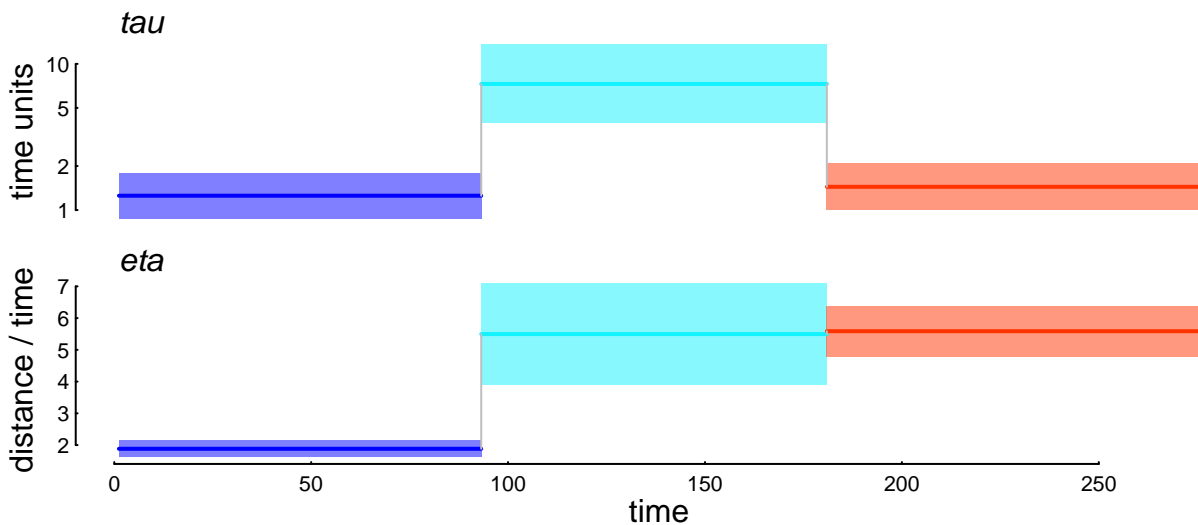

Supplement: Supplementary file 3 — The source bundle for the smoove package. (GZ 7034.88 kb) [file 40462_2017_103_MOESM3_ESM.gz › smoove/vignettes/figure/unnamed-chunk-18-1.pdf]
